# Facile Synthesis of Sulfonyl Chlorides/Bromides from Sulfonyl Hydrazides

**DOI:** 10.3390/molecules26185551

**Published:** 2021-09-13

**Authors:** Rongxiang Chen, Shaohong Xu, Fumin Shen, Canran Xu, Kaikai Wang, Zhanyong Wang, Lantao Liu

**Affiliations:** 1 School of Pharmacy, Xinxiang University, Xinxiang 453000, China; chenrx@xxu.edu.cn (R.C.); ayssfm@163.com (F.S.); xucanran2003@163.com (C.X.); wangkaikaii@163.com (K.W.); zhanyongw@126.com (Z.W.); 2 College of Chemistry and Chemical Engineering, Shangqiu Normal University, Shangqiu 476000, China

**Keywords:** sulfonyl chlorides, sulfonyl bromides, sulfonyl hydrazides, NCS, NBS

## Abstract

A simple and rapid method for efficient synthesis of sulfonyl chlorides/bromides from sulfonyl hydrazide with NXS (X = Cl or Br) and late-stage conversion to several other functional groups was described. A variety of nucleophiles could be engaged in this transformation, thus permitting the synthesis of complex sulfonamides and sulfonates. In most cases, these reactions are highly selective, simple, and clean, affording products at excellent yields.

## 1. Introduction

**Scheme 1:**
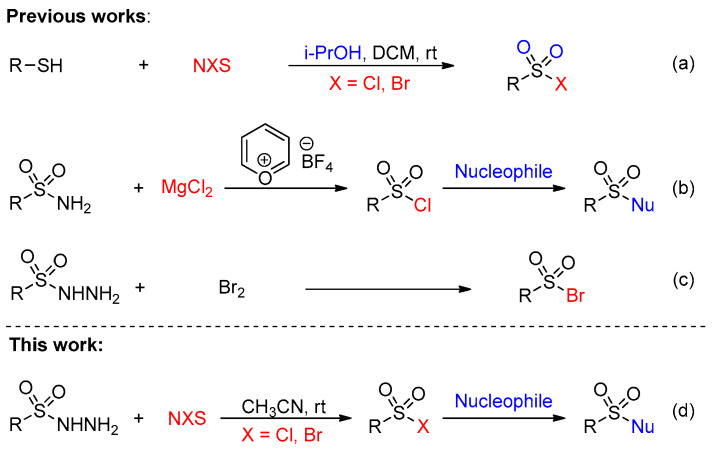
Synthesis of sulfonyl halides. (**a**) The oxidation of thiols using NCS/NBS-iPrOH; (**b**) Sulfonyl chloride formation from sulfonamides enabled by pyrylium salt; (**c**) The synthesis of sulfonyl bromides from sulfonyl hydrazides with bromine; (**d**) The reaction of sulfonyl hydrazides with NCS/NBS.

Sulfonyl chlorides are the most prevalent reagents for the installation of the sulfonyl protecting group [1], which can be converted into numerous sulfonyl derivatives [2,3,4,5,6], undergo diverse desulfitative cross-couplings [7,8], and serve as arylating agents [9,10,11,12,13]. In addition, they have been widely used as important building blocks for the manufacture of elastomers, pharmaceuticals, dyes, detergents, ion exchange resins, and herbicides [14,15,16]. Recently, they have also exhibited important applications in building synthetic receptors [17] and catalysts [18,19,20]. Given their importance in various fields, there is a strong interest in developing efficient synthetic methods for preparing them. The oxidative chlorination of thiols has been a frequently applied synthetic pathway using several combinations of oxidants and chloride sources [21,22,23,24,25,26,27,28]. In addition, chlorination with different sulfur compounds [29,30,31,32] or Grignard reactions [33] have been developed as efficient methods for the synthesis of sulfonyl chlorides. However, in the reported methods, toxic and highly corrosive reagents were required, the formation of some side products was reported, and tedious workup procedures for the isolation of the pure products were necessary. Therefore, the development of a milder and more practical method for the synthesis of sulfonyl chlorides is highly desirable. In 2017, Montelongo’s group developed an elegant strategy for the synthesis of sulfonyl chlorides and bromides by the oxidation of thiols using NCS/NBS-iPrOH as an oxyhalogenation reagent (Scheme 1a) [34]. Recently, Cornella reported highly selective conversion reactions of primary sulfonamides to the corresponding sulfonyl chlorides and fluorides using pyrylium salt as an activating reagent (Scheme 1b) [35].

Sulfonyl hydrazides are attractive targets because of their wide applications in organic synthesis, particularly in total synthesis [36]. Because of the high activity of sulfonyl chloride, they could react with hydrazine hydrate to synthesize various sulfonyl hydrazides [36]. We suspected that more stable sulfonyl hydrazides could convert to sulfonyl chloride, in which sulfonyl hydrazide can be used as a protective reagent in organic synthesis. Magnotta reported a simple strategy for the synthesis of sulfonyl bromides from sulfonyl hydrazides with bromine (Scheme 1c) [37]. This strategy represents a highly valuable synthetic tool but leaves ample opportunities to develop more green and gentle reaction systems to construct sulfonyl chlorides/bromides. Herein, we describe that the sulfonyl hydrazides react with NCS/NBS under mild reaction conditions, providing convenient and efficient access to sulfonyl chlorides/bromides (Scheme 1d). 

## 2. Results

We commenced our study by investigating 4-methylbenzenesulfonhydrazide (**1a**) and a halogen source (**2**). Inspired by the work of Cornella, we first evaluated the reaction using MgCl_2_ as the halogen source in CH_3_CN at room temperature without any catalysts or additives; however, no appreciable formation of target product **3a** was detected in the reaction mixture (Table 1, entry 1). Subsequent screening of a large panel of chlorides found that the use of CuCl resulted in the generation of **3a** at a 38% yield (Table 1, entries 2–7). We further investigated the reactivity of organic chlorides, and the results suggested that NCS (*N*-chlorosuccinimide) was optimal to provide a comparable 99% yield (Table 1, entries 8–10). Furthermore, the replacement of CH_3_CN with other solvents hampered product formation to various degrees (Table 1, entries 11−16). Furthermore, the replacement of NCS with NBS (*N*-bromosuccinimide) also smoothly provided the target product sulfonyl bromide **4a** at an 87% yield (Table 1, entry 17). However, when using NIS (*N*-iodosuccinimide) as the substrate, the corresponding product **5a** was not formed (Table 1, entry 18).

## 3. Discussion

**Scheme 2:**
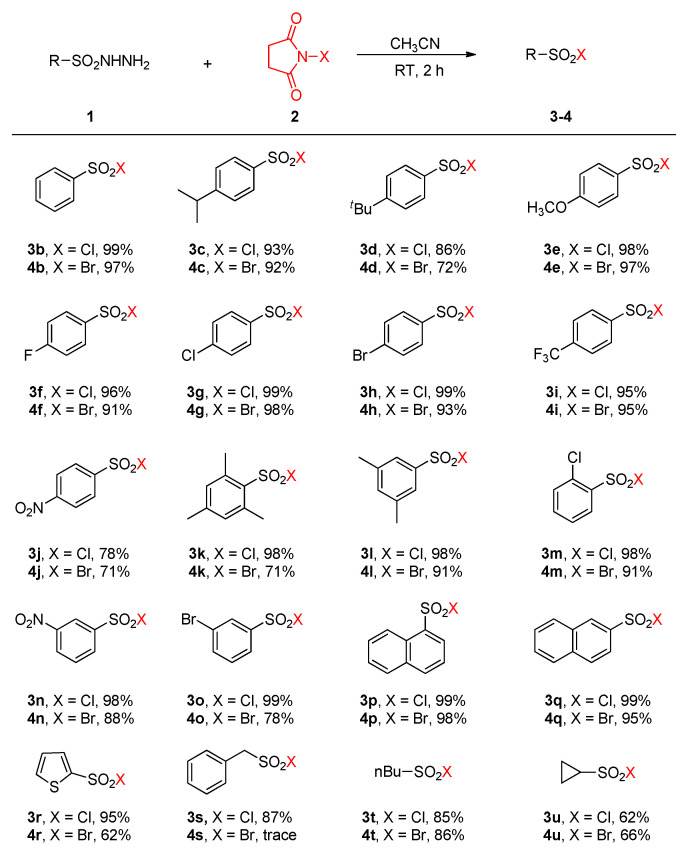
Scope of the conversion of sulfonyl hydrazides to sulfonyl chlorides and sulfonyl bromides. Conditions: **1** (0.3 mmol), **2** (0.6 mmol), and CH_3_CN (2.0 mL), rt for 2 h under air.

With the obtained optimized reaction conditions, we explored the substrate’s scope. As shown in Scheme 2, various ortho-, meta-, and parasubstituted arylsulfonyl hydrazides, including the aryl and alkyl substitution, could react smoothly with NCS to deliver the desired products in good to excellent yields (**3a**–**3u**). The substitution in the aromatic ring of sulfonyl hydrazides, regardless of the electron-donating or electron-withdrawing groups, hardly affected the reactivity of the reaction. To our delight, the naphthyl and heterocyclic sulfonyl hydrazides, such as thiophene, also afforded the corresponding products in satisfactory yields (**3p**–**3r**). In addition, both benzylsulfonyl hydrazide and alkylsulfonyl hydrazides could undergo this process smoothly to afford the corresponding products (**3s**−**3u**) in moderate to high yields. On the other hand, NBS was subjected to the reaction under the same reaction conditions. In contrast with NCS, NBS showed relatively weak reactivity, and the corresponding sulfonyl bromide products could also be obtained in moderate to good yields (**4a**–**4u**). Unfortunately, benzylsulfonyl hydrazide was not suitable for this transformation (**4s**).

**Scheme 3:**
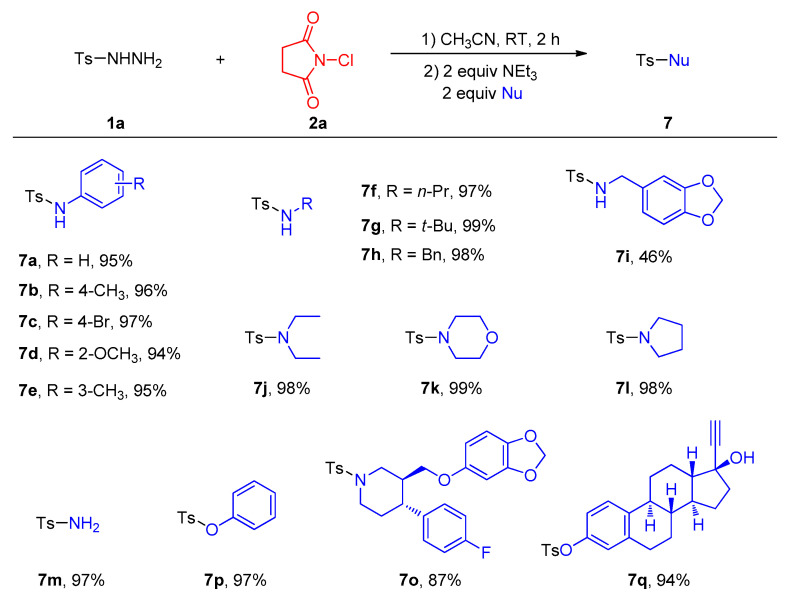
Synthesis of sulfonamides or sulfonates under the optimum conditions. Conditions: (1) **1a** (0.3 mmol), **2a** (0.6 mmol), and CH_3_CN (2.0 mL), rt for 2 h under air; (2) NEt_3_ (0.6 mmol), nucleophile (0.6 mmol), rt for 2 h under air.

Having established a protocol for synthesizing highly versatile sulfonyl chlorides and considering that the importance of complex sulfonamide and sulfonates in drug discovery, we next assessed the scope of the reaction between different nucleophiles in the presence of a base in one pot. As listed in Scheme 3, both aromatic and aliphatic primary amines reacted smoothly with **1a** and **2a** under air, giving the corresponding sulfonamides in moderate to excellent yields (**7a**–**7i**). It was discovered that secondary alkyl amines were suitable participants (**7j**–**7l**), as along with ammonia (**7m**). Phenol was able to furnish corresponding sulfonate **7p** at a good yield. In addition, we turned our attention to biologically active compounds bearing various functional groups embedded in their structure. Paroxetine was successfully applied in this transformation and afforded an 87% yield of the corresponding sulfonamides over two steps (**7o**). As we predicted, ethynyl estradiol was also compatible in sulfonate formation via a simple two-step process (**7q**).

**Scheme 4:**
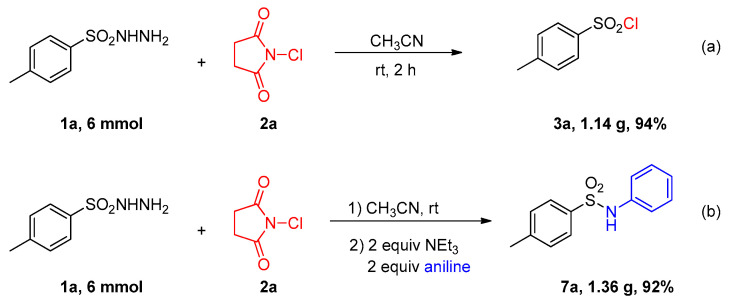
(**a**) Formation of sulfonyl chloride at a gram scale; (**b**) Formation of sulfonamide at a gram scale.

To further illustrate the robustness of the protocol, we scaled up this sulfonyl chloride synthesis. Without modification of the original protocol, 6 mmol of **1a** could successfully be converted to **3a** at a 94% yield (Scheme 4a). By adding aniline to the above reaction system without any separation, sulfonamide **7a** could be obtained at a yield of 92% (Scheme 4b).

**Scheme 5:**
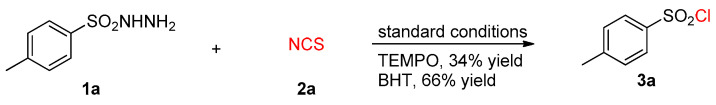
Preliminary research mechanism.

To gain insight into the reaction mechanism, several control experiments were designed to understand the mechanism of this process. We performed experiments with the addition of the radical inhibitor TEMPO (2,2,6,6-tetramethyl-1-piperidinyloxy) or BHT (butylated hydroxytoluene), and the product yield was significantly reduced in both cases (Scheme 5).

**Scheme 6:**
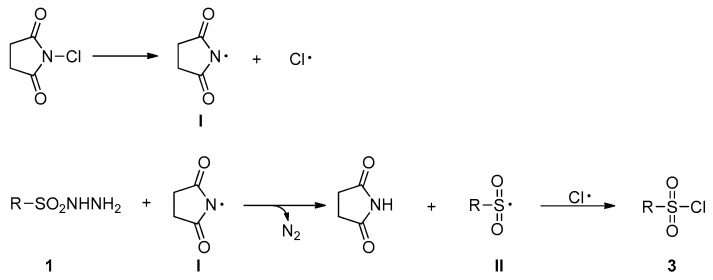
Proposed reaction mechanism.

On the basis of the above results and literature, a plausible mechanism was proposed, as shown in Scheme 6. Initially, nitrogen center radical **I** and chlorine radical were generated from NCS [38,39,40]. Then, sulfonyl hydrazide could transfer to sulfonyl radical **II** with the release of nitrogen gas under oxidative conditions [38,39,40]. Finally, sulfonyl radical **II** went through a coupling reaction with chlorine radical to afford the final product **3**. 

## 4. Materials and Methods

### 4.1. General Information

NMR data were obtained for ^1^H at 400 MHz and for ^13^C at 100 MHz. Chemical shifts were reported in ppm from tetramethylsilane with the solvent resonance as the internal standard in CDCl_3_ solution. Column chromatography was performed on silica gel (300–400 mesh) eluting with ethyl acetate/petroleum ether. TLC was performed on glass-backed silica plates. UV light and I_2_ were used to visualize products. All chemicals were used without purification as commercially available unless otherwise noted. 

### 4.2. General Procedure for Synthesis of Sulfonyl Chloride ***3*** or Sulfonyl Bromide ***4***

*N*-Chlorosuccinimide **2a** or *N*-bromosuccinimide **2b** (0.6 mmol, 2.0 equiv) was added to a solution of sulfonyl hydrazide **1** (0.3 mmol) in CH_3_CN (2 mL) in one portion. The mixture was stirred at room temperature for 2 h. The solvent was removed, and the residue was purified by flash column chromatography (petroleum ether/ethyl acetate) to provide the corresponding sulfonyl chloride **3** or sulfonyl bromide **4**.

### 4.3. Large-Scale Reaction for the Synthesis of Sulfonyl Chloride ***3a***

*N*-Chlorosuccinimide **2a** (12 mmol, 2.0 equiv, 1.6 g) was added to a solution of 4-methylbenzenesulfonhydrazide **1a** (6 mmol, 1.12 g) in CH_3_CN (10 mL) in one portion. The mixture was stirred at room temperature for 2 h. The solvent was removed, and the residue was purified by flash column chromatography (PE/EA = 20:1) to provide the corresponding p-toluenesulfonyl chloride **3a** (white solid, 1.14 g, 94%).

### 4.4. General Procedure for One-Port Reaction with Nucleophile

*N*-Chlorosuccinimide **2a** (0.6 mmol, 2.0 equiv) was added to a solution of 4-methylbenzenesulfonhydrazide **1a** (0.3 mmol) in CH_3_CN (2 mL) in one portion. The mixture was stirred at room temperature for 2 h. Then, Et_3_N (0.6 mmol, 2.0 equiv) and nucleophile (0.6 mmol, 2.0 equiv) were added to the above reaction system, and the mixture was stirred at room temperature for 2 h. The solvent was removed, and the residue was purified by flash column chromatography (PE/EA) to provide the corresponding sulfonamides and sulfonate **7**.

### 4.5. Large-Scale Reaction for the Synthesis of ***7a***

*N*-Chlorosuccinimide **2a** (12 mmol, 2.0 equiv) was added to a solution of 4-methylbenzenesulfonhydrazide **1a** (6 mmol, 1.12 g) in CH_3_CN (10 mL) in one portion. The mixture was stirred at room temperature for 2 h. Then, Et_3_N (12 mmol, 2.0 equiv) and aniline (12 mmol, 2.0 equiv) were added to the above reaction system, and the mixture was stirred at room temperature for 2 h. The solvent was removed, and the residue was purified by flash column chromatography (PE/EA) to provide the corresponding sulfonamide **7a** (brown solid, 1.39 g, 94%).

## 5. Conclusions

In conclusion, we successfully developed an efficient, simple, practical approach for the construction of sulfonyl chlorides/bromides from sulfonyl hydrazide. This methodology allows a wide substrate scope, utilizes readily available starting materials, and provides operational simplicity. Efforts to develop more direct applications in the chemical community are in progress in our laboratory.

## Figures and Tables

**Table 1:**
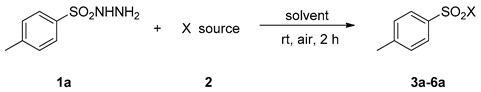
Optimization of reaction conditions ^a^.

Entry	X Source	Solvent	Product	Yield (%) ^b^
1	MgCl_2_	CH_3_CN	**3a**	N.R.
2	NaCl	CH_3_CN	**3a**	N.R.
3	CaCl_2_	CH_3_CN	**3a**	N.R.
4	ZnCl_2_	CH_3_CN	**3a**	N.R.
5	HCl	CH_3_CN	**3a**	N.R.
6	CuCl	CH_3_CN	**3a**	38
7	FeCl_3_	CH_3_CN	**3a**	8
8	SOCl_2_	CH_3_CN	**3a**	trace
9	PCl_5_	CH_3_CN	**3a**	65
10	NCS	CH_3_CN	**3a**	99
11	NCS	CH_2_Cl_2_	**3a**	91
12	NCS	EtOAc	**3a**	94
13	NCS	DME	**3a**	87
14	NCS	THF	**3a**	56
15	NCS	DCE	**3a**	58
16	NCS	Dioxane	**3a**	75
17	NBS	CH_3_CN	**4a**	87
18	NIS	CH_3_CN	**5a**	N.R.

^a^ Unless noted otherwise, reactions were performed with **1a** (0.3 mmol) and **X source** (0.6 mmol, 2 equiv) in 2 mL solvent and room temperature under open air for 2 h. ^b^ Isolated yield. N.R.: no reaction, NCS: *N*-chlorosuccinimide, NBS: *N*-bromosuccinimide, NIS: *N*-iodosuccinimide.

## Data Availability

Data are contained within the article and the supplementary material.

## Supplementary Materials

The following are available online, Characterization of products and ^1^H and ^13^C-NMR spectra are available online (see manuscript-supplementary).

## Sample Availability

Samples of the compounds are available from the authors.
