# Supplementary material for: Facile Synthesis of Sulfonyl Chlorides/Bromides from Sulfonyl Hydrazides
Source: Molecules. 2021 Sep 13;26(18):5551. doi: 10.3390/molecules26185551 (PMC8471771; doi:10.3390/molecules26185551)
Supplement: Supplementary file 1 [file molecules-26-05551-s001.zip › molecules-1360676-supplementary.pdf]

1. Characterization of products

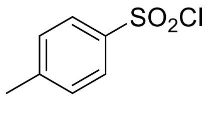

3a

4-Methylbenzene-1-sulfonyl chloride (**3a**). white solid, ^{1}H NMR (400 MHz, $CDCl_3$) δ 7.92 (d, *J* = 8.2 Hz, 2H), 7.41 (d, *J* = 8.1 Hz, 2H), 2.49 (s, 3H). ^{13}C NMR (101 MHz, $CDCl_3$) δ 146.9, 141.7, 130.3, 127.1, 21.8.

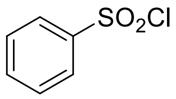

3b

Benzenesulfonyl chloride (**3b**). colorless liquid, ^{1}H NMR (400 MHz, $CDCl_3$) δ 8.05 (d, *J* = 8.1 Hz, 2H), 7.76 (t, *J* = 7.4 Hz, 1H), 7.64 (t, *J* = 7.8 Hz, 2H). ^{13}C NMR (101 MHz, $CDCl_3$) δ 144.3, 135.3, 129.7, 126.9.

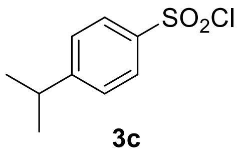

3c

4-Isopropylbenzene-1-sulfonyl chloride (**3c**). white solid, ^{1}H NMR (400 MHz, $CDCl_3$) δ 7.96 (d, *J* = 8.3 Hz, 2H), 7.47 (d, *J* = 8.3 Hz, 2H), 3.04 (hept, *J* = 6.9 Hz, 1H), 1.30 (d, *J* = 7.0 Hz, 6H). ^{13}C NMR (101 MHz, $CDCl_3$) δ 157.4, 141.8, 127.7, 127.2, 34.4, 23.5.

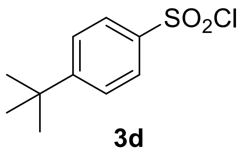

3d

4-(*tert*-Butyl)benzene-1-sulfonyl chloride (**3d**). white solid, ^{1}H NMR (400 MHz, $CDCl_3$) δ 7.96 (d, *J* = 8.3 Hz, 2H), 7.62 (d, *J* = 8.3 Hz, 2H), 1.37 (s, 9H). ^{13}C NMR (101 MHz, $CDCl_3$) δ 159.6, 141.5, 126.9, 126.7, 35.5, 30.9.

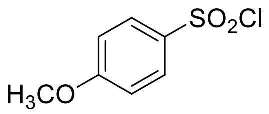

3e

4-Methoxybenzene-1-sulfonyl chloride (**3e**). light yellow liquid, ^{1}H NMR (400 MHz, $CDCl_3$) δ 7.89 (d, *J* = 8.9 Hz, 2H), 6.97 (d, *J* = 8.9 Hz, 2H), 3.85 (s, 3H). ^{13}C NMR (101 MHz, $CDCl_3$) δ 164.9, 136.0, 129.5, 114.7, 56.0.

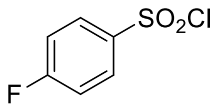

3f

4-Fluorobenzene-1-sulfonyl chloride (**3f**). light yellow solid, ^{1}H NMR (400 MHz, $CDCl_3$) δ 8.14 – 8.03 (m, 2H), 7.31 (t, *J* = 8.4 Hz, 2H). ^{19}F NMR (376 MHz, $CDCl_3$) δ -99.56. ^{13}C NMR (101 MHz, $CDCl_3$) δ 166.4 (d, *J* = 260.6 Hz), 140.3 (d, *J* = 3.0 Hz), 130.1(d, *J* = 10.1 Hz), 117.1 (d, *J* = 23.2 Hz).

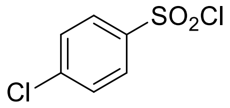

3g

4-Chlorobenzene-1-sulfonyl chloride (**3g**). white solid, ^{1}H NMR (400 MHz, $CDCl_3$) δ 7.99 (d, *J* = 8.2 Hz, 2H), 7.61 (d, *J* = 8.2 Hz, 2H). ^{13}C NMR (101 MHz, $CDCl_3$) δ 142.6, 142.2, 130.0, 128.4.

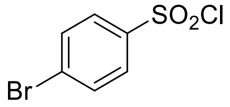

3h

4-Bromobenzene-1-sulfonyl chloride (**3h**). white solid, ^{1}H NMR (400 MHz, $CDCl_3$) δ 7.91 (d, *J* = 8.4 Hz, 2H), 7.78 (d, *J* = 8.4 Hz, 2H). ^{13}C NMR (101 MHz, $CDCl_3$) δ 143.1, 133.0, 130.8, 128.4.

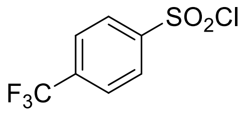

3i

4-(Trifluoromethyl)benzene-1-sulfonyl chloride (**3i**). yellow solid, ^{1}H NMR (400 MHz, $CDCl_3$) δ 8.20 (d, *J* = 8.3 Hz, 2H), 7.92 (d, *J* = 8.3 Hz, 2H). ^{19}F NMR (376 MHz, $CDCl_3$) δ -63.42. ^{13}C NMR (101 MHz, $CDCl_3$) δ 147.1, 136.7 (d, *J* = 33.3 Hz), 127.7, 127.0 (q, *J* = 3.7 Hz), 124.1, 121.4 (d, *J* = 273.7 Hz).

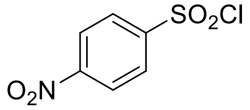

3j

4-Nitrobenzene-1-sulfonyl chloride (**3j**). colorless liquid, ^{1}H NMR (400 MHz, $CDCl_3$) δ 8.49 (d, *J* = 8.7 Hz, 2H), 8.27 (d, *J* = 8.7 Hz, 2H). ^{13}C NMR (101 MHz, $CDCl_3$) δ 151.3, 148.6, 128.5, 125.0.

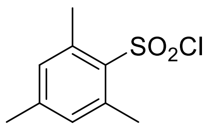

3k

2,4,6-Trimethylbenzene-1-sulfonyl chloride (**3k**). light yellow liquid, ^{1}H NMR (400 MHz, $CDCl_3$) δ 6.94 (s, 2H), 2.63 (s, 6H), 2.26 (s, 3H). ^{13}C NMR (101 MHz, $CDCl_3$) δ 145.4, 139.9, 139.4, 132.3, 22.8,

21.1.

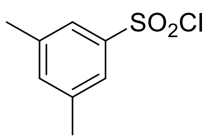

3l

3,5-Dimethylbenzene-1-sulfonyl chloride (**3l**). white solid, ^{1}H NMR (400 MHz, $CDCl_3$) δ 7.65 (s, 2H), 7.35 (s, 1H), 2.44 (s, 6H). ^{13}C NMR (101 MHz, $CDCl_3$) δ 144.1, 140.0, 136.9, 124.4, 21.2.

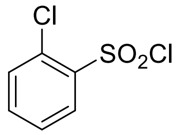

3m

2-Chlorobenzene-1-sulfonyl chloride (**3m**). colorless liquid, ^{1}H NMR (400 MHz, $CDCl_3$) δ 8.16 (d, J = 8.1 Hz, 1H), 7.72 – 7.61 (m, 2H), 7.52 (t, J = 7.3 Hz, 1H). ^{13}C NMR (101 MHz, $CDCl_3$) δ 141.2, 136.0, 132.9, 132.8, 130.4, 127.4.

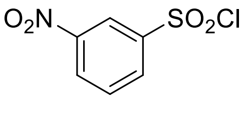

3n

3-Nitrobenzene-1-sulfonyl chloride (**3n**). light yellow liquid, ^{1}H NMR (400 MHz, $CDCl_3$) δ 8.18 (s, 1H), 7.99 (d, J = 8.0 Hz, 1H), 7.88 (d, J = 8.0 Hz, 1H), 7.52 (t, J = 8.0 Hz, 1H). ^{13}C NMR (101 MHz, $CDCl_3$) δ 145.5, 138.3, 131.1, 129.8, 125.5, 123.4.

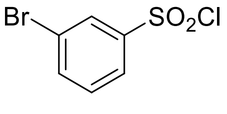

3o

3-Bromobenzene-1-sulfonyl chloride (**3o**). colorless liquid, ^{1}H NMR (400 MHz, $CDCl_3$) δ 8.80 (s, 1H), 8.55 (d, J = 8.2 Hz, 1H), 8.32 (d, J = 7.9 Hz, 1H), 7.85 (t, J = 8.1 Hz, 1H). ^{13}C NMR (101 MHz, $CDCl_3$) δ 148.3, 145.4, 132.3, 131.4, 129.6, 122.3.

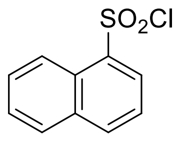

3p

Naphthalene-1-sulfonyl chloride (**3p**). white solid, ^{1}H NMR (400 MHz, $CDCl_3$) δ 8.79 (d, J = 8.7 Hz, 1H), 8.37 (d, J = 7.5 Hz, 1H), 8.21 (d, J = 8.2 Hz, 1H), 8.00 (d, J = 8.2 Hz, 1H), 7.80 (t, J = 7.8 Hz, 1H), 7.69 (t, J = 7.6 Hz, 1H), 7.59 (t, J = 7.9 Hz, 1H). ^{13}C NMR (101 MHz, $CDCl_3$) δ 139.5, 137.0, 134.4, 129.4, 129.3, 129.2, 127.7, 127.4, 124.1, 123.9

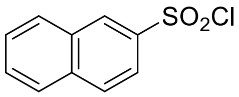

3q

Naphthalene-2-sulfonyl chloride (**3q**). white solid, ^{1}H NMR (400 MHz, $CDCl_3$) δ 8.58 (s, 1H), 8.00 (dt, *J* = 20.3, 9.0 Hz, 4H), 7.74 (t, *J* = 7.5 Hz, 1H), 7.68 (t, *J* = 7.5 Hz, 1H). ^{13}C NMR (101 MHz, $CDCl_3$) δ 141.0, 135.7, 131.6, 130.3, 130.2, 129.8, 128.9, 128.3, 128.1, 121.2.

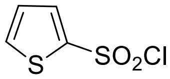

3r

Thiophene-2-sulfonyl chloride (**3r**). light yellow liquid, ^{1}H NMR (400 MHz, $CDCl_3$) δ 7.92 – 7.87 (m, 1H), 7.84 (d, *J* = 5.0 Hz, 1H), 7.19 (t, *J* = 4.5 Hz, 1H). ^{13}C NMR (101 MHz, $CDCl_3$) δ 144.0, 135.8, 135.8, 134.9, 127.7.

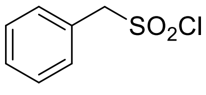

3s

Phenylmethanesulfonyl chloride (**3s**). white solid, ^{1}H NMR (400 MHz, $CDCl_3$) δ 7.46 (d, *J* = 8.6 Hz, 5H), 4.86 (s, 2H). ^{13}C NMR (101 MHz, $CDCl_3$) δ 131.4, 130.2, 129.2, 126.1, 70.9.

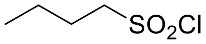

3t

Butane-1-sulfonyl chloride (**3t**). colorless liquid, ^{1}H NMR (400 MHz, $CDCl_3$) δ 3.75 – 3.61 (m, 2H), 2.10 – 1.96 (m, 2H), 1.62 – 1.46 (m, 2H), 1.01 (t, *J* = 7.4 Hz, 3H). ^{13}C NMR (101 MHz, $CDCl_3$) δ 65.2, 26.1, 20.8, 13.3.

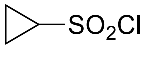

3u

Cyclopropanesulfonyl chloride (**3u**). light yellow liquid, ^{1}H NMR (400 MHz, $CDCl_3$) δ 3.35 – 3.23 (m, 1H), 1.64 – 1.55 (m, 2H), 1.37 (q, *J* = 7.2 Hz, 2H). ^{13}C NMR (101 MHz, $CDCl_3$) δ 43.1, 9.1.

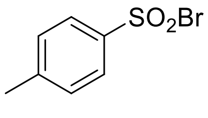

4a

4-Methylbenzene-1-sulfonyl bromide (**4a**). white solid, ^{1}H NMR (400 MHz, $CDCl_3$) δ 7.93 (d, *J* = 8.2 Hz, 2H), 7.41 (d, *J* = 8.2 Hz, 2H), 2.49 (s, 3H). ^{13}C NMR (101 MHz, $CDCl_3$) δ 146.8, 141.7, 130.2, 127.0, 21.8.

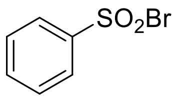

4b

Benzenesulfonyl bromide (**4b**). light yellow liquid, ^{1}H NMR (400 MHz, $CDCl_3$) δ 7.93 (d, *J* = 8.1 Hz, 2H), 7.67 (t, *J* = 7.4 Hz, 1H), 7.54 (t, *J* = 7.7 Hz, 2H). ^{13}C NMR (101 MHz, $CDCl_3$) δ 147.1, 135.2, 129.6, 126.4.

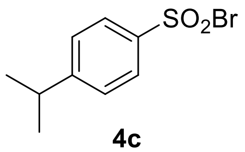

4c

4-Isopropylbenzene-1-sulfonyl bromide (**4c**). yellow liquid, ^{1}H NMR (400 MHz, $CDCl_3$) δ 7.91 (d, *J* = 8.4 Hz, 2H), 7.45 (d, *J* = 8.3 Hz, 2H), 3.04 (dt, *J* = 13.8, 6.9 Hz, 1H), 1.30 (d, *J* = 7.0 Hz, 6H). ^{13}C NMR (101 MHz, $CDCl_3$) δ 157.3, 144.7, 127.6, 126.6, 34.4, 23.5.

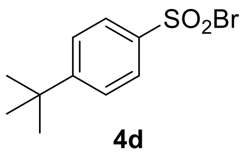

4d

4-(*tert*-Butyl)benzene-1-sulfonyl bromide (**4d)**. yellow solid, ^{1}H NMR (400 MHz, $CDCl_3$) δ 7.92 (d, *J* = 8.6 Hz, 2H), 7.61 (d, *J* = 8.6 Hz, 2H), 1.37 (s, 9H). ^{13}C NMR (101 MHz, $CDCl_3$) δ 159.6, 144.4, 126.6, 126.4, 35.6, 30.9.

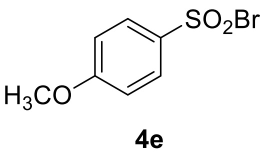

4e

4-Methoxybenzene-1-sulfonyl bromide (**4e**). light yellow liquid, ^{1}H NMR (400 MHz, $CDCl_3$) δ 7.86 (d, *J* = 8.9 Hz, 2H), 6.96 (d, *J* = 8.9 Hz, 2H), 3.85 (s, 3H). ^{113}C NMR (101 MHz, $CDCl_3$) δ 164.8, 139.0, 129.0, 114.53, 56.0.

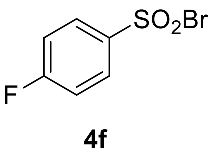

4f

4-Fluorobenzene-1-sulfonyl bromide (**4f**). light yellow liquid, ^{1}H NMR (400 MHz, $CDCl_3$) δ 8.05 (dd, *J* = 8.8, 4.8 Hz, 2H), 7.29 (t, *J* = 8.4 Hz, 2H). ^{19}F NMR (376 MHz, $CDCl_3$) δ -99.54. ^{13}C NMR (101 MHz, $CDCl_3$) δ 166.2 (d, *J* = 261.6 Hz), 143.0 (d, *J* = 3.0Hz), 129.6 (d, *J* = 10.1 Hz), 116.9 (d, *J* = 23.2 Hz).

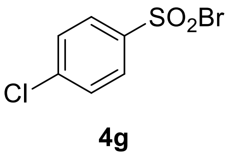

4g

4-Chlorobenzene-1-sulfonyl bromide (**4g**). light yellow solid, ^{1}H NMR (400 MHz, $CDCl_3$) δ 7.95 (d,

J = 8.4 Hz, 2H), 7.59 (d, J = 8.4 Hz, 2H). ^{13}C NMR (101 MHz, $CDCl_3$) δ 145.3, 142.0, 129.9, 127.9.

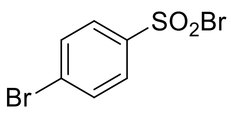

4h

4-Bromobenzene-1-sulfonyl bromide (**4h**). yellow solid, ^{1}H NMR (400 MHz, $CDCl_3$) δ 7.87 (d, J = 8.6 Hz, 2H), 7.76 (d, J = 8.6 Hz, 2H). ^{13}C NMR (101 MHz, $CDCl_3$) δ 145.8, 132.9, 130.7, 127.8.

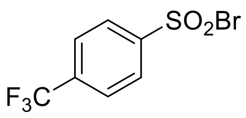

4i

4-(Trifluoromethyl)benzene-1-sulfonyl bromide (**4i**). colorless liquid, ^{1}H NMR (400 MHz, $CDCl_3$) δ 8.15 (d, J = 8.3 Hz, 2H), 7.90 (d, J = 8.3 Hz, 2H). ^{19}F NMR (376 MHz, $CDCl_3$) δ -63.36. ^{13}C NMR (101 MHz, $CDCl_3$) δ 149.6, 136.5 (d, J = 34.3 Hz), 127.1, 126.9 (q, J = 3.7 Hz), 124.1, 121.4.

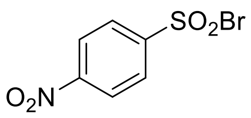

4j

4-Nitrobenzene-1-sulfonyl bromide (**4j**). colorless liquid, ^{1}H NMR (400 MHz, $CDCl_3$) δ 8.48 (d, J = 8.6 Hz, 2H), 8.22 (d, J = 8.6 Hz, 2H). ^{13}C NMR (101 MHz, $CDCl_3$) δ 151.1, 150.8, 127.2, 124.9.

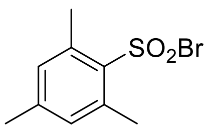

4k

2,4,6-Trimethylbenzene-1-sulfonyl bromide (**4k**). yellow solid, ^{1}H NMR (400 MHz, $CDCl_3$) δ 7.01 (s, 2H), 2.72 (s, 6H), 2.35 (s, 3H). ^{13}C NMR (101 MHz, $CDCl_3$) δ 145.5, 142.7, 139.0, 132.3, 22.9, 21.2.

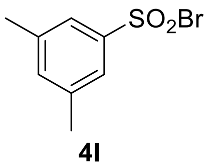

4l

3,5-Dimethylbenzene-1-sulfonyl bromide (**4l**). light yellow solid, ^{1}H NMR (400 MHz, $CDCl_3$) δ 7.60 (s, 2H), 7.34 (s, 1H), 2.44 (s, 6H). ^{13}C NMR (101 MHz, $CDCl_3$) δ 146.99, 139.9, 136.8, 123.8, 21.2.

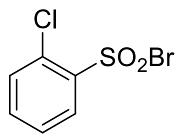

4m

2-Chlorobenzene-1-sulfonyl bromide (**4m**). colorless liquid, ^{1}H NMR (400 MHz, $CDCl_3$) δ 8.10 (d, J =

8.1 Hz, 1H), 7.71 – 7.59 (m, 2H), 7.50 (t, J = 7.5 Hz, 1H). ^{13}C NMR (101 MHz, $CDCl_3$) δ 143.2, 135.9, 133.0, 132.7, 129.8, 127.3.

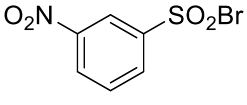

4n

3-Nitrobenzene-1-sulfonyl bromide (**4n**). light yellow liquid, ^{1}H NMR (400 MHz, $CDCl_3$) δ 8.13 (s, 1H), 7.94 (d, J = 8.0 Hz, 1H), 7.86 (d, J = 8.0 Hz, 1H), 7.51 (t, J = 8.0 Hz, 1H). ^{13}C NMR (101 MHz, $CDCl_3$) δ 148.0, 138.2, 131.0, 129.2, 124.9, 123.2.

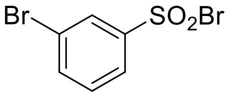

4o

3-Bromobenzene-1-sulfonyl bromide (**4o**). light yellow liquid, ^{1}H NMR (400 MHz, $CDCl_3$) δ 8.83 (s, 1H), 8.61 (d, J = 8.1 Hz, 1H), 8.35 (d, J = 7.9 Hz, 1H), 7.91 (t, J = 8.1 Hz, 1H). ^{13}C NMR (101 MHz, $CDCl_3$) δ 148.1, 147.9, 131.7, 131.2, 129.4, 121.8.

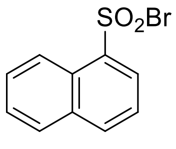

4p

Naphthalene-1-sulfonyl bromide (**4p**). light yellow solid, ^{1}H NMR (400 MHz, $CDCl_3$) δ 8.87 (d, J = 8.7 Hz, 1H), 8.32 (d, J = 7.5 Hz, 1H), 8.21 (d, J = 8.2 Hz, 1H), 8.01 (d, J = 8.2 Hz, 1H), 7.82 (t, J = 7.8 Hz, 1H), 7.69 (t, J = 7.6 Hz, 1H), 7.56 (t, J = 7.9 Hz, 1H). ^{13}C NMR (101 MHz, $CDCl_3$) δ 142.0, 136.9, 134.4, 129.3, 129.2, 128.5, 127.8, 127.1, 123.8, 123.8.

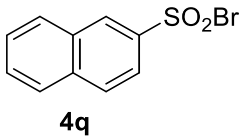

4q

Naphthalene-2-sulfonyl bromide (**4q**). white solid, ^{1}H NMR (400 MHz, $CDCl_3$) δ 8.53 (s, 1H), 8.02 (t, J = 6.9 Hz, 2H), 7.95 (d, J = 8.5 Hz, 2H), 7.74 (t, J = 7.5 Hz, 1H), 7.67 (t, J = 7.5 Hz, 1H). ^{13}C NMR (101 MHz, $CDCl_3$) δ 143.8, 135.7, 131.4, 130.3, 130.1, 129.9, 128.4, 128.1, 120.9.

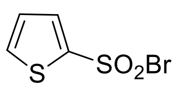

4r

Thiophene-2-sulfonyl bromide (**4r**). light yellow liquid, ^{1}H NMR (400 MHz, $CDCl_3$) δ 7.87 (d, J = 3.8 Hz, 1H), 7.81 (d, J = 5.0 Hz, 1H), 7.18 (t, J = 4.5 Hz, 1H). ^{13}C NMR (101 MHz, $CDCl_3$) δ 147.0, 135.4,

134.1, 127.5.

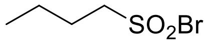

4t

Butane-1-sulfonyl bromide (**4t**). light yellow liquid, ^{1}H NMR (400 MHz, $CDCl_3$) δ 3.82 – 3.69 (m, 2H), 2.08 – 1.94 (m, 2H), 1.64 – 1.46 (m, 2H), 1.01 (t, J = 7.4 Hz, 3H). ^{13}C NMR (101 MHz, $CDCl_3$) δ 69.4, 26.5, 20.6, 13.4.

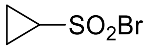

4u

Cyclopropanesulfonyl bromide (**4u**). light yellow liquid, ^{1}H NMR (400 MHz, $CDCl_3$) δ 3.49 – 3.41 (m, 1H), 1.63 – 1.57 (m, 2H), 1.39 – 1.33 (m, 2H). ^{13}C NMR (101 MHz, $CDCl_3$) δ 47.5, 10.0.

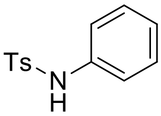

7a

4-Methyl-N-phenylbenzenesulfonamide (**7a**). brown solid, ^{1}H NMR (400 MHz, $CDCl_3$) δ 7.70 (d, J = 8.1 Hz, 2H), 7.47 (s, 1H), 7.20 (t, J = 7.8 Hz, 4H), 7.13 – 7.03 (m, 3H), 2.34 (s, 3H). ^{13}C NMR (101 MHz, $CDCl_3$) δ 143.8, 136.6, 135.9, 129.6, 129.2, 127.2, 125.0, 121.3, 21.4.

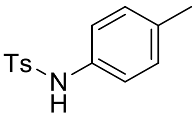

7b

4-Methyl-N-(p-tolyl)benzenesulfonamide (**7b**). white solid, ^{1}H NMR (400 MHz, CDCl3) δ 7.67 (d, J = 8.0 Hz, 2H), 7.34 (d, J = 5.9 Hz, 1H), 7.18 (d, J = 7.9 Hz, 2H), 6.99 (s, 4H), 2.33 (s, 3H), 2.23 (s, 3H). ^{13}C NMR (101 MHz, $CDCl_3$) δ 143.6, 136.0, 135.0, 133.8, 129.7, 129.5, 127.2, 122.0, 21.4, 20.7.

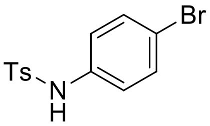

7c

N-(4-bromophenyl)-4-methylbenzenesulfonamide (**7c**). white solid, ^{1}H NMR (400 MHz, $CDCl_3$) δ 7.73 (d, J = 40.3 Hz, 2H), 7.27 (d, J = 32.7 Hz, 4H), 6.99 (s, 2H), 2.37 (s, 3H). ^{13}C NMR (101 MHz, $CDCl_3$) δ 144.2, 135.7, 135.6, 132.3, 129.8, 127.2, 122.9, 118.4, 21.5.

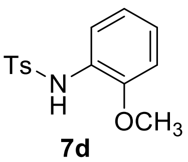

7d

N-(2-methoxyphenyl)-4-methylbenzenesulfonamide (**7d**). brown solid, ^{1}H NMR (400 MHz, $CDCl_3$) δ 7.63 (d, *J* = 8.2 Hz, 2H), 7.51 (d, *J* = 7.9 Hz, 1H), 7.17 (d, *J* = 8.0 Hz, 2H), 7.02 (dd, *J* = 10.9, 4.5 Hz, 2H), 6.87 (t, *J* = 7.7 Hz, 1H), 6.72 (d, *J* = 8.1 Hz, 1H), 3.62 (s, 3H), 2.33 (s, 3H). ^{13}C NMR (101 MHz, $CDCl_3$) δ 149.4, 143.6, 136.3, 129.3, 127.2, 126.02, 125.99, 125.2, 121.0, 110.6, 55.6, 21.4.

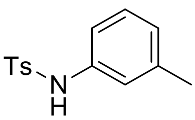

7e

4-Methyl-N-(m-tolyl)benzenesulfonamide (**7e**). white solid, ^{1}H NMR (400 MHz, $CDCl_3$) δ 7.71 (d, *J* = 8.1 Hz, 2H), 7.44 (s, 1H), 7.19 (d, *J* = 8.0 Hz, 2H), 7.07 (t, *J* = 7.7 Hz, 1H), 6.89 (dd, *J* = 15.5, 9.2 Hz, 3H), 2.34 (s, 3H), 2.23 (s, 3H). ^{13}C NMR (101 MHz, $CDCl_3$) δ 143.7, 139.2, 136.5, 136.0, 129.6, 128.9, 127.2, 125.8, 121.8, 118.0, 21.4, 21.2.

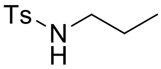

7f

4-Methyl-N-propylbenzenesulfonamide (**7f**). colorless liquid, ^{1}H NMR (400 MHz, $CDCl_3$) δ 7.77 (d, *J* = 8.1 Hz, 2H), 7.29 (t, *J* = 7.1 Hz, 2H), 4.99 (s, 1H), 2.89 (q, *J* = 6.8 Hz, 2H), 2.42 (s, 3H), 1.54 – 1.40 (m, 2H), 0.85 (t, *J* = 7.4 Hz, 3H). ^{13}C NMR (101 MHz, $CDCl_3$) δ 143.2, 137.0, 129.6 127.0, 44.9, 22.8, 21.4, 11.0.

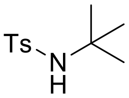

7g

N-(*tert*-Butyl)-4-methylbenzenesulfonamide (**7g**). white solid, ^{1}H NMR (400 MHz, $CDCl_3$) δ 7.80 (d, *J* = 8.1 Hz, 2H), 7.27 (d, *J* = 8.0 Hz, 2H), 5.15 (s, 1H), 2.41 (s, 3H), 1.21 (s, 9H). ^{13}C NMR (101 MHz, $CDCl_3$) δ 142.7, 140.6, 129.4, 126.9, 54.4, 30.0, 21.4.

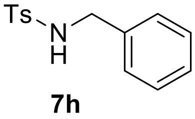

7h

N-benzyl-4-methylbenzenesulfonamide (**7h**). white solid, ^{1}H NMR (400 MHz, $CDCl_3$) δ 7.73 (d, *J* = 8.0 Hz, 2H), 7.30 – 7.21 (m, 5H), 7.18 (d, *J* = 7.5 Hz, 2H), 5.01 (t, *J* = 5.9 Hz, 1H), 4.09 (d, *J* = 6.2 Hz, 2H), 2.41 (s, 3H). ^{13}C NMR (101 MHz, $CDCl_3$) δ 143.4, 136.8, 136.3, 129.6, 128.6, 127.8, 127.7, 127.1,

47.1, 21.4.

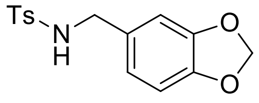

7i

N-(benzo[d][1,3]dioxol-5-ylmethyl)-4-methylbenzenesulfonamide (**7i**). white solid, ^{1}H NMR (400 MHz, $CDCl_3$) δ 7.74 (d, *J* = 7.9 Hz, 2H), 7.28 (t, *J* = 8.3 Hz, 2H), 6.65 (q, *J* = 8.2 Hz, 3H), 5.90 (s, 2H), 4.87 (t, *J* = 5.8 Hz, 1H), 4.00 (d, *J* = 6.1 Hz, 2H), 2.43 (s, 3H). ^{13}C NMR (101 MHz, $CDCl_3$) δ 147.8, 147.2, 143.4, 136.9, 130.0, 129.7, 127.1, 121.3, 108.4, 108.2, 101.1, 47.1, 21.5.

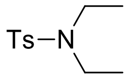

7j

N,N-diethyl-4-methylbenzenesulfonamide (**7j**). colorless liquid, ^{1}H NMR (400 MHz, $CDCl_3$) δ 7.69 (d, *J* = 8.0 Hz, 2H), 7.28 (d, *J* = 8.0 Hz, 2H), 3.22 (q, *J* = 7.1 Hz, 4H), 2.41 (s, 3H), 1.12 (t, *J* = 7.2 Hz, 6H). ^{13}C NMR (101 MHz, $CDCl_3$) δ 142.8, 137.3, 129.5, 126.9, 41.9, 21.3, 14.0.

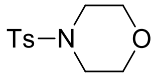

7k

4-Tosylmorpholine (**7k**). white solid, ^{1}H NMR (400 MHz, $CDCl_3$) δ 7.64 (d, *J* = 8.0 Hz, 2H), 7.35 (d, *J* = 7.9 Hz, 2H), 3.77 – 3.68 (m, 4H), 3.04 – 2.90 (m, 4H), 2.44 (s, 3H). ^{13}C NMR (101 MHz, $CDCl_3$) δ 143.9, 132.0, 129.7, 127.8, 66.0, 45.9, 21.4.

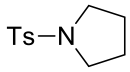

7l

1-Tosylpyrrolidine (**7l**). white solid, ^{1}H NMR (400 MHz, $CDCl_3$) δ 7.71 (d, *J* = 8.0 Hz, 2H), 7.32 (d, *J* = 7.9 Hz, 2H), 3.23 (t, *J* = 6.3 Hz, 4H), 2.43 (s, 3H), 1.74 (t, *J* = 6.4 Hz, 4H). ^{13}C NMR (101 MHz, $CDCl_3$) δ 143.2, 133.8, 129.5, 127.4, 47.8, 25.1, 21.4.

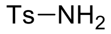

7m

4-Methylbenzenesulfonamide (**7m**). white solid, ^{1}H NMR (400 MHz, $CDCl_3$) δ 7.81 (d, *J* = 7.8 Hz, 2H), 7.31 (d, *J* = 7.9 Hz, 2H), 4.94 (s, 2H), 2.43 (s, 3H). ^{13}C NMR (101 MHz, $CDCl_3$) δ 143.6, 139.0, 129.7, 126.4, 21.5.

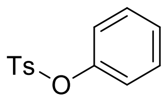

7n

Phenyl 4-methylbenzenesulfonate (**7n**). white solid, ^{1}H NMR (400 MHz, $CDCl_3$) δ 7.69 (d, *J* = 8.1 Hz, 2H), 7.33 – 7.18 (m, 5H), 6.97 (d, *J* = 7.8 Hz, 2H), 2.42 (s, 3H). ^{13}C NMR (101 MHz, $CDCl_3$) δ 149.6, 145.3, 132.3, 129.7, 129.5, 128.4, 126.7, 122.3, 21.6.

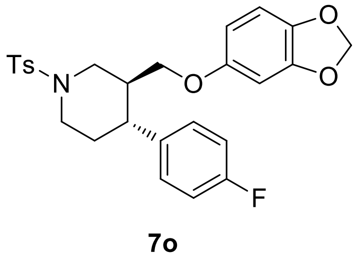

7o

(3*S*,4*R*)-3-((Benzo[d][1,3]dioxol-5-yloxy)methyl)-4-(4-fluorophenyl)-1-tosylpiperidine (**7o**). yellow liquid, ^{1}H NMR (400 MHz, $CDCl_3$) δ 7.69 (d, *J* = 8.1 Hz, 2H), 7.34 (d, *J* = 8.0 Hz, 2H), 7.08 (dd, *J* = 8.2, 5.6 Hz, 2H), 6.95 (t, *J* = 8.6 Hz, 2H), 6.61 (d, *J* = 8.5 Hz, 1H), 6.31 (d, *J* = 2.3 Hz, 1H), 6.10 (dd, *J* = 8.5, 2.3 Hz, 1H), 5.86 (s, 2H), 4.13 (d, *J* = 9.9 Hz, 1H), 3.93 (d, *J* = 11.5 Hz, 1H), 3.57 (dd, *J* = 9.5, 2.2 Hz, 1H), 3.39 (dd, *J* = 9.4, 6.5 Hz, 1H), 2.51 – 2.41 (m, 4H), 2.39 – 2.30 (m, 2H), 2.22 (ddd, *J* = 18.4, 10.7, 5.1 Hz, 1H), 1.90 (tt, *J* = 13.0, 6.8 Hz, 2H). ^{19}F NMR (376 MHz, $CDCl_3$) δ -115.66. ^{13}C NMR (101 MHz, $CDCl_3$) δ 161.6 (d, *J* = 245.4 Hz), 153.9, 148.1, 143.5, 141.7, 138.3 (d, *J* = 3.0 Hz), 133.0, 129.6, 128.6 (d, *J* = 8.1 Hz), 127.7, 115.5 (d, *J* = 21.2 Hz), 107.7, 105.4, 101.0, 97.8, 68.5, 49.4, 46.6, 43.0, 41.6, 33.2, 21.4.

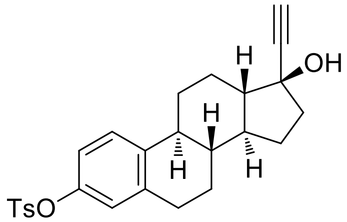

7p

(8*R*,9*S*,13*R*,14*S*)-17-ethynyl-7,8,9,11,12,13,14,15,16,17-decahydro-6*H*-cyclopenta [*a*]phenanthrene-3,13-diol (**7p**). yellow liquid, ^{1}H NMR (400 MHz, $CDCl_3$) δ 7.73 (d, *J* = 8.0 Hz, 2H), 7.31 (d, *J* = 7.9 Hz, 2H), 7.16 (d, *J* = 8.6 Hz, 1H), 6.74 (s, 1H), 6.67 (d, *J* = 8.5 Hz, 1H), 2.77 (d, *J* = 4.5 Hz, 2H), 2.59 (s, 1H), 2.45 (s, 3H), 2.37 – 2.27 (m, 2H), 2.24 – 2.12 (m, 2H), 2.06 – 1.97 (m, 1H), 1.95 – 1.64 (m, 6H), 1.46 – 1.32 (m, 4H). ^{13}C NMR (101 MHz, $CDCl_3$) δ 147.3, 145.1, 139.1, 138.5, 132.7, 129.6, 128.4, 126.4, 122.2, 119.0, 87.4, 79.7, 74.1, 49.4, 46.9, 43.6, 38.8, 38.8, 32.6, 29.4, 26.8, 26.1, 22.7, 21.6, 12.6.

2. ^{1}H NMR and ^{13}C NMR for all Compounds

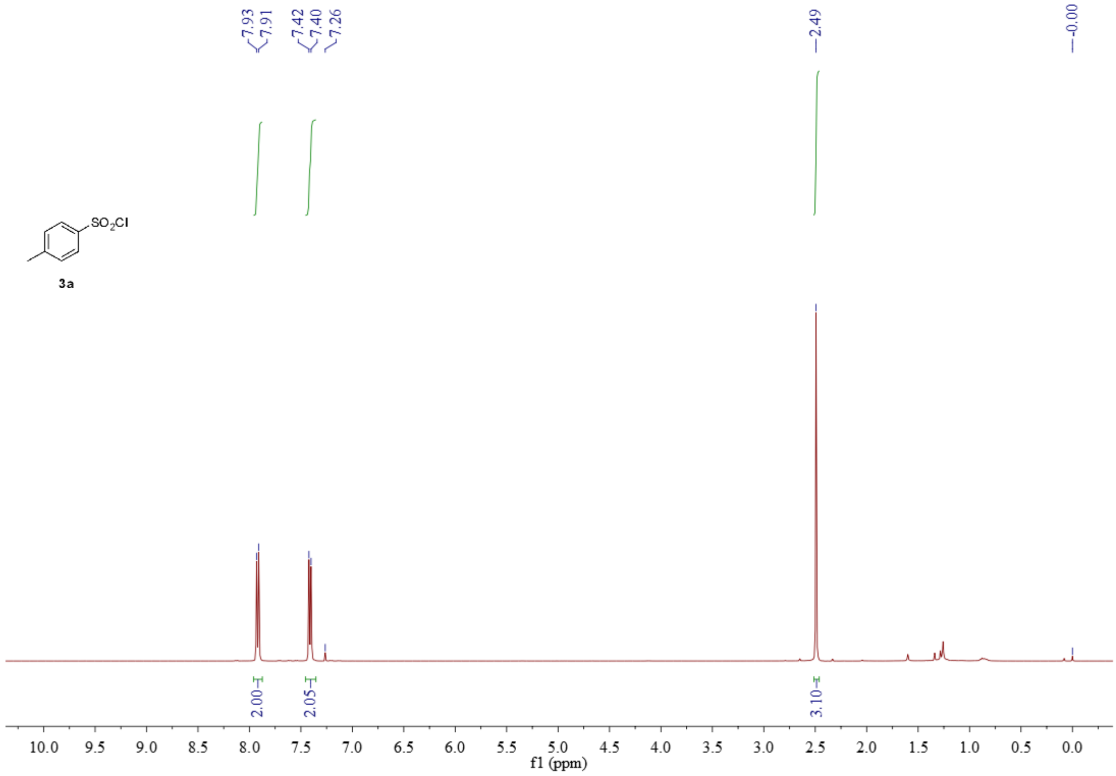

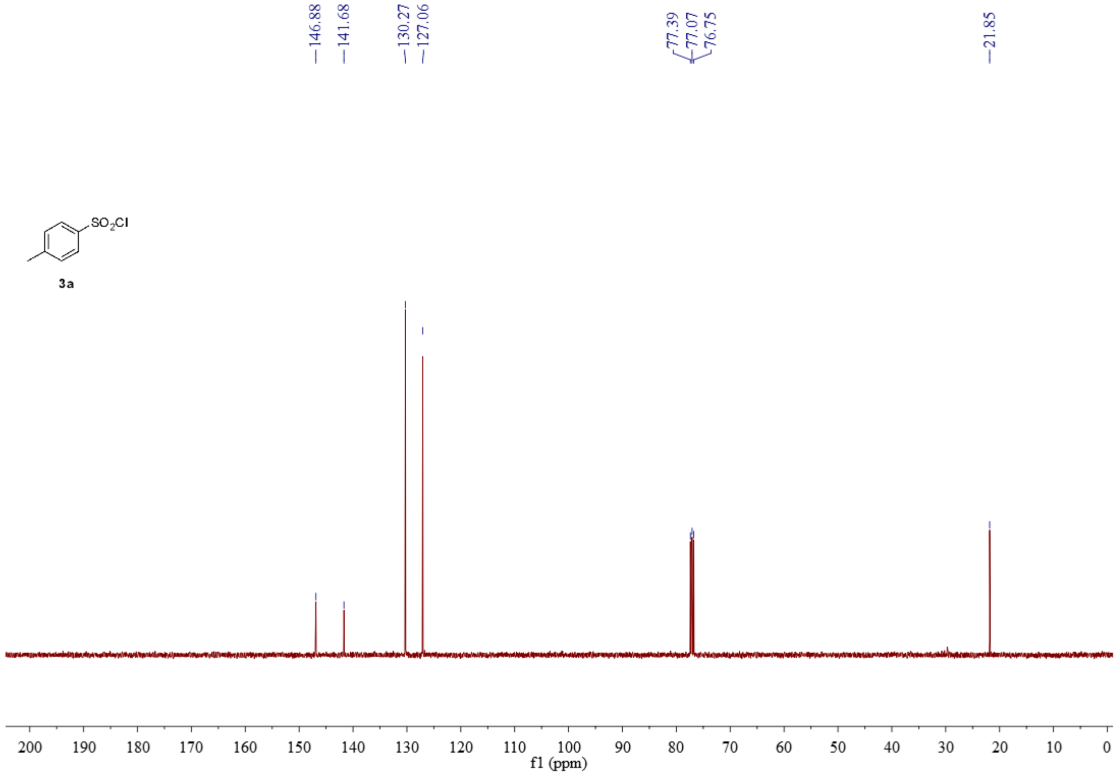

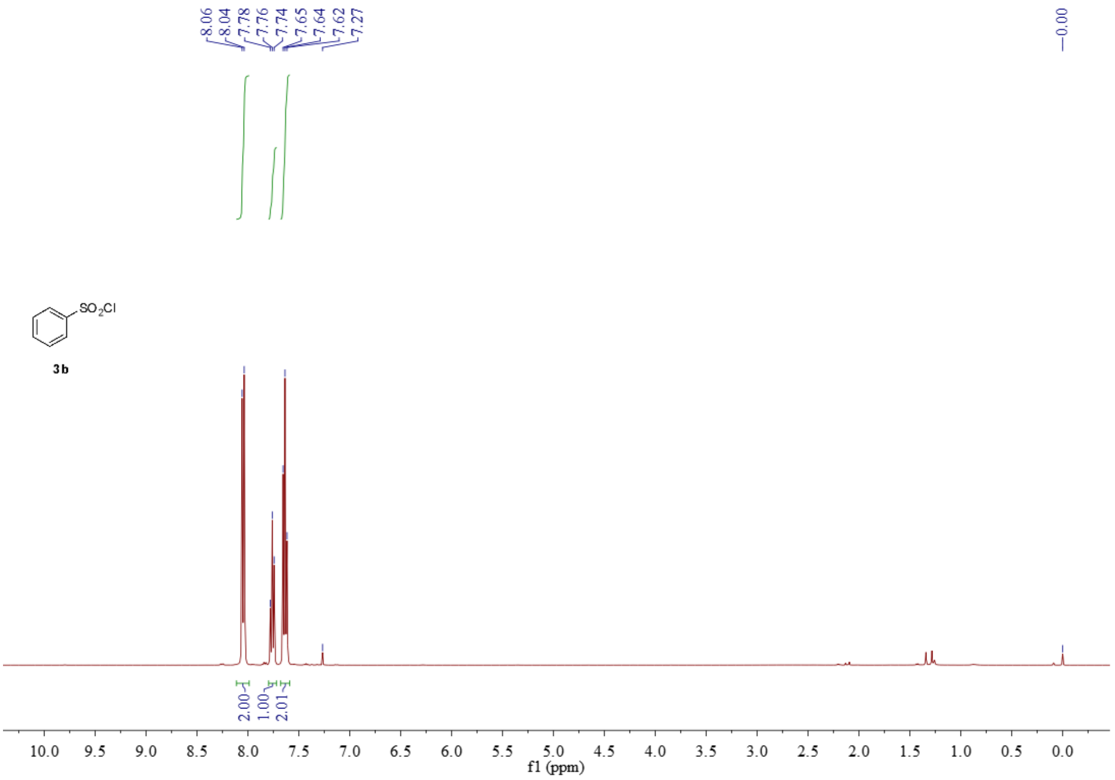
8.06
8.04
7.78
7.76
7.74
7.65
7.64
7.62
7.27
0.00
SO2Cl
3b
2.00
1.00
2.01
f1 (ppm)

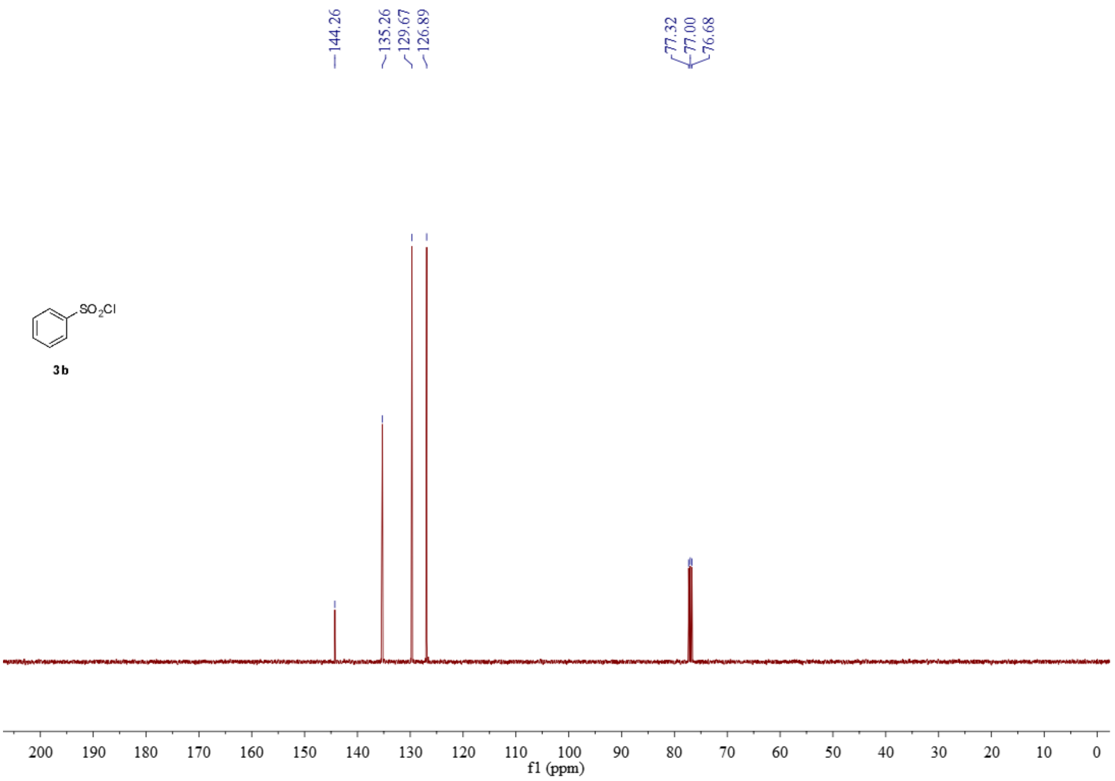
144.26
135.26
129.67
126.89
77.32
77.00
76.68
SO2Cl
3b
f1 (ppm)

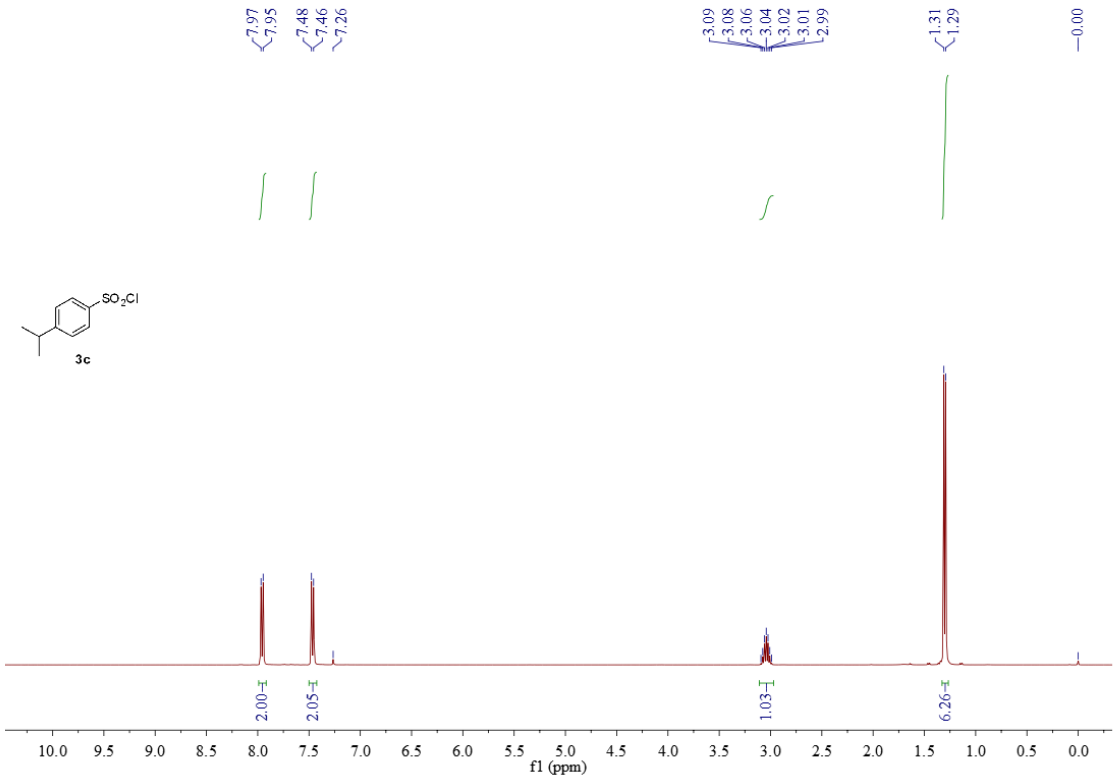
7.97
7.95
7.48
7.46
7.26
3.09
3.08
3.06
3.04
3.02
3.01
2.99
1.31
1.29
0.00
SO2Cl
3c
2.00
2.05
1.03
6.26
10.0 9.5 9.0 8.5 8.0 7.5 7.0 6.5 6.0 5.5 5.0 4.5 4.0 3.5 3.0 2.5 2.0 1.5 1.0 0.5 0.0
f1 (ppm)

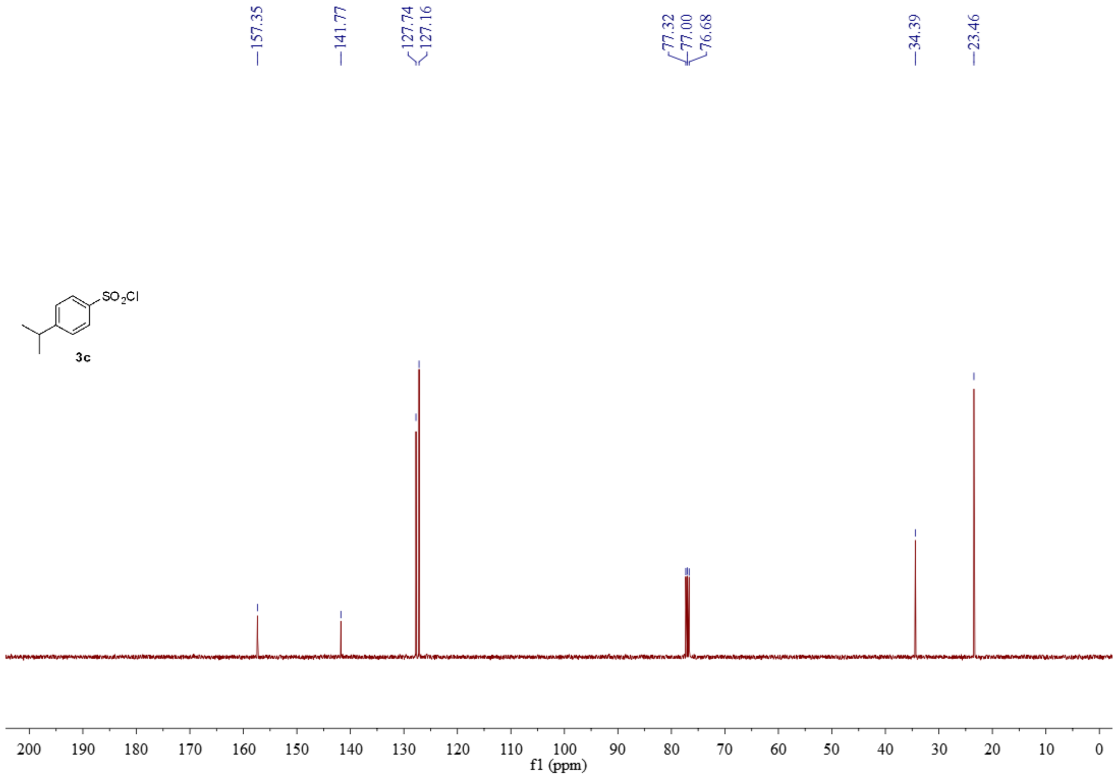
157.35
141.77
127.74
127.16
77.32
77.00
76.68
34.39
23.46
SO2Cl
3c
200 190 180 170 160 150 140 130 120 110 100 90 80 70 60 50 40 30 20 10 0
f1 (ppm)

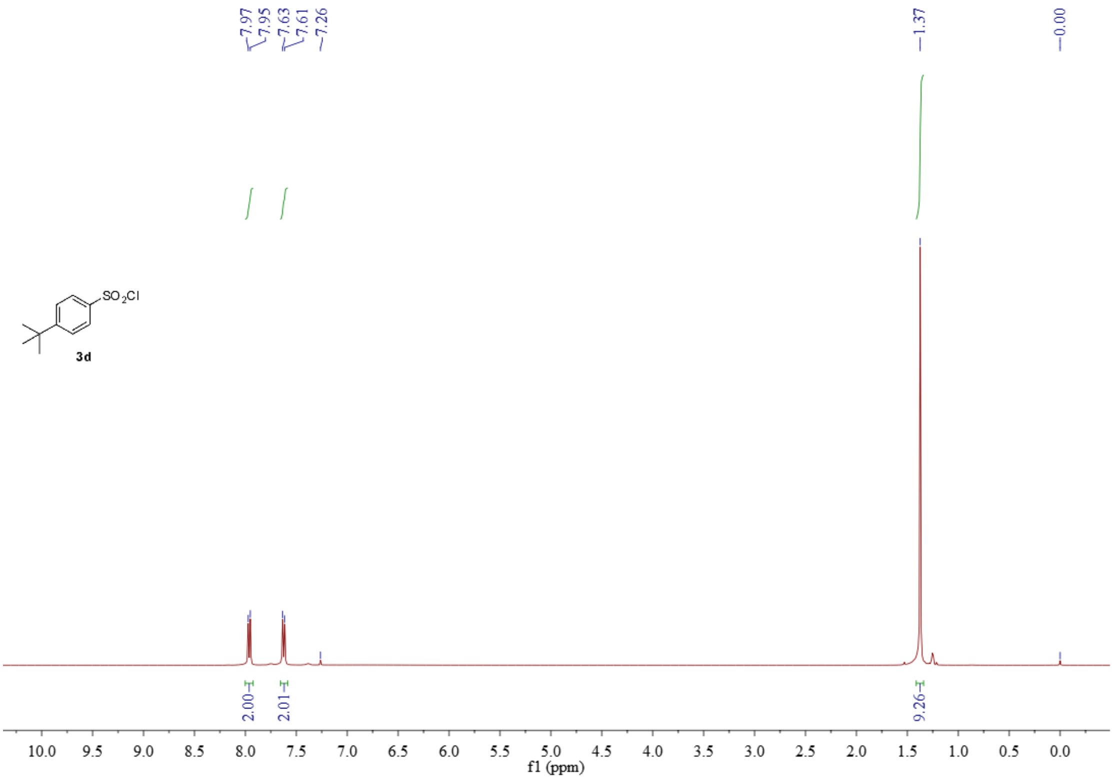
7.97
7.95
7.63
7.61
7.26
1.37
0.00
SO2Cl
3d
2.00
2.01
9.26
10.0 9.5 9.0 8.5 8.0 7.5 7.0 6.5 6.0 5.5 5.0 4.5 4.0 3.5 3.0 2.5 2.0 1.5 1.0 0.5 0.0
f1 (ppm)

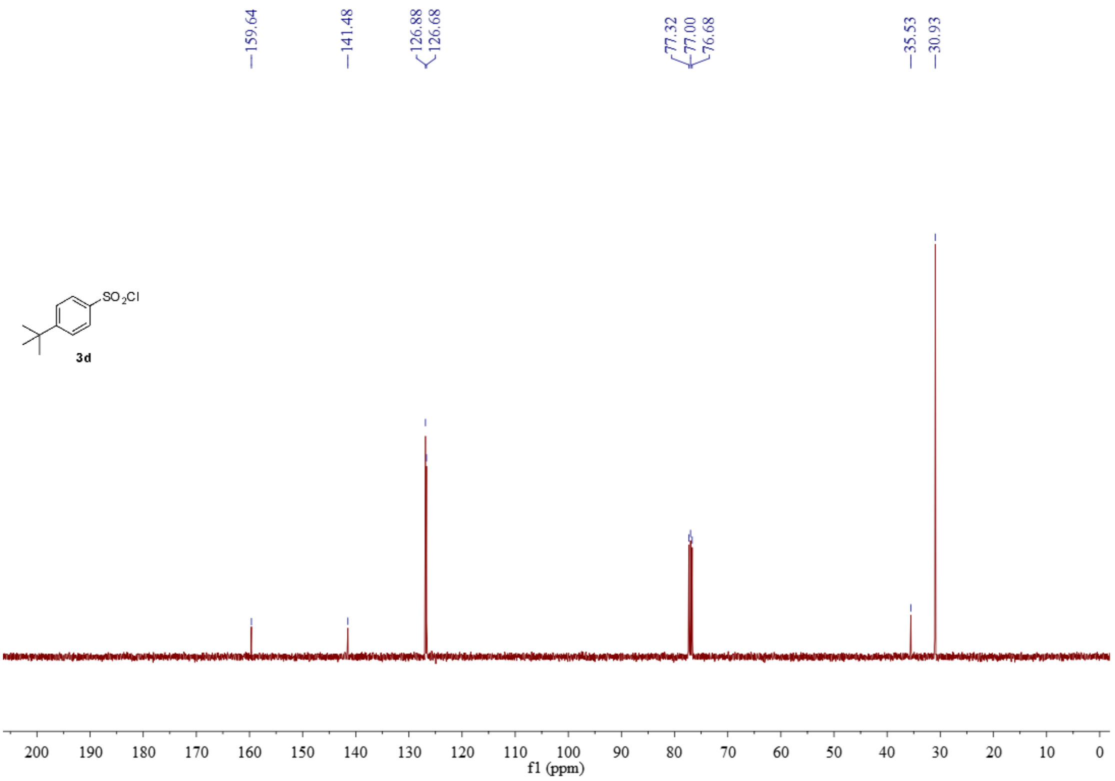
159.64
141.48
126.88
126.68
77.32
77.00
76.68
35.53
30.93
SO2Cl
3d
200 190 180 170 160 150 140 130 120 110 100 90 80 70 60 50 40 30 20 10 0
f1 (ppm)

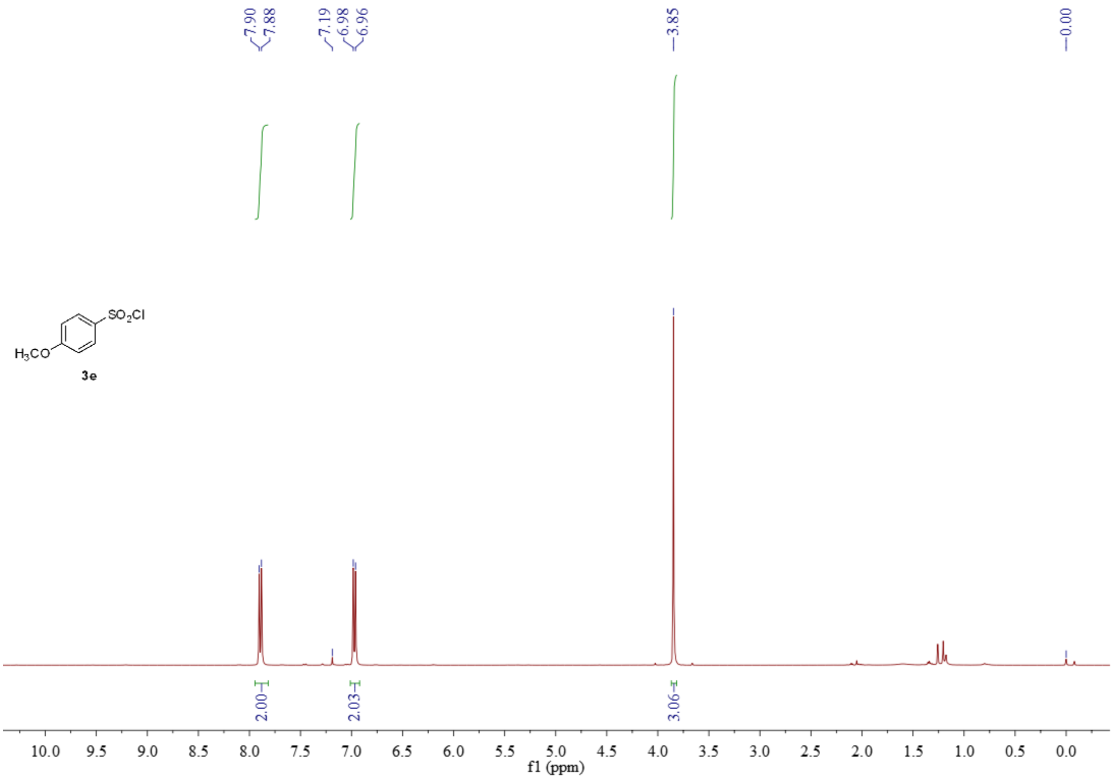
7.90
7.88
7.19
6.98
6.96
3.85
0.00
SO2Cl
H3CO
3e
2.00
2.03
3.06
f1 (ppm)

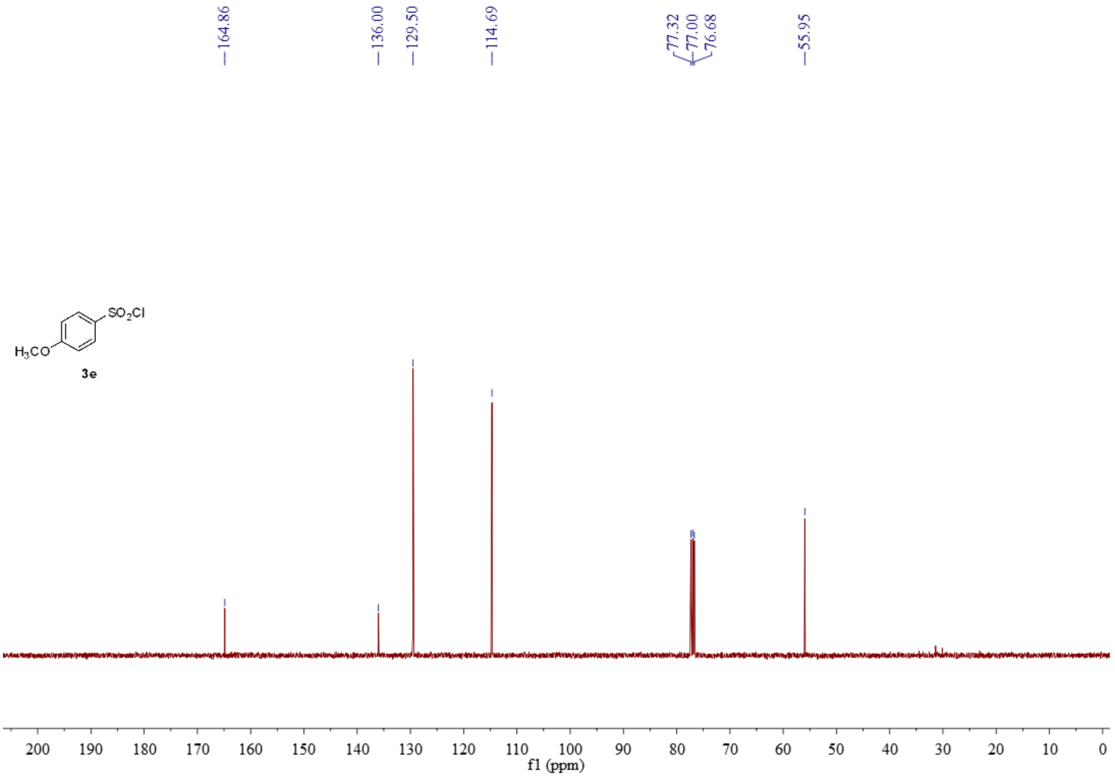
164.86
136.00
129.50
114.69
77.32
77.00
76.68
55.95
SO2Cl
H3CO
3e
f1 (ppm)

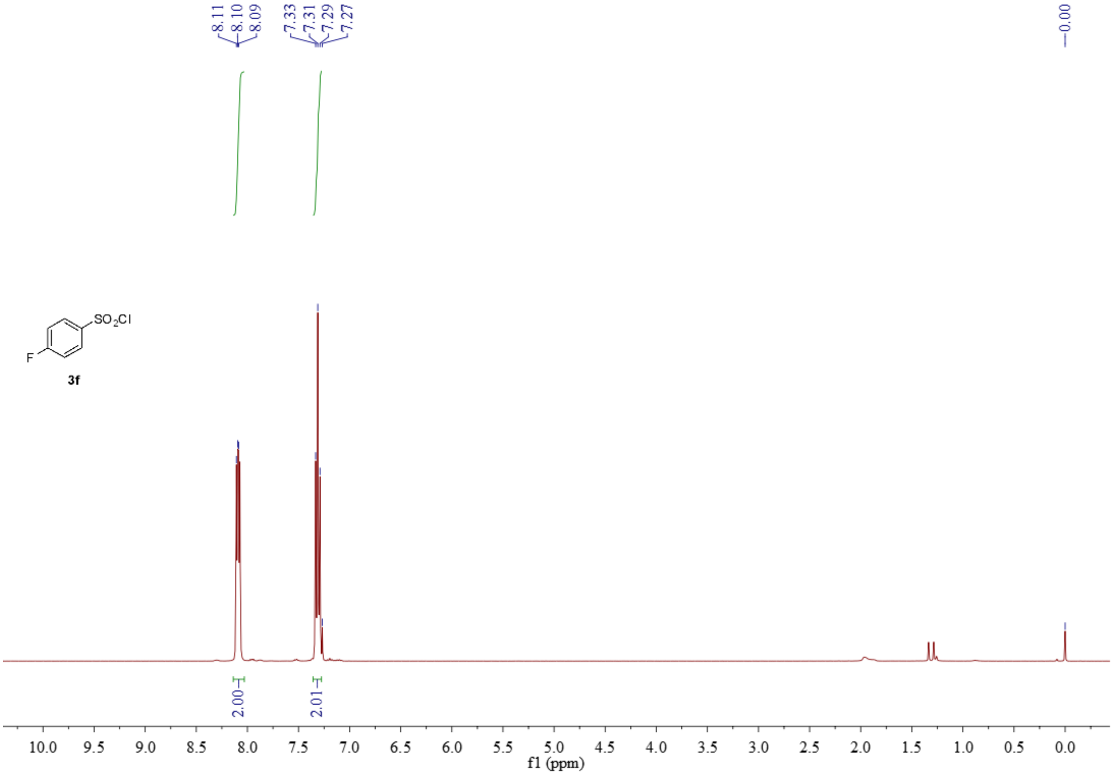
8.11
8.10
8.09
7.33
7.31
7.29
7.27
0.00
SO2Cl
F
2.00
2.01
10.0 9.5 9.0 8.5 8.0 7.5 7.0 6.5 6.0 5.5 5.0 4.5 4.0 3.5 3.0 2.5 2.0 1.5 1.0 0.5 0.0
f1 (ppm)

3f

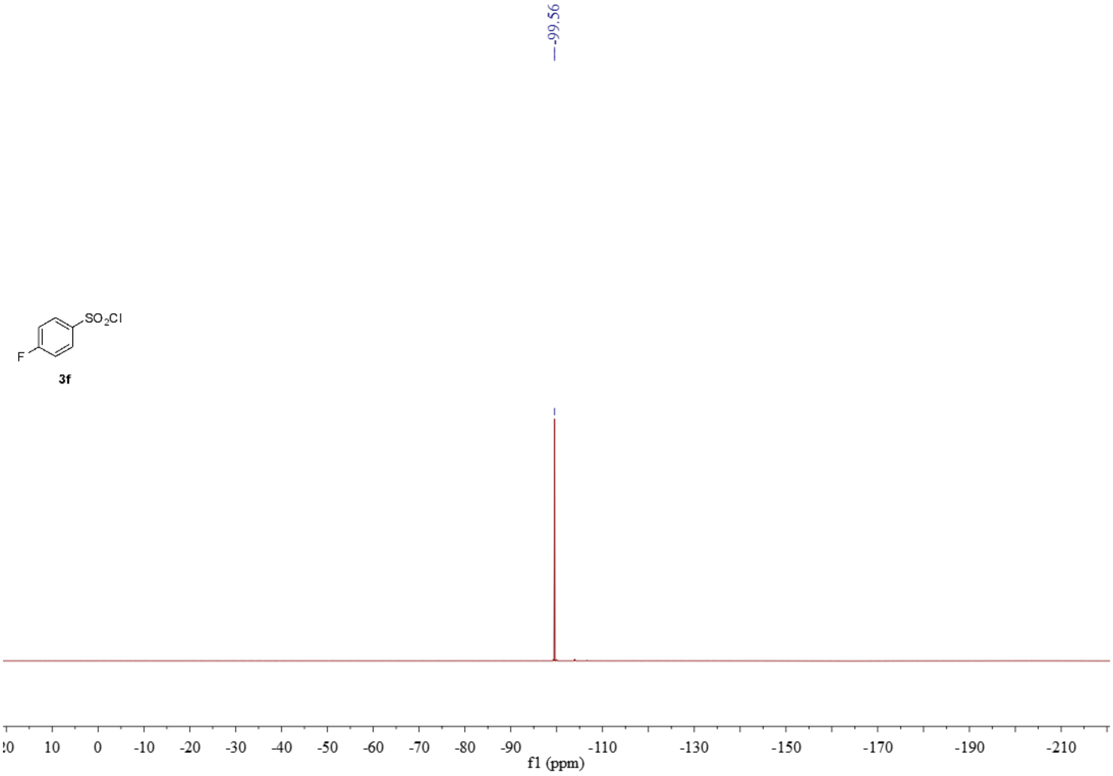
-99.56
SO2Cl
F
10 0 -10 -20 -30 -40 -50 -60 -70 -80 -90 -110 -130 -150 -170 -190 -210
f1 (ppm)

3f

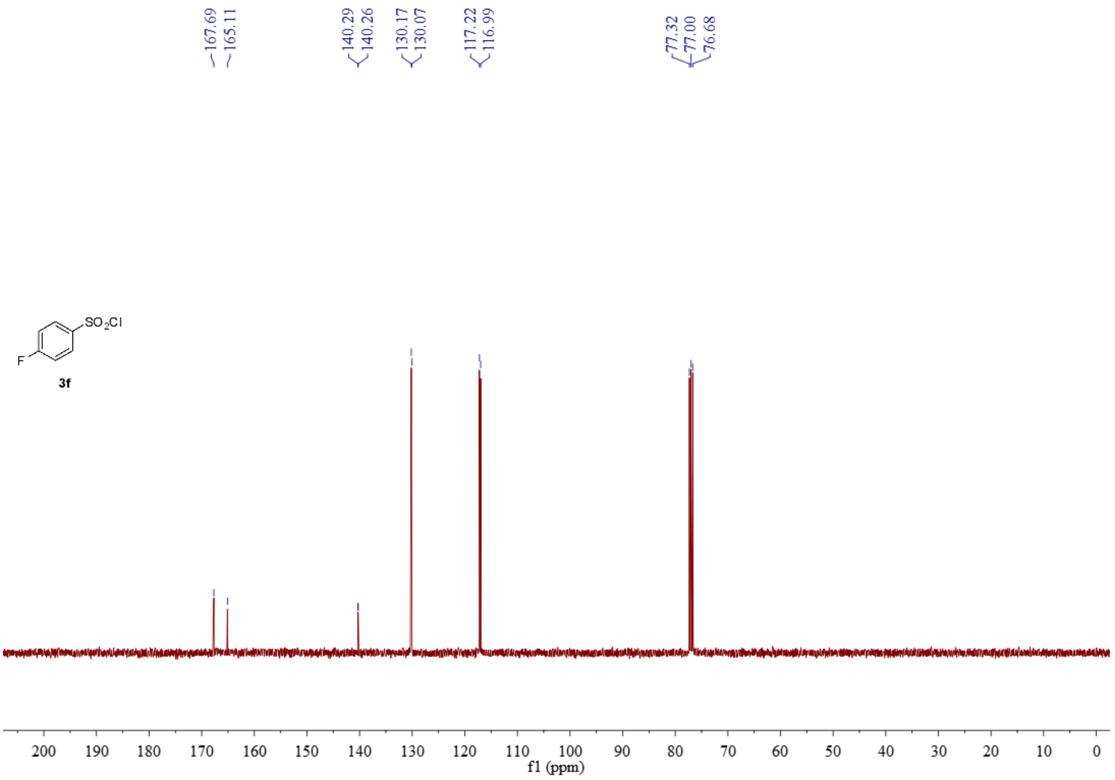
167.69
165.11
140.29
140.26
130.17
130.07
117.22
116.99
77.32
77.00
76.68
SO2Cl
F
3f
200 190 180 170 160 150 140 130 120 110 100 90 80 70 60 50 40 30 20 10 0
f1 (ppm)

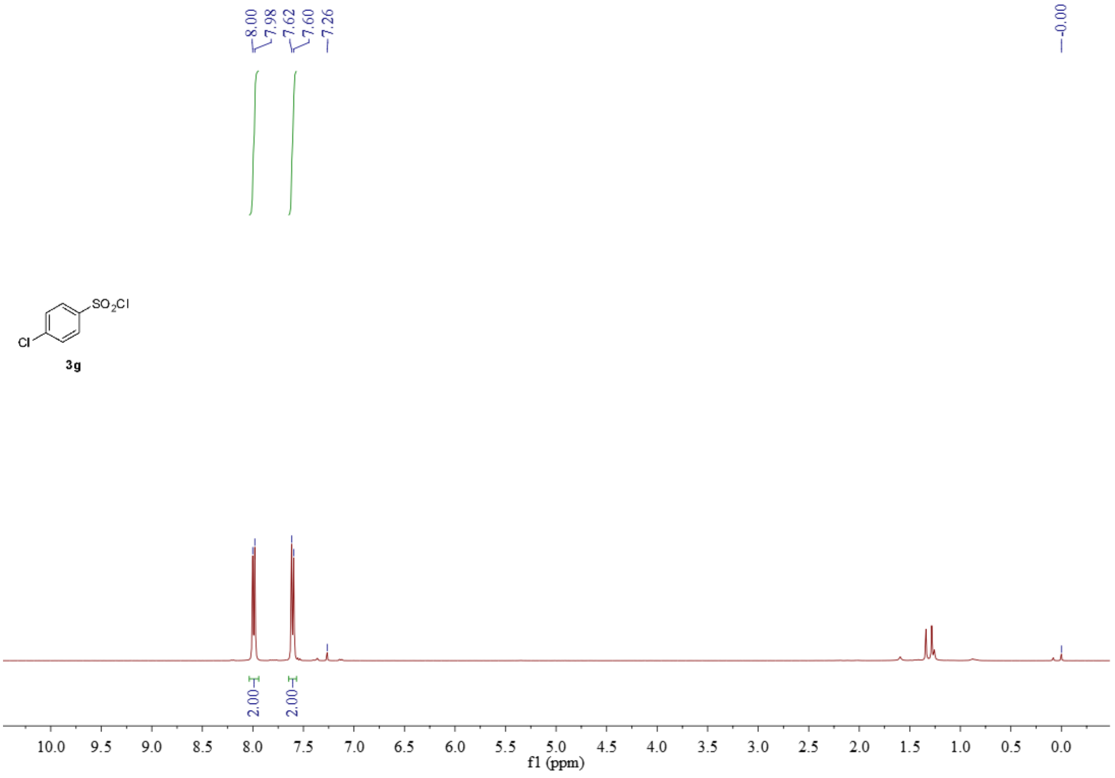
8.00
7.98
7.62
7.60
7.26
0.00
SO2Cl
Cl
3g
2.00
2.00
10.0 9.5 9.0 8.5 8.0 7.5 7.0 6.5 6.0 5.5 5.0 4.5 4.0 3.5 3.0 2.5 2.0 1.5 1.0 0.5 0.0
f1 (ppm)

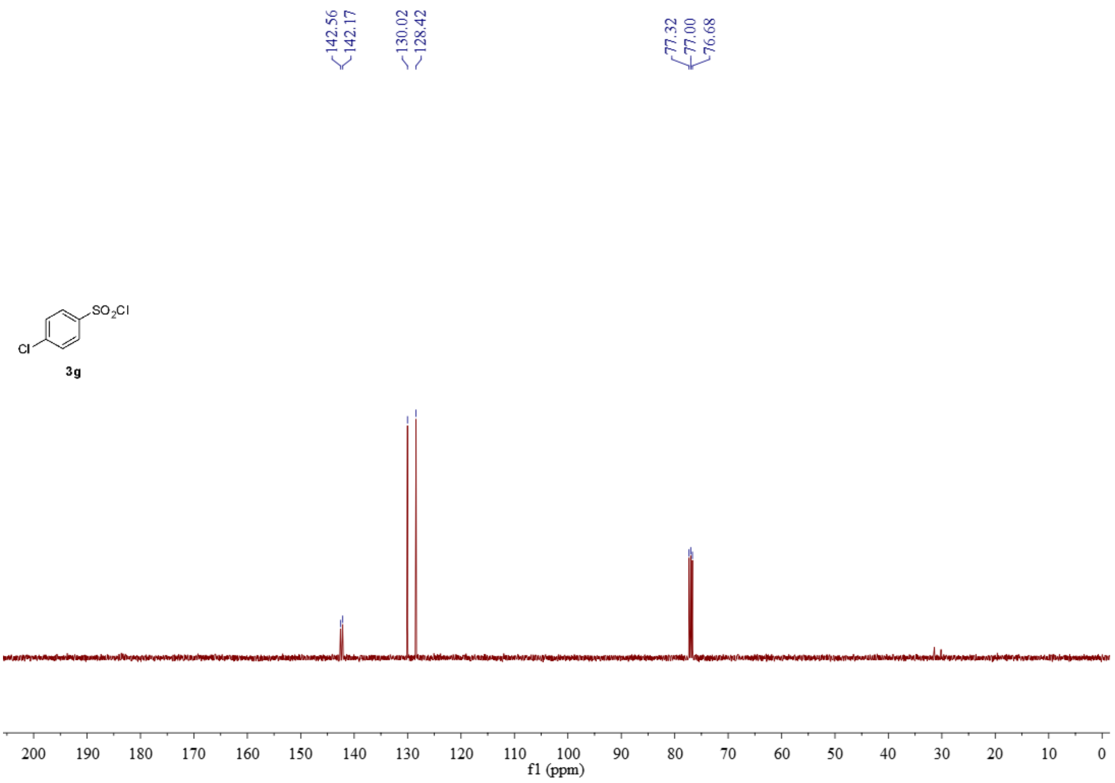
142.56
142.17
130.02
128.42
77.32
77.00
76.68
SO_2Cl
Cl
3g
200 190 180 170 160 150 140 130 120 110 100 90 80 70 60 50 40 30 20 10 0
f1 (ppm)

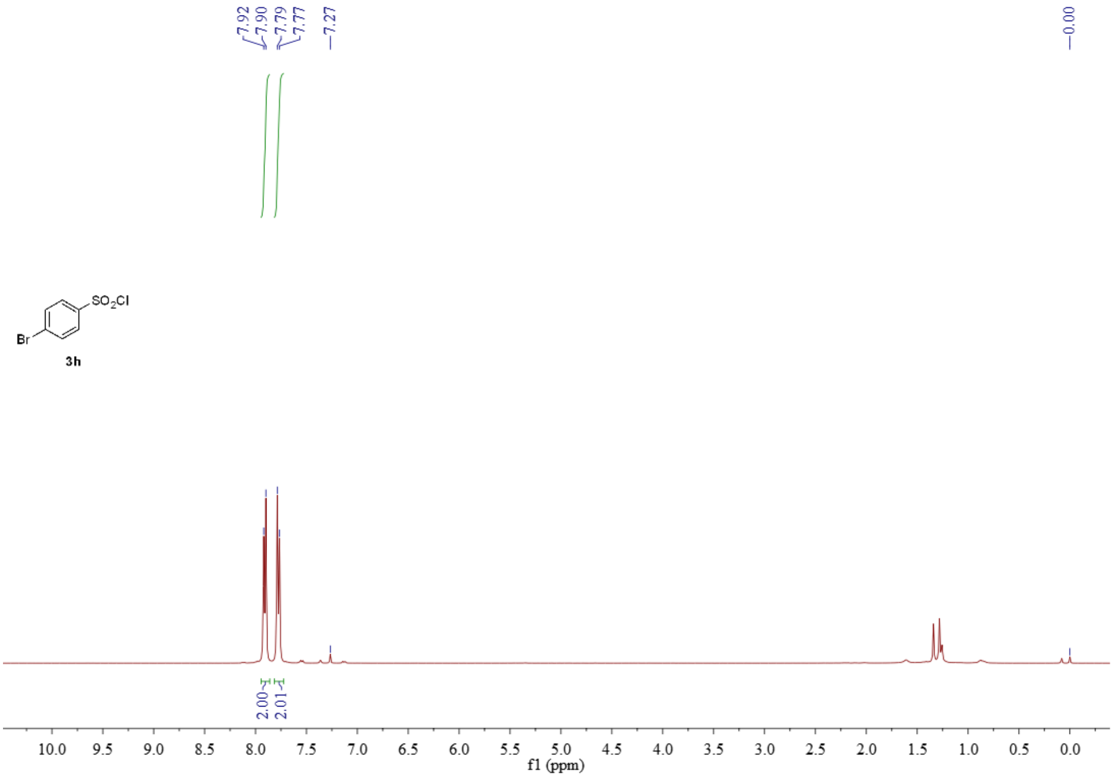
7.92
7.90
7.79
7.77
7.27
0.00
SO_2Cl
Br
3h
2.00
2.01
10.0 9.5 9.0 8.5 8.0 7.5 7.0 6.5 6.0 5.5 5.0 4.5 4.0 3.5 3.0 2.5 2.0 1.5 1.0 0.5 0.0
f1 (ppm)

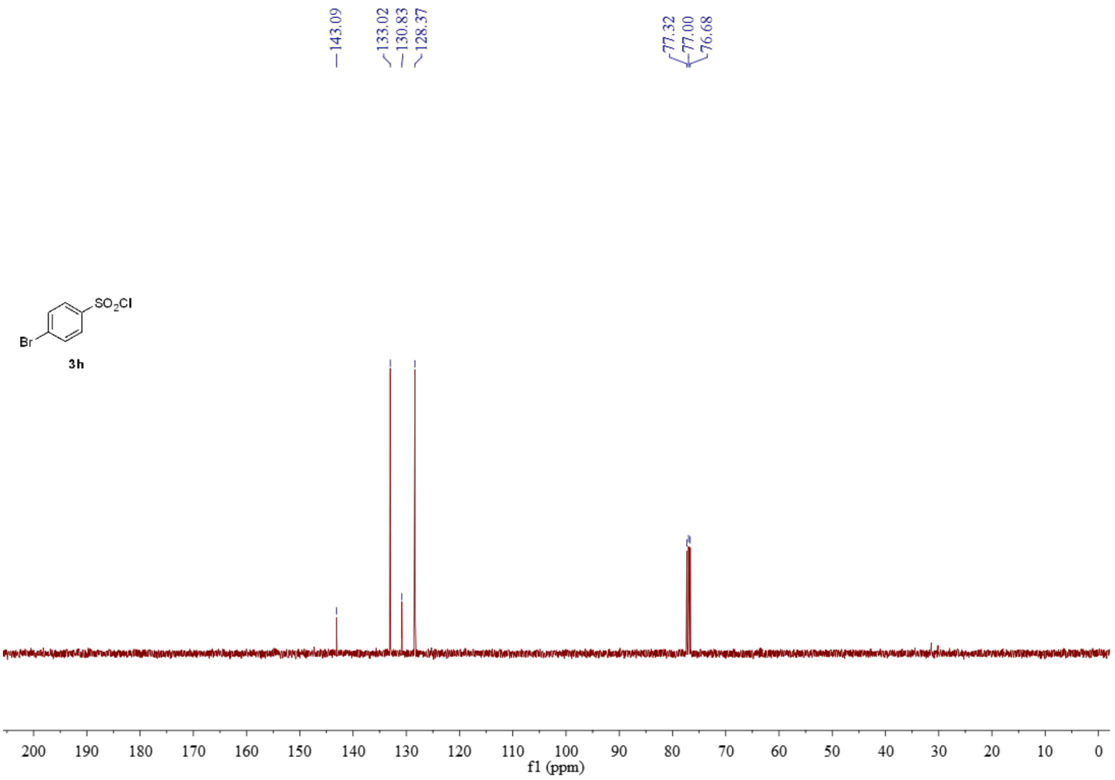
143.09
133.02
130.83
128.37
77.32
77.00
76.68
SO2Cl
Br
3h
200 190 180 170 160 150 140 130 120 110 100 90 80 70 60 50 40 30 20 10 0
f1 (ppm)

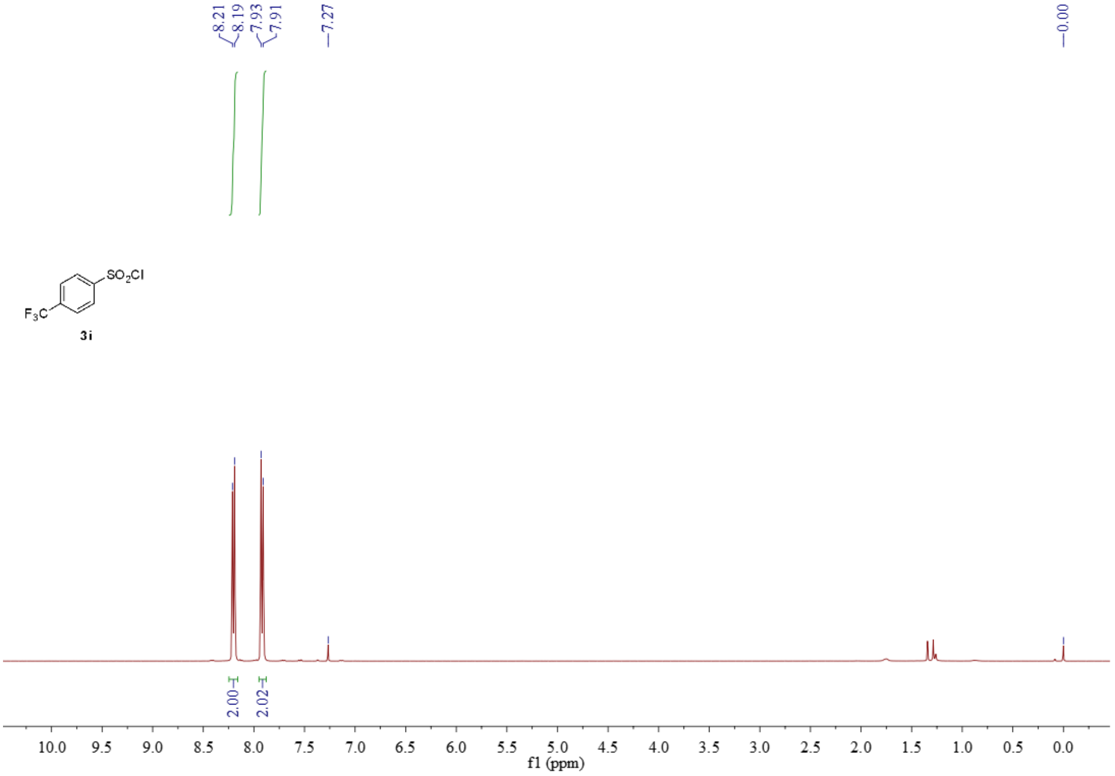
8.21
8.19
7.93
7.91
7.27
0.00
SO2Cl
F3C
3i
2.00
2.02
10.0 9.5 9.0 8.5 8.0 7.5 7.0 6.5 6.0 5.5 5.0 4.5 4.0 3.5 3.0 2.5 2.0 1.5 1.0 0.5 0.0
f1 (ppm)

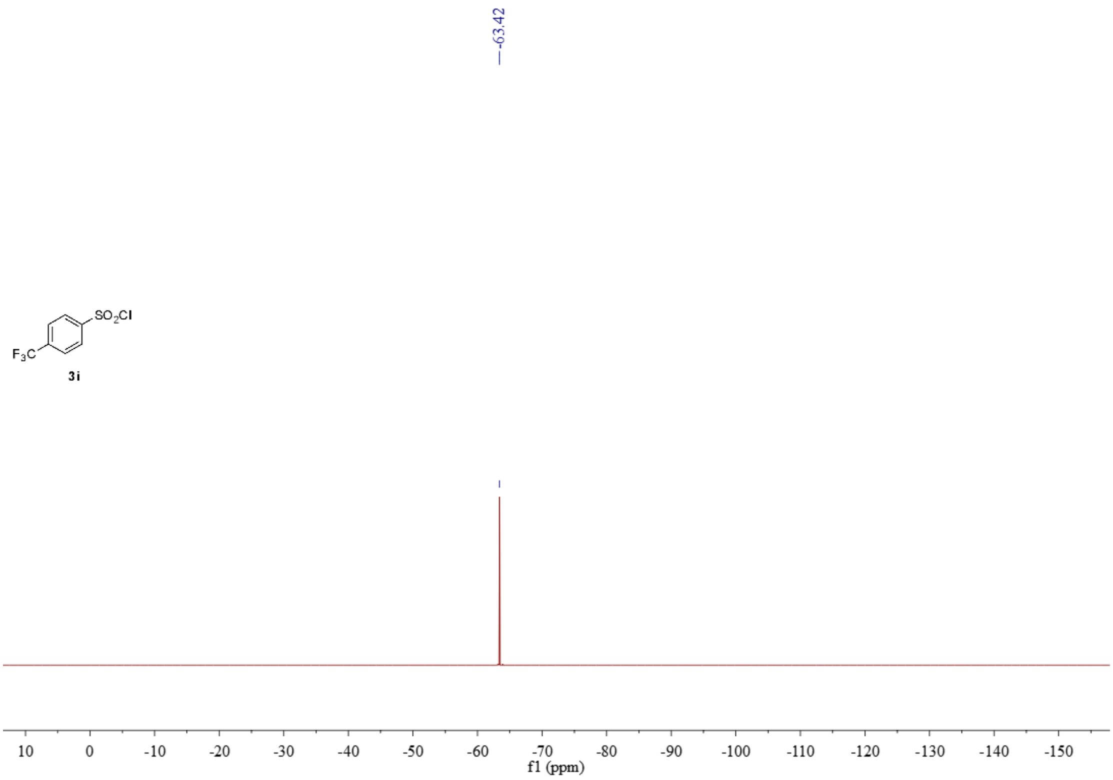
-63.42
SO2Cl
F3C
3i
10
0
-10
-20
-30
-40
-50
-60
-70
-80
-90
-100
-110
-120
-130
-140
-150
f1 (ppm)

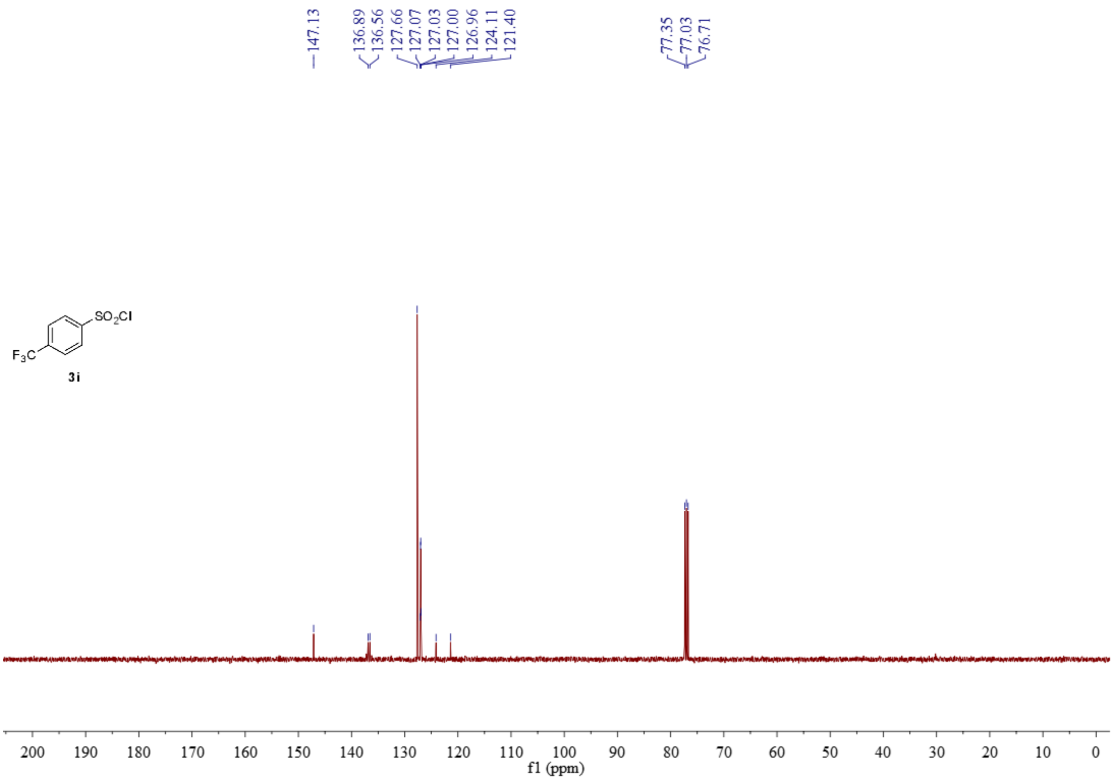
147.13
136.89
136.56
127.66
127.07
127.03
127.00
126.96
124.11
121.40
77.35
77.03
76.71
SO2Cl
F3C
3i
200
190
180
170
160
150
140
130
120
110
100
90
80
70
60
50
40
30
20
10
0
f1 (ppm)

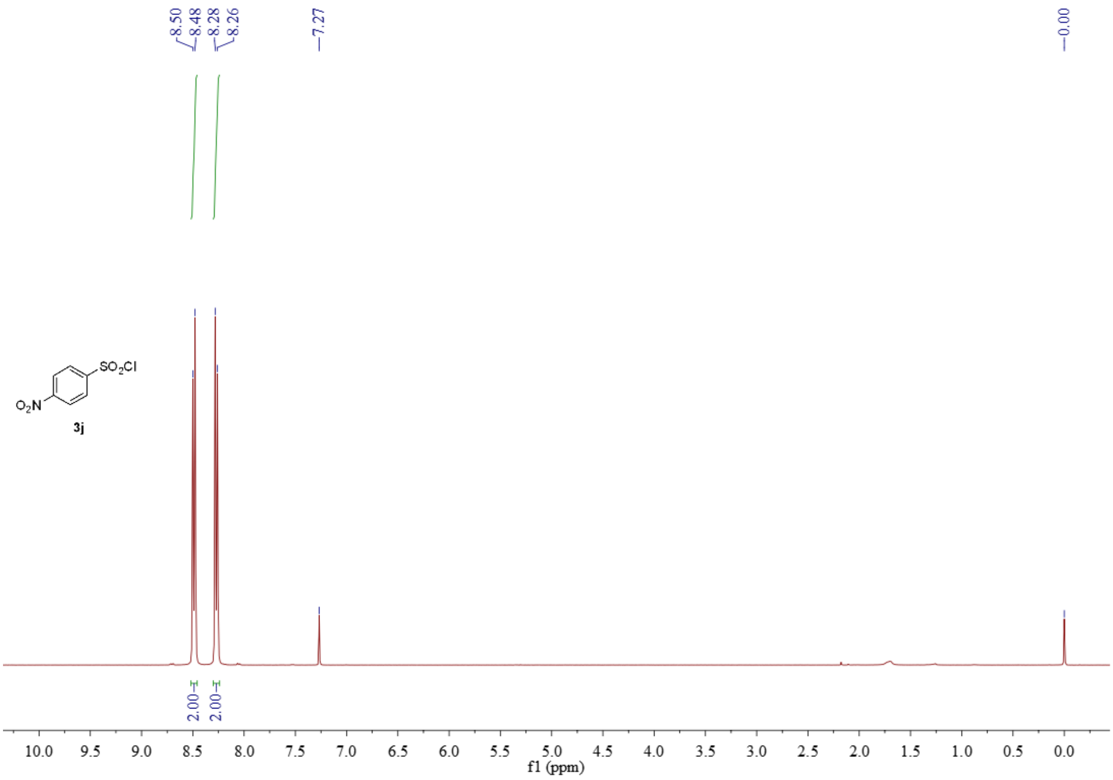
8.50
8.48
8.28
8.26
7.27
0.00
SO2Cl
O2N
3j
2.00
2.00
10.0 9.5 9.0 8.5 8.0 7.5 7.0 6.5 6.0 5.5 5.0 4.5 4.0 3.5 3.0 2.5 2.0 1.5 1.0 0.5 0.0
f1 (ppm)

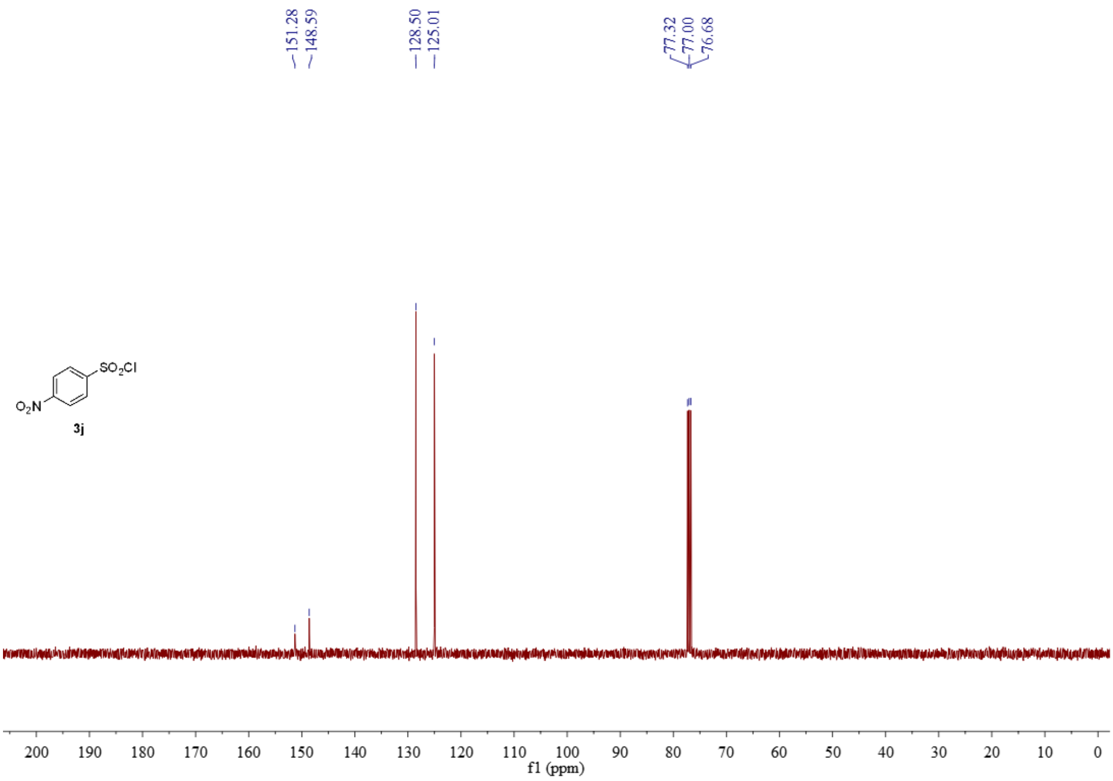
151.28
148.59
128.50
125.01
77.32
77.00
76.68
SO2Cl
O2N
3j
200 190 180 170 160 150 140 130 120 110 100 90 80 70 60 50 40 30 20 10 0
f1 (ppm)

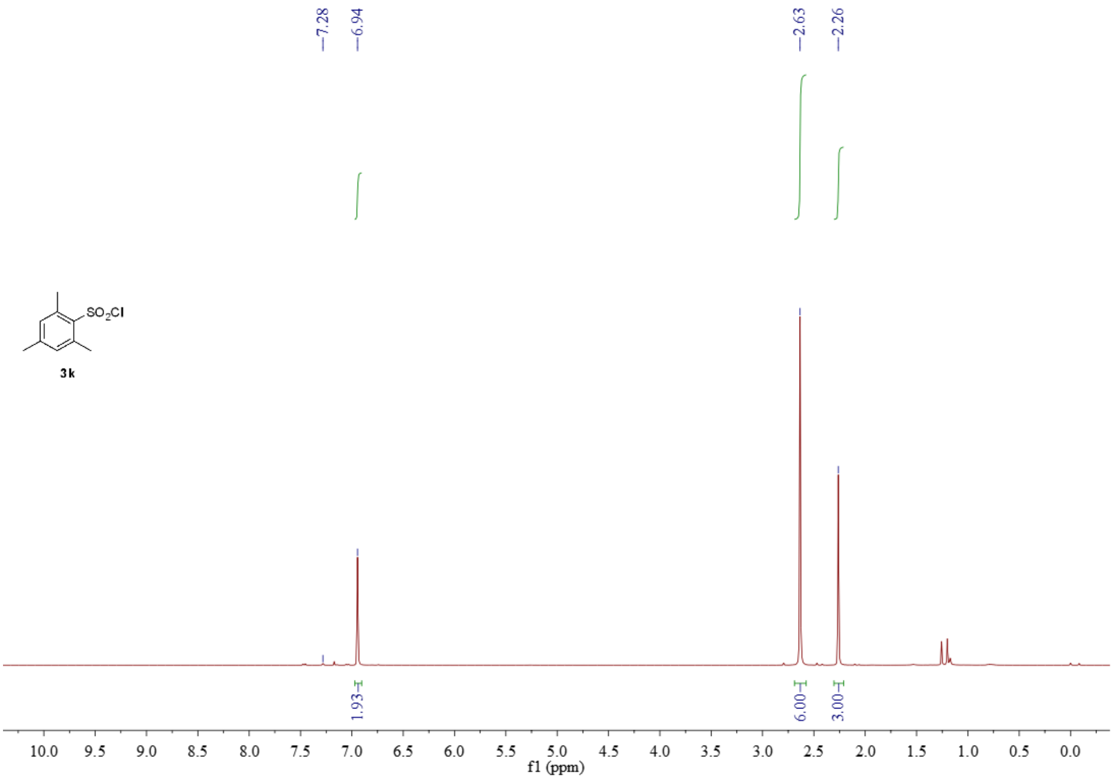
7.28
6.94
2.63
2.26
SO_2Cl
3k
1.93
6.00
3.00
10.0 9.5 9.0 8.5 8.0 7.5 7.0 6.5 6.0 5.5 5.0 4.5 4.0 3.5 3.0 2.5 2.0 1.5 1.0 0.5 0.0
f1 (ppm)

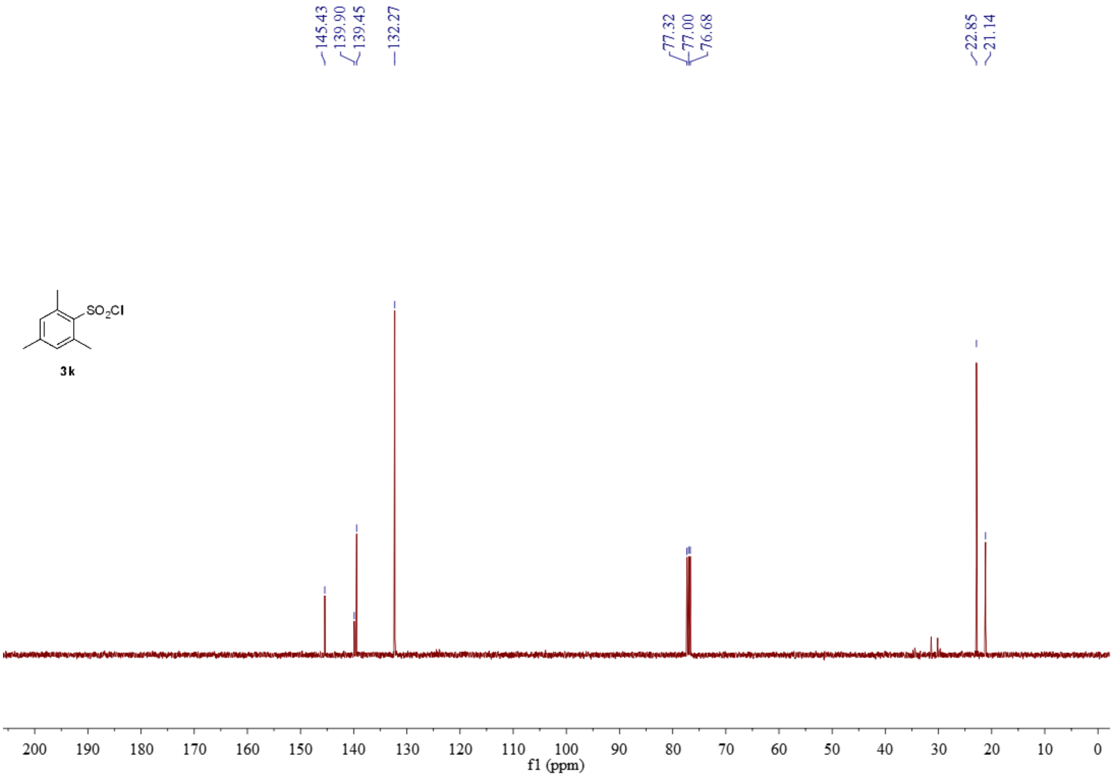
145.43
139.90
139.45
132.27
77.32
77.00
76.68
22.85
21.14
SO_2Cl
3k
200 190 180 170 160 150 140 130 120 110 100 90 80 70 60 50 40 30 20 10 0
f1 (ppm)

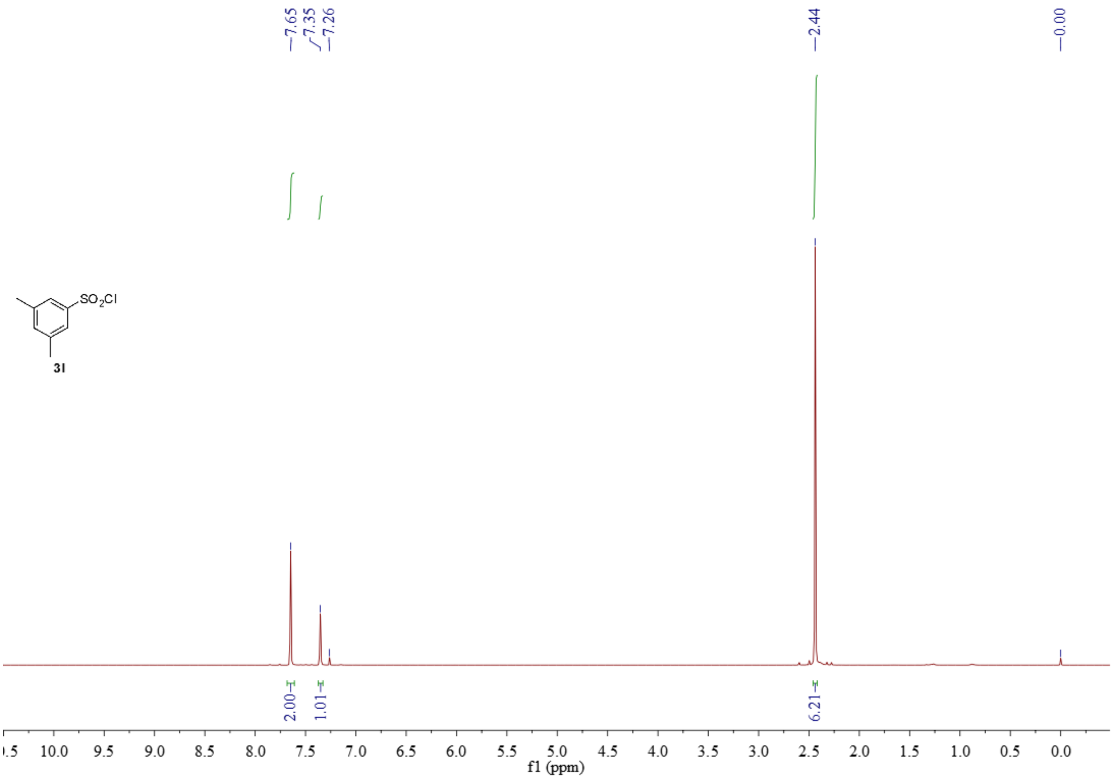
7.65
7.35
7.26
2.44
0.00
2.00
1.01
6.21
f1 (ppm)

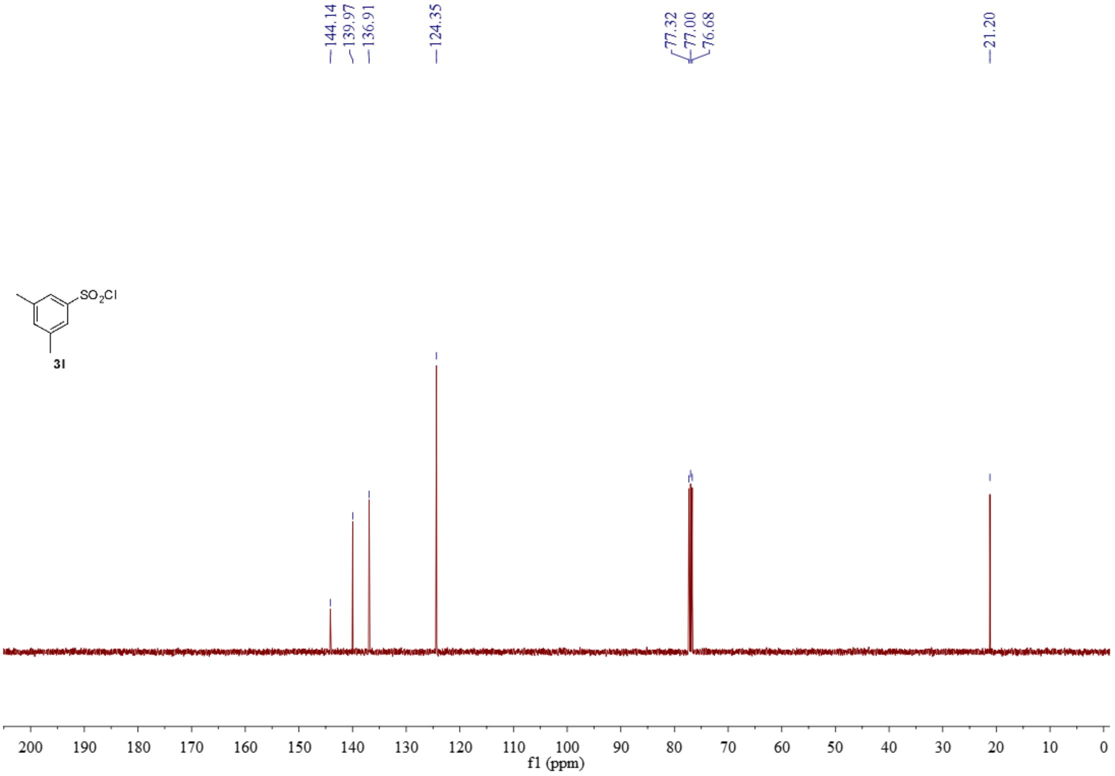
144.14
139.97
136.91
124.35
77.32
77.00
76.68
21.20
f1 (ppm)

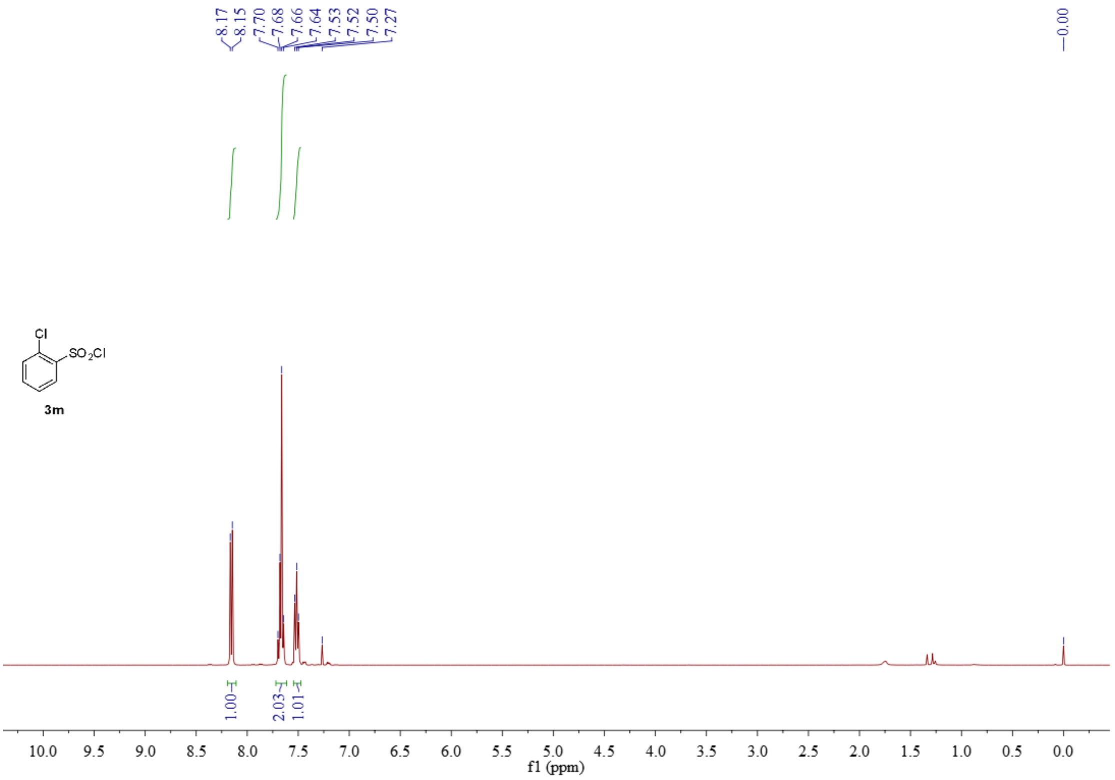
8.17
8.15
7.70
7.68
7.66
7.64
7.53
7.52
7.50
7.27
0.00
Cl
SO2Cl
3m
1.00
2.03
1.01
10.0 9.5 9.0 8.5 8.0 7.5 7.0 6.5 6.0 5.5 5.0 4.5 4.0 3.5 3.0 2.5 2.0 1.5 1.0 0.5 0.0
f1 (ppm)

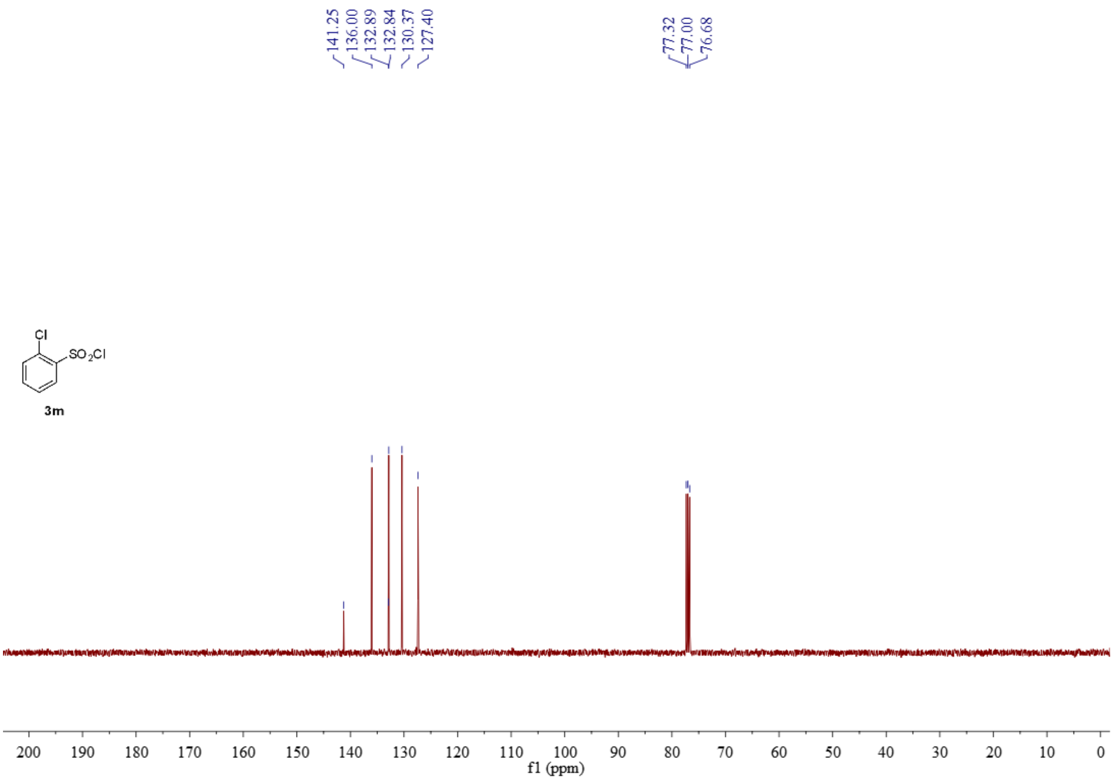
141.25
136.00
132.89
132.84
130.37
127.40
77.32
77.00
76.68
Cl
SO2Cl
3m
200 190 180 170 160 150 140 130 120 110 100 90 80 70 60 50 40 30 20 10 0
f1 (ppm)

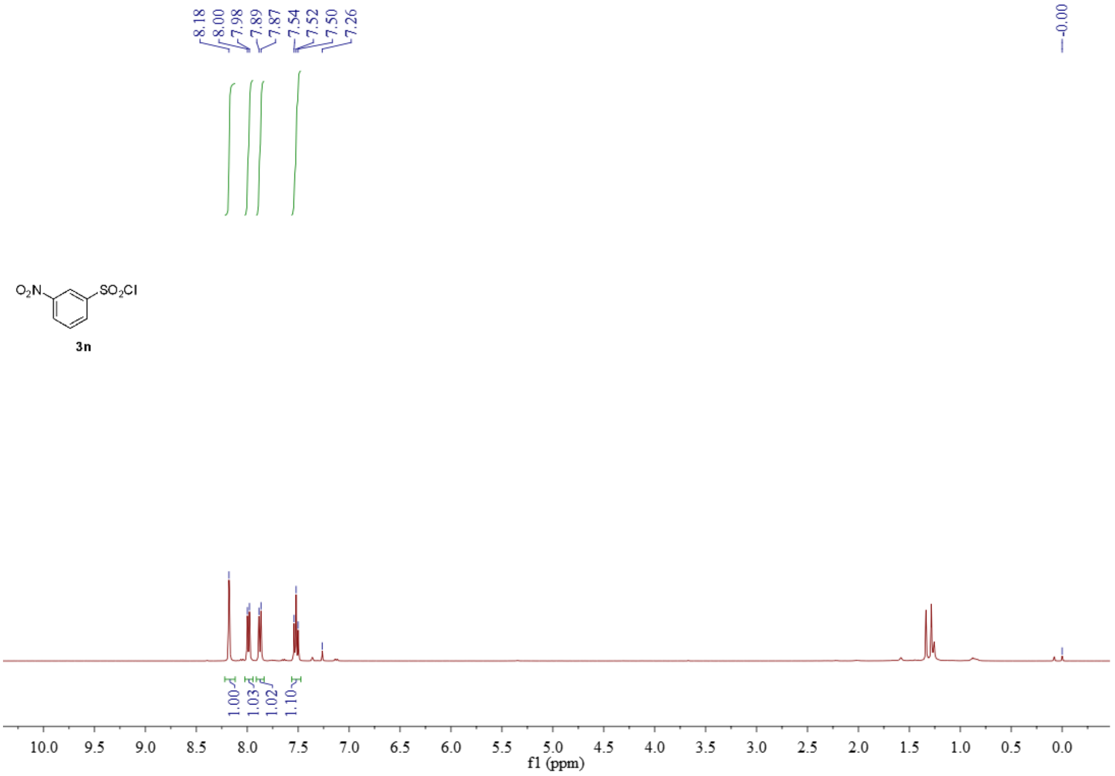
8.18
8.00
7.98
7.89
7.87
7.54
7.52
7.50
7.26
-0.00
O2N
SO2Cl
3n
1.00
1.03
1.02
1.10
10.0 9.5 9.0 8.5 8.0 7.5 7.0 6.5 6.0 5.5 5.0 4.5 4.0 3.5 3.0 2.5 2.0 1.5 1.0 0.5 0.0
f1 (ppm)

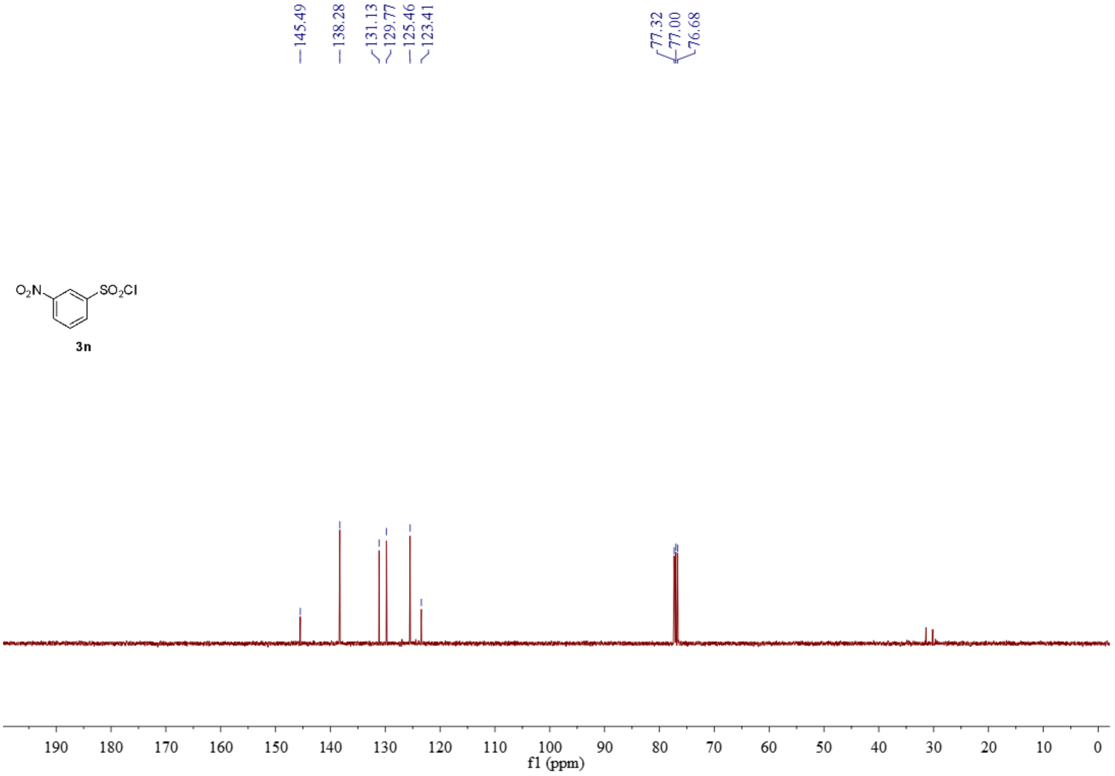
145.49
138.28
131.13
129.77
125.46
123.41
77.32
77.00
76.68
O2N
SO2Cl
3n
190 180 170 160 150 140 130 120 110 100 90 80 70 60 50 40 30 20 10 0
f1 (ppm)

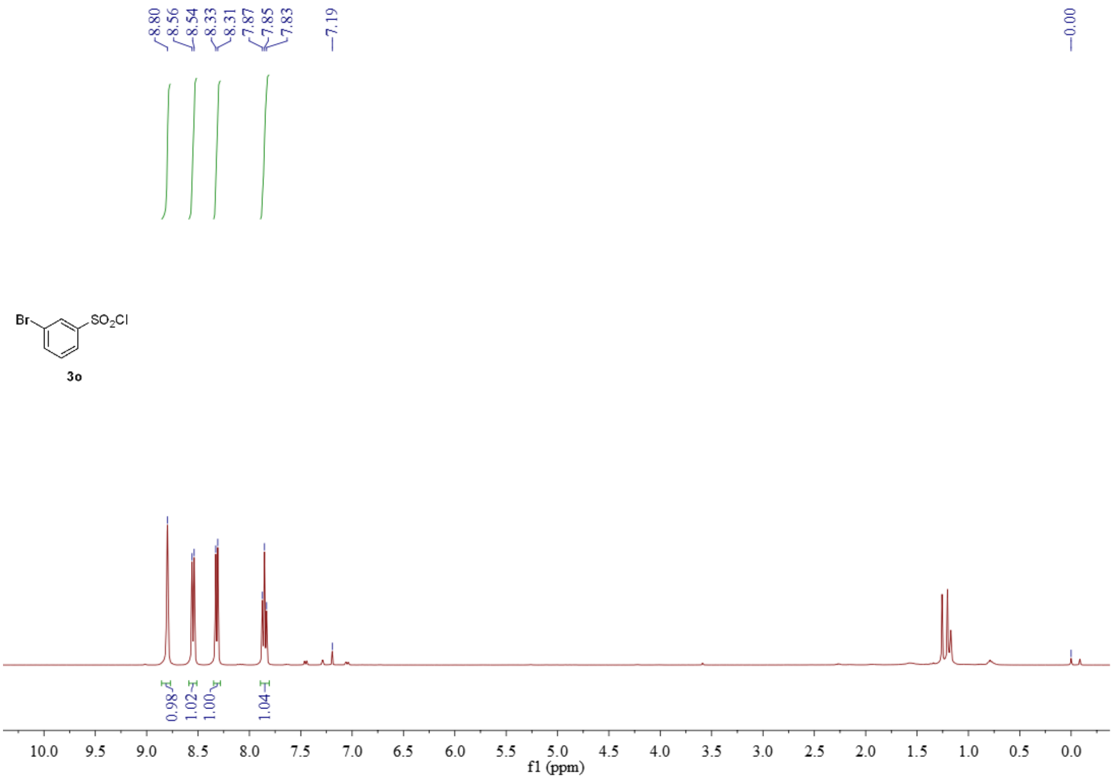
8.80
8.56
8.54
8.33
8.31
7.87
7.85
7.83
7.19
0.00
Br
SO2Cl
3o
0.98
1.02
1.00
1.04
10.0 9.5 9.0 8.5 8.0 7.5 7.0 6.5 6.0 5.5 5.0 4.5 4.0 3.5 3.0 2.5 2.0 1.5 1.0 0.5 0.0
f1 (ppm)

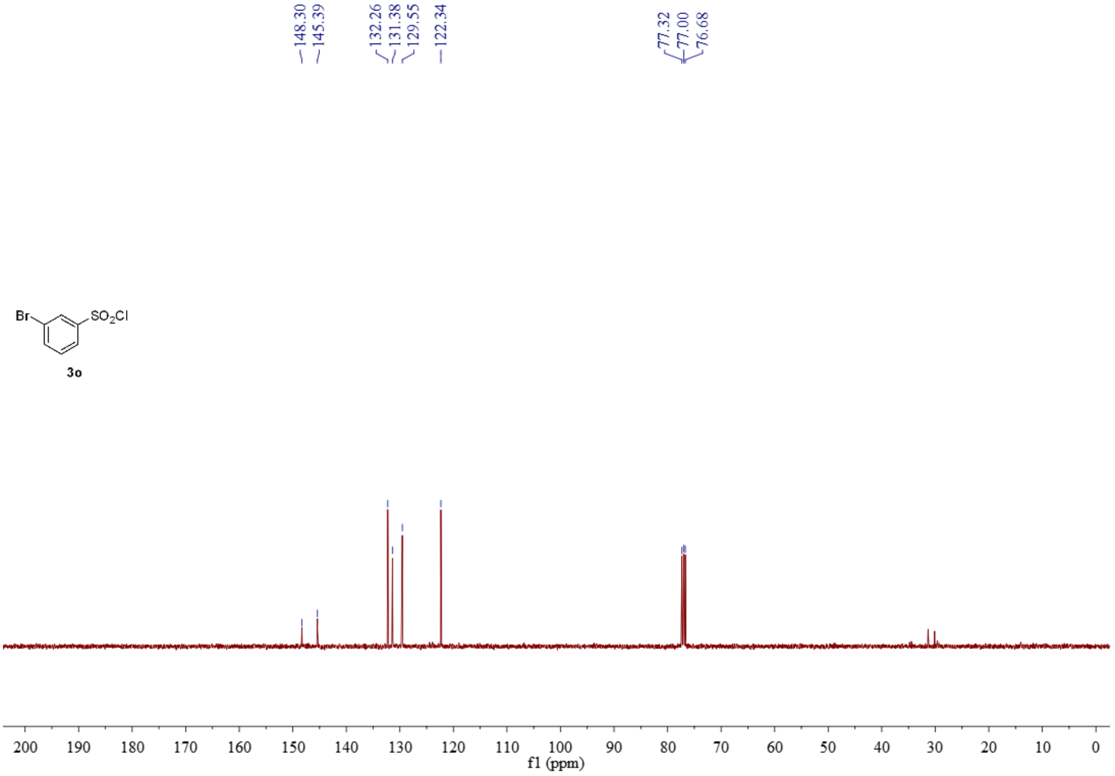
148.30
145.39
132.26
131.38
129.55
122.34
77.32
77.00
76.68
Br
SO2Cl
3o
200 190 180 170 160 150 140 130 120 110 100 90 80 70 60 50 40 30 20 10 0
f1 (ppm)

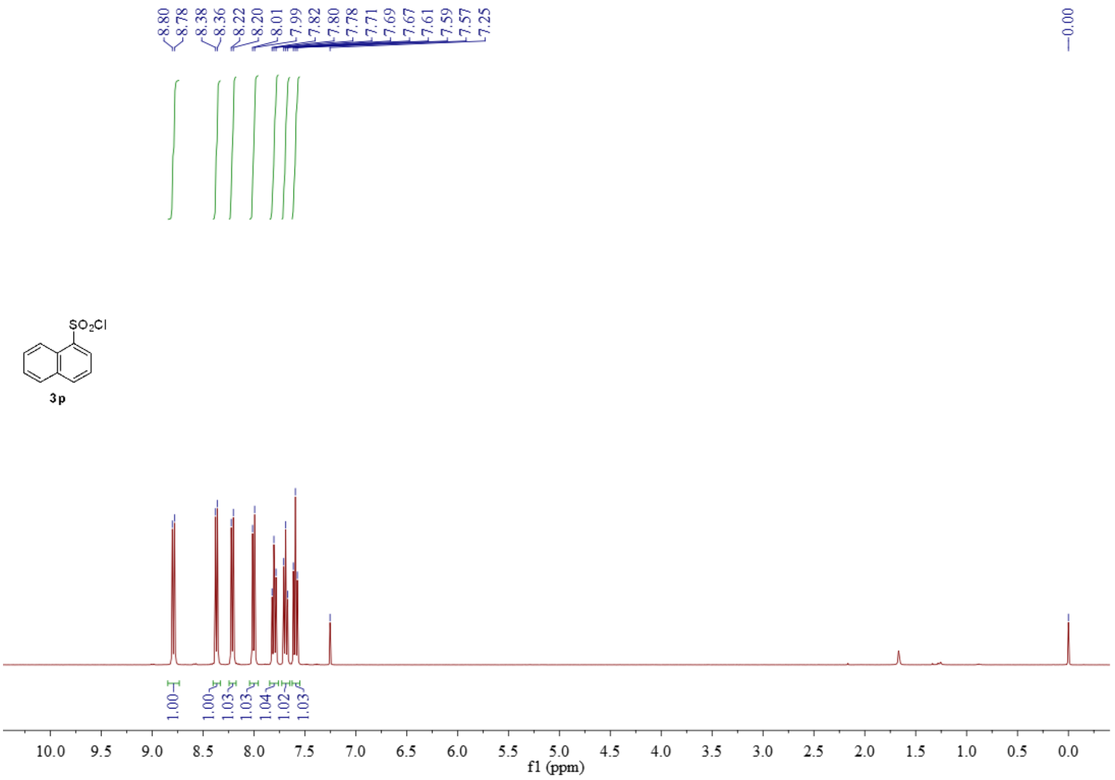
8.80
8.78
8.38
8.36
8.22
8.20
8.01
7.99
7.82
7.80
7.78
7.71
7.69
7.67
7.61
7.59
7.57
7.25
0.00
SO2Cl
3p
1.00
1.00
1.03
1.03
1.04
1.02
1.03
10.0 9.5 9.0 8.5 8.0 7.5 7.0 6.5 6.0 5.5 5.0 4.5 4.0 3.5 3.0 2.5 2.0 1.5 1.0 0.5 0.0
f1 (ppm)

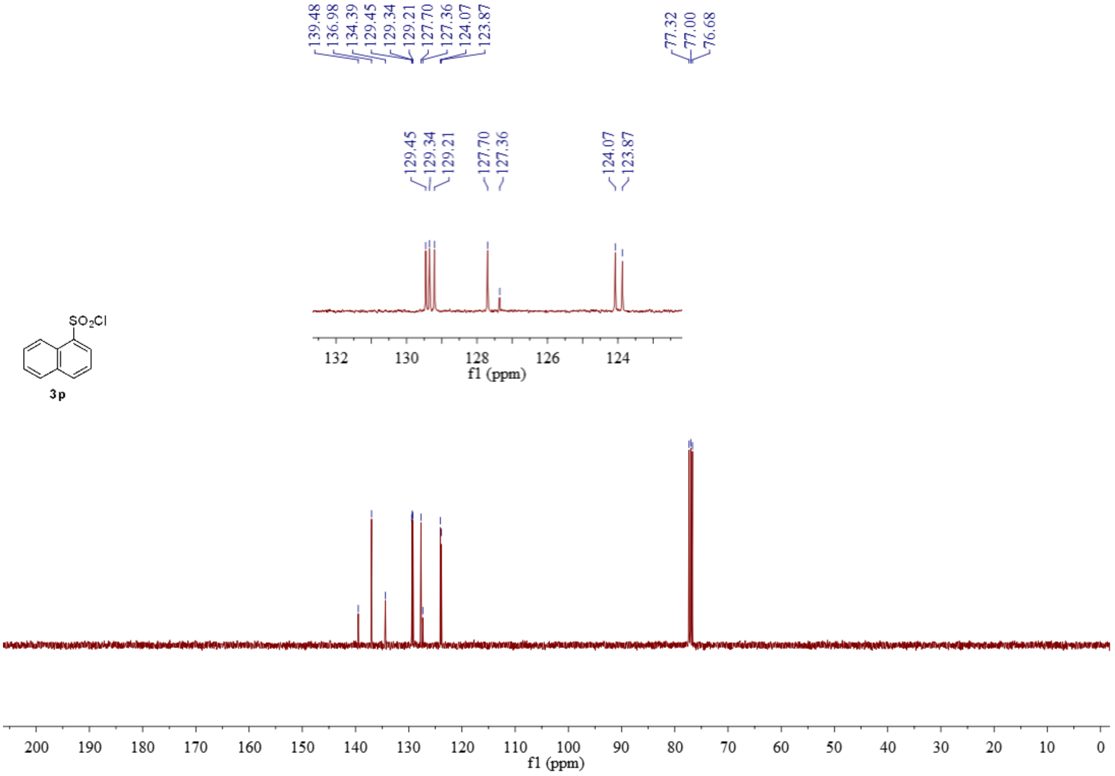
139.48
136.98
134.39
129.45
129.34
129.21
127.70
127.36
124.07
123.87
77.32
77.00
76.68
129.45
129.34
129.21
127.70
127.36
124.07
123.87
132 130 128 126 124
f1 (ppm)
SO2Cl
3p
200 190 180 170 160 150 140 130 120 110 100 90 80 70 60 50 40 30 20 10 0
f1 (ppm)

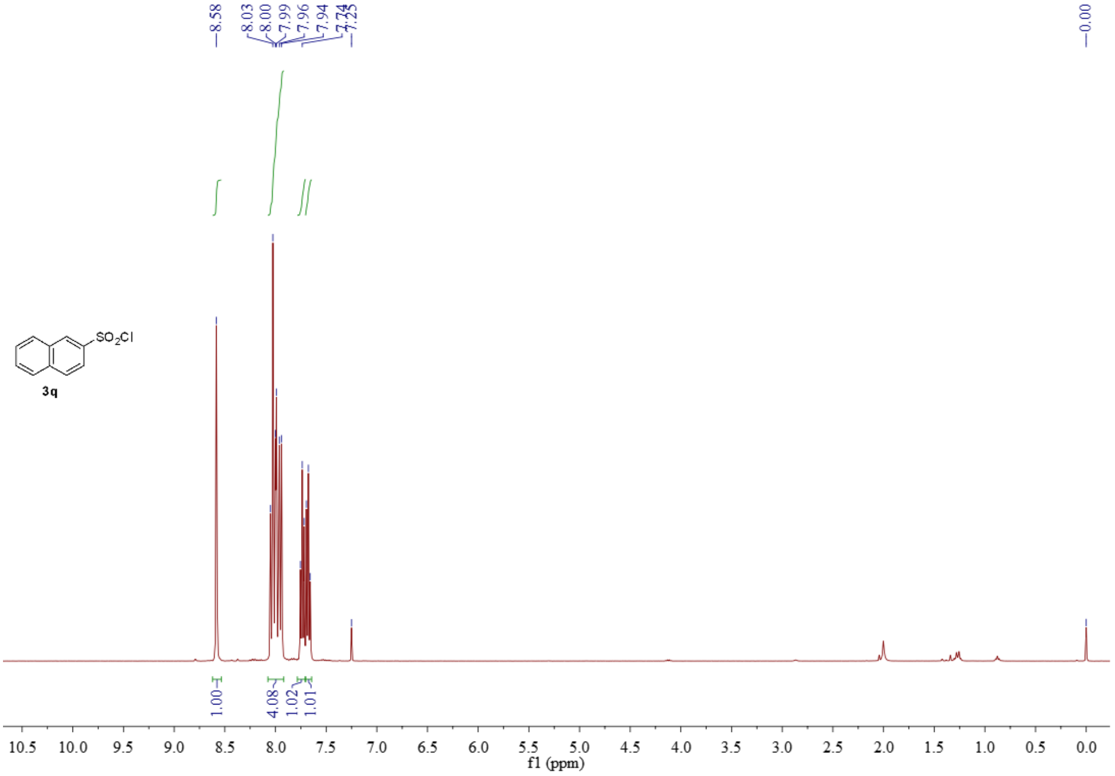
8.58
8.03
8.00
7.99
7.96
7.94
7.74
7.25
0.00
SO2Cl
3q
1.00
4.08
1.02
1.01
f1 (ppm)

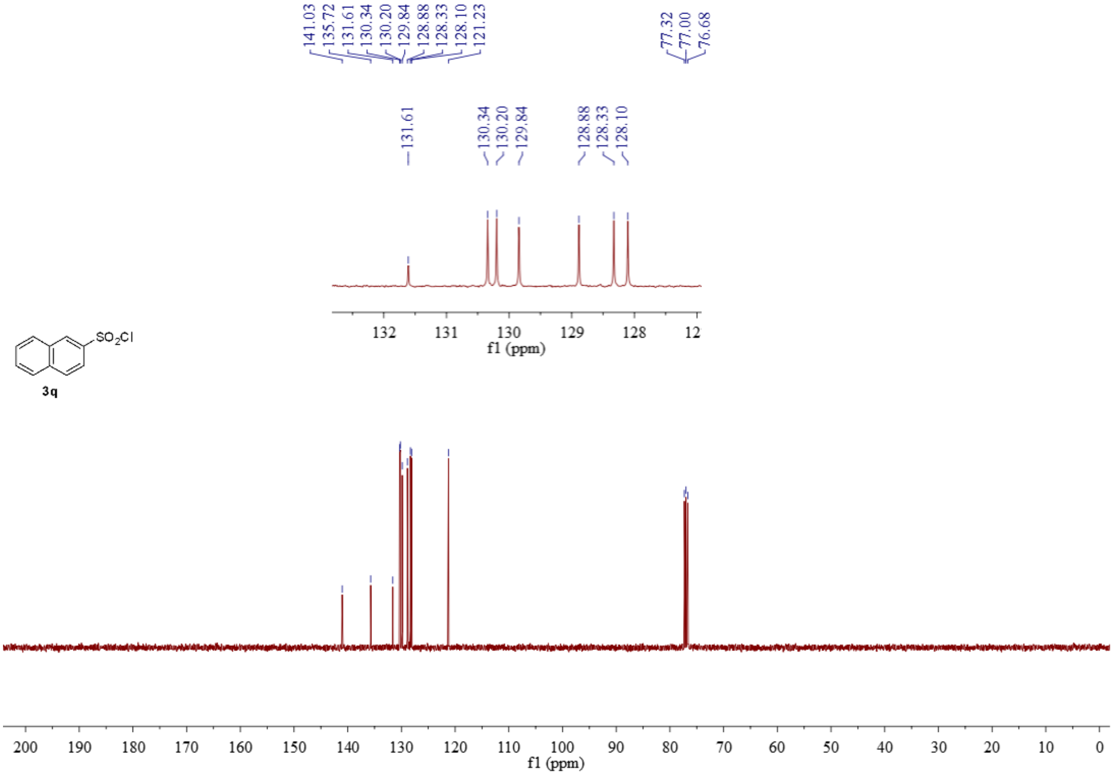
141.03
135.72
131.61
130.34
130.20
129.84
128.88
128.33
128.10
121.23
77.32
77.00
76.68
f1 (ppm)
SO2Cl
3q

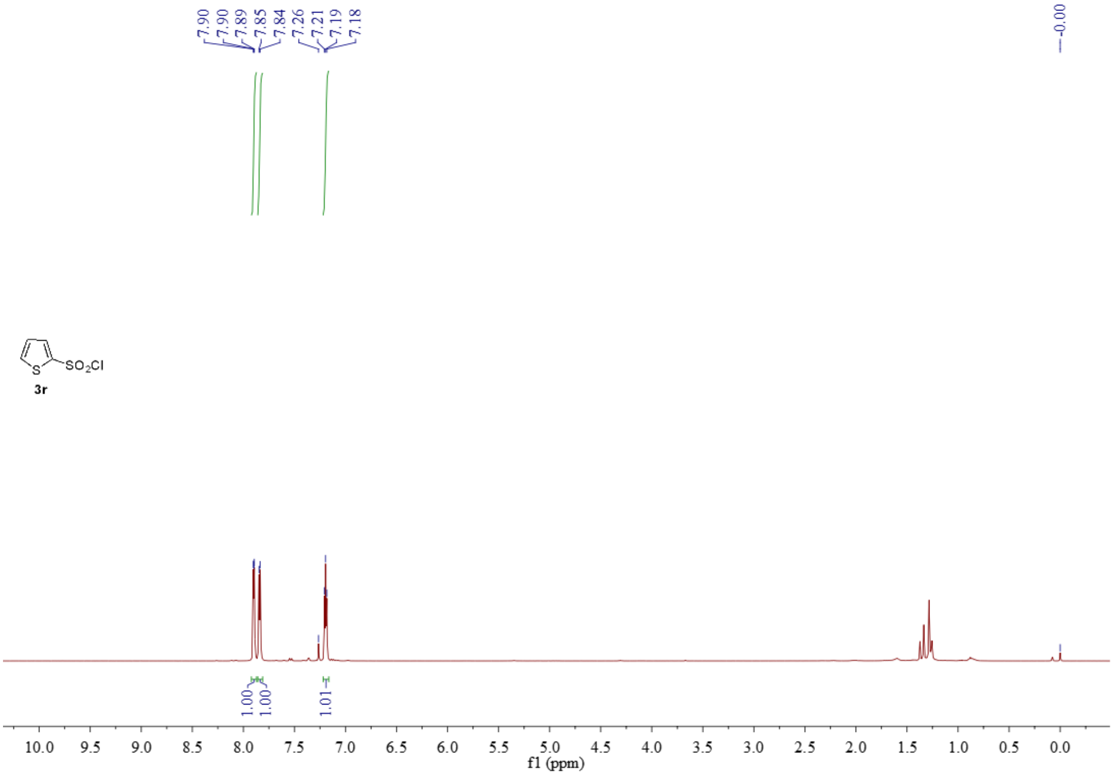
7.90
7.90
7.89
7.85
7.84
7.26
7.21
7.19
7.18
-0.00
SO_2Cl
S
3r
1.00
1.00
1.01
10.0 9.5 9.0 8.5 8.0 7.5 7.0 6.5 6.0 5.5 5.0 4.5 4.0 3.5 3.0 2.5 2.0 1.5 1.0 0.5 0.0
f1 (ppm)

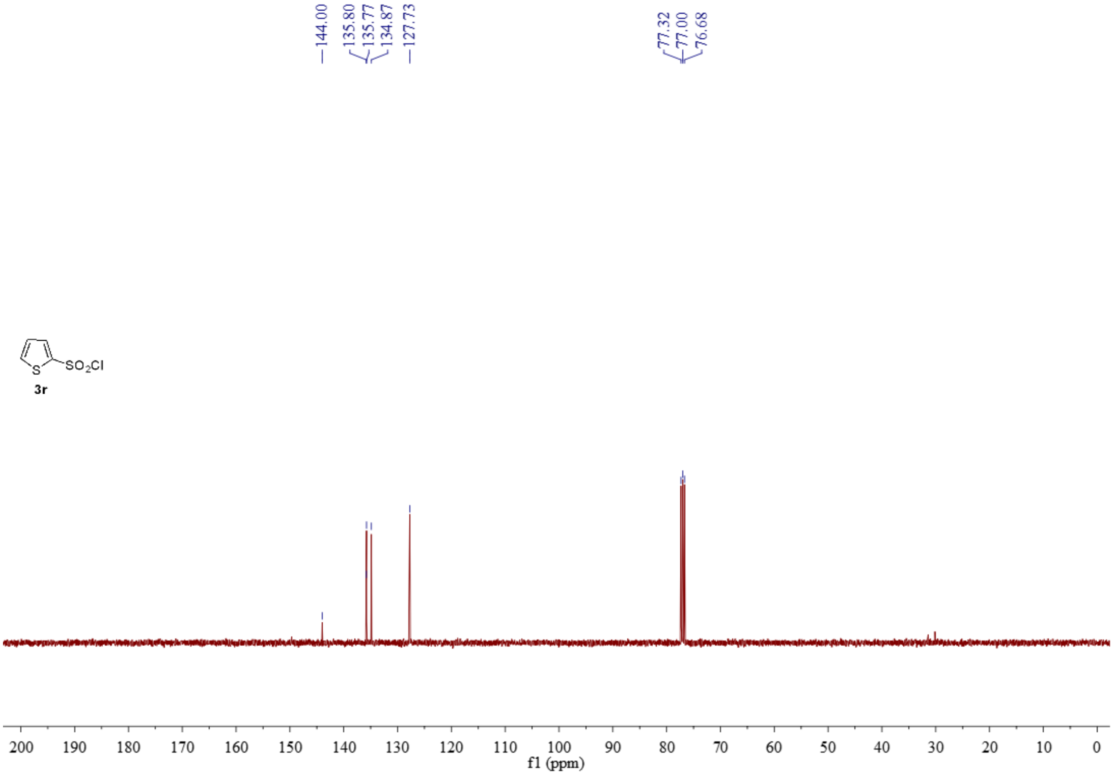
144.00
135.80
135.77
134.87
127.73
77.32
77.00
76.68
SO_2Cl
S
3r
200 190 180 170 160 150 140 130 120 110 100 90 80 70 60 50 40 30 20 10 0
f1 (ppm)

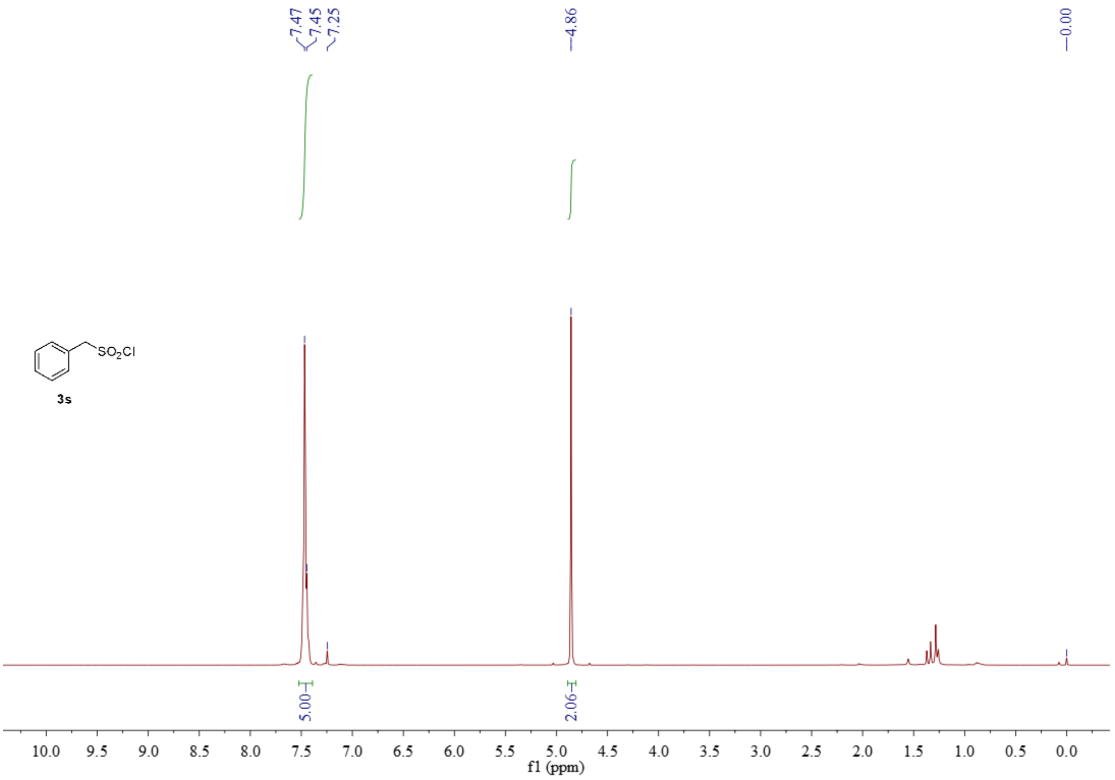
7.47
7.45
7.25
4.86
0.00
SO2Cl
3s
5.00
2.06
10.0 9.5 9.0 8.5 8.0 7.5 7.0 6.5 6.0 5.5 5.0 4.5 4.0 3.5 3.0 2.5 2.0 1.5 1.0 0.5 0.0
f1 (ppm)

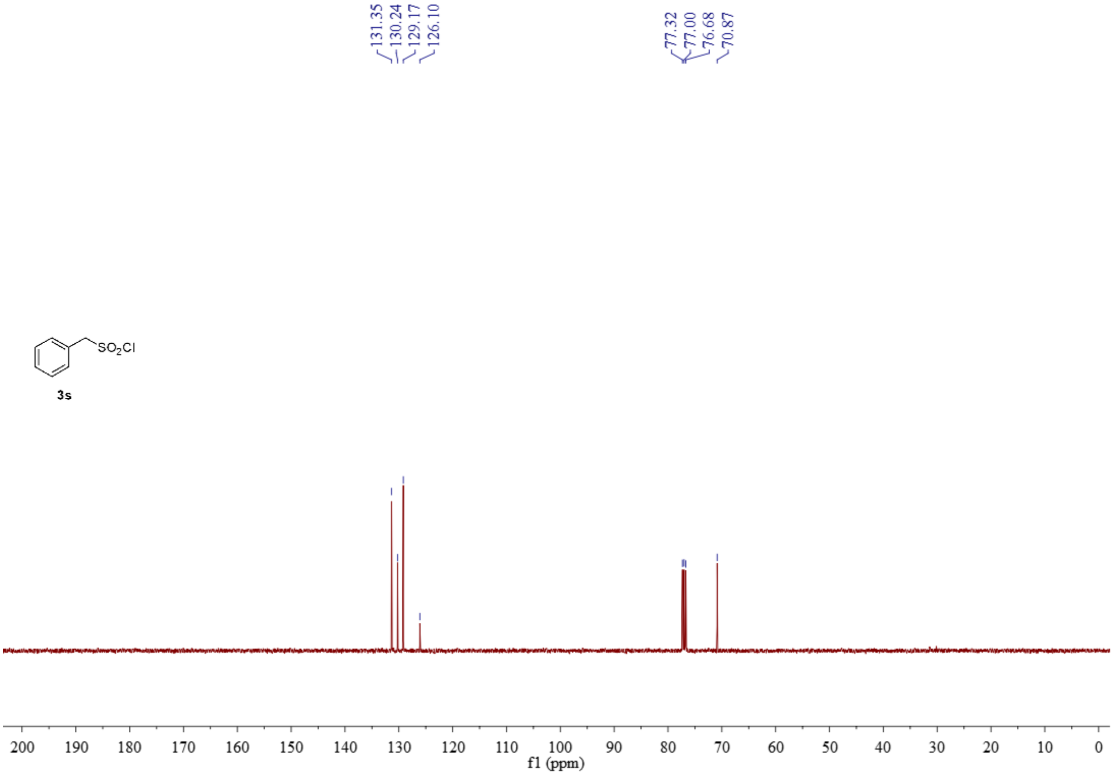
131.35
130.24
129.17
126.10
77.32
77.00
76.68
70.87
SO2Cl
3s
200 190 180 170 160 150 140 130 120 110 100 90 80 70 60 50 40 30 20 10 0
f1 (ppm)

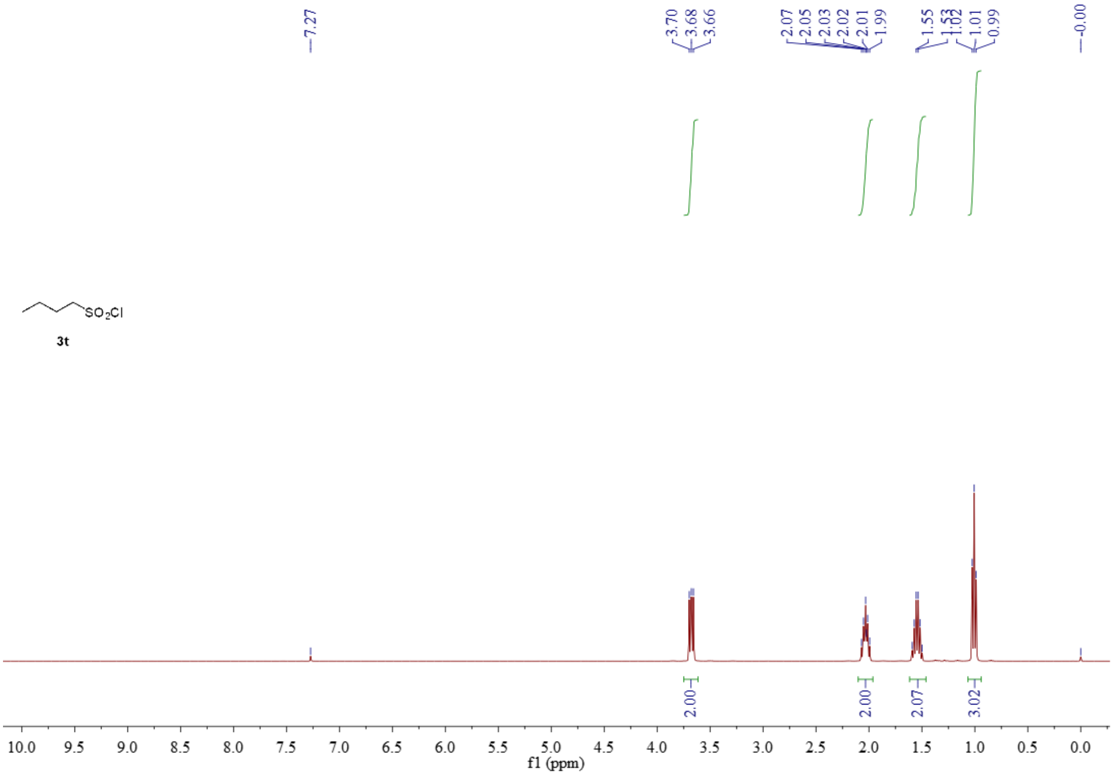
7.27
3.70
3.68
3.66
2.07
2.05
2.03
2.02
2.01
1.99
1.55
1.53
1.02
1.01
0.99
0.00
SO2Cl
3t
2.00
2.00
2.07
3.02
10.0 9.5 9.0 8.5 8.0 7.5 7.0 6.5 6.0 5.5 5.0 4.5 4.0 3.5 3.0 2.5 2.0 1.5 1.0 0.5 0.0
f1 (ppm)

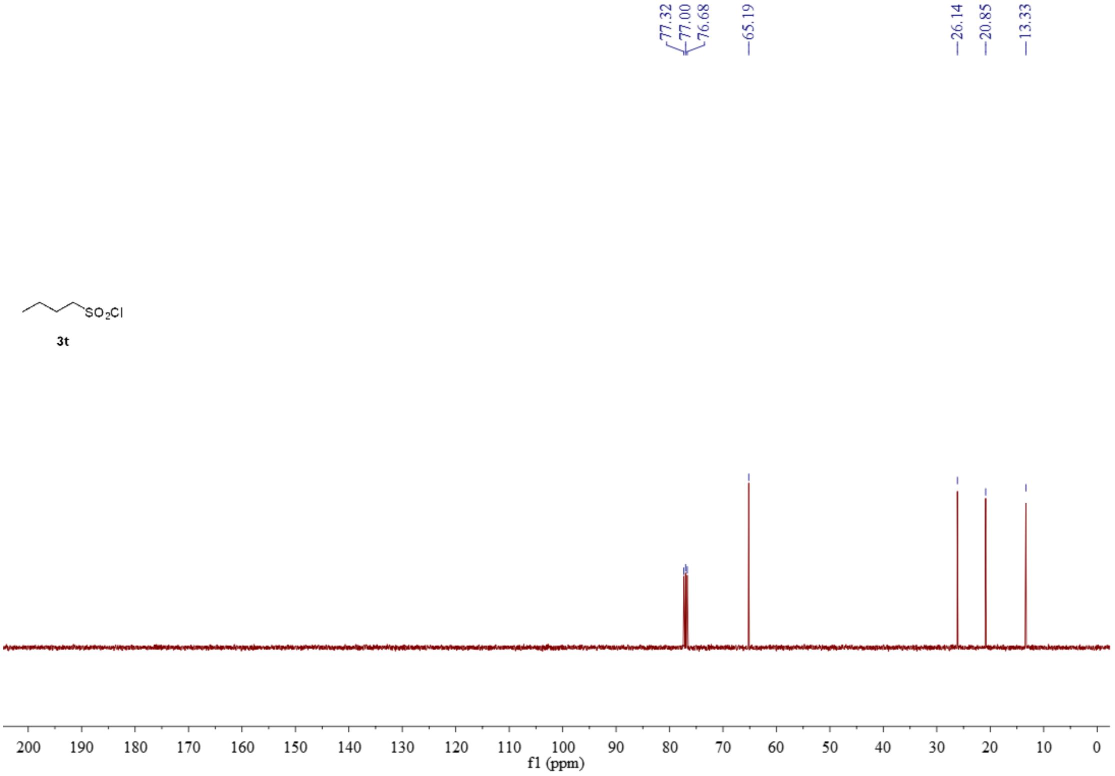
77.32
77.00
76.68
65.19
26.14
20.85
13.33
SO2Cl
3t
200 190 180 170 160 150 140 130 120 110 100 90 80 70 60 50 40 30 20 10 0
f1 (ppm)

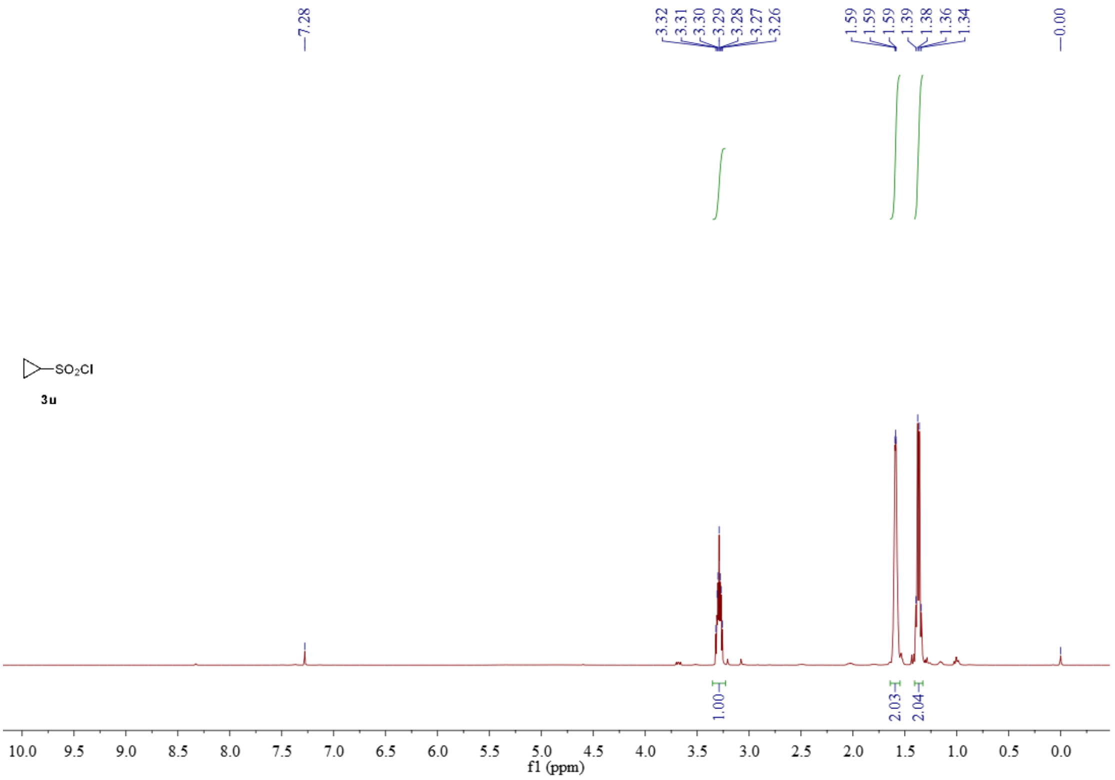
7.28
3.32
3.31
3.30
3.29
3.28
3.27
3.26
1.59
1.59
1.59
1.39
1.38
1.36
1.34
0.00
SO2Cl
3u
1.00
2.03
2.04
10.0 9.5 9.0 8.5 8.0 7.5 7.0 6.5 6.0 5.5 5.0 4.5 4.0 3.5 3.0 2.5 2.0 1.5 1.0 0.5 0.0
f1 (ppm)

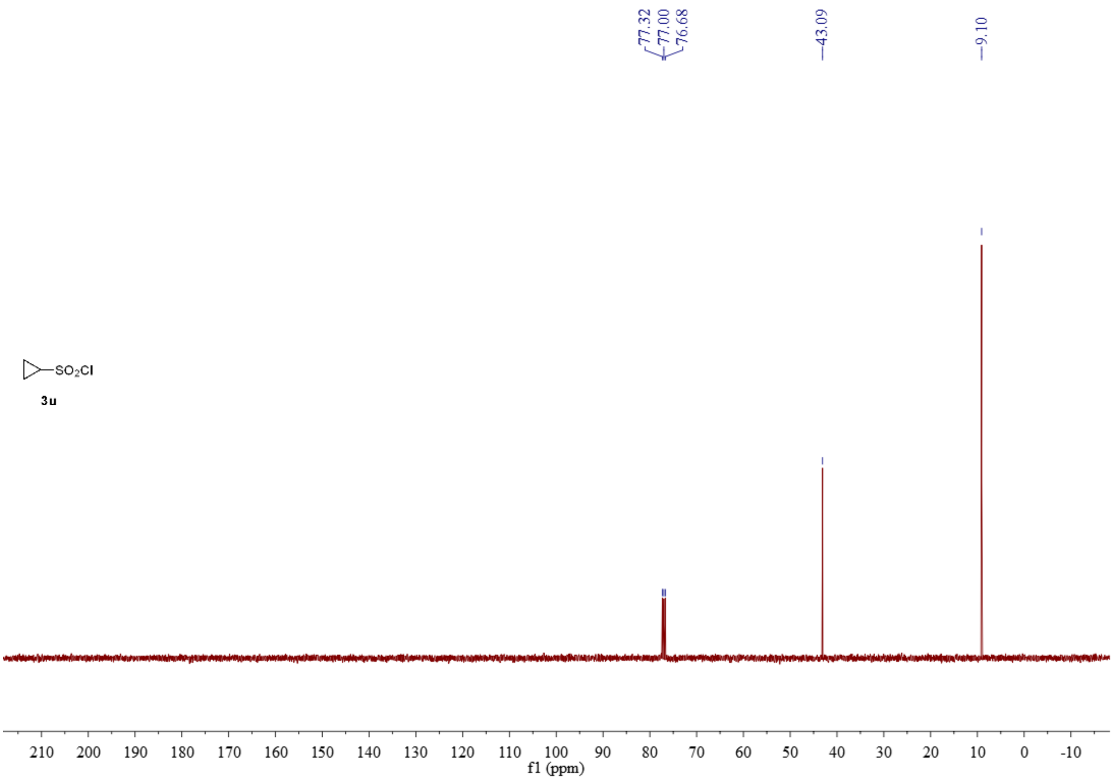
77.32
77.00
76.68
43.09
9.10
SO2Cl
3u
210 200 190 180 170 160 150 140 130 120 110 100 90 80 70 60 50 40 30 20 10 0 -10
f1 (ppm)

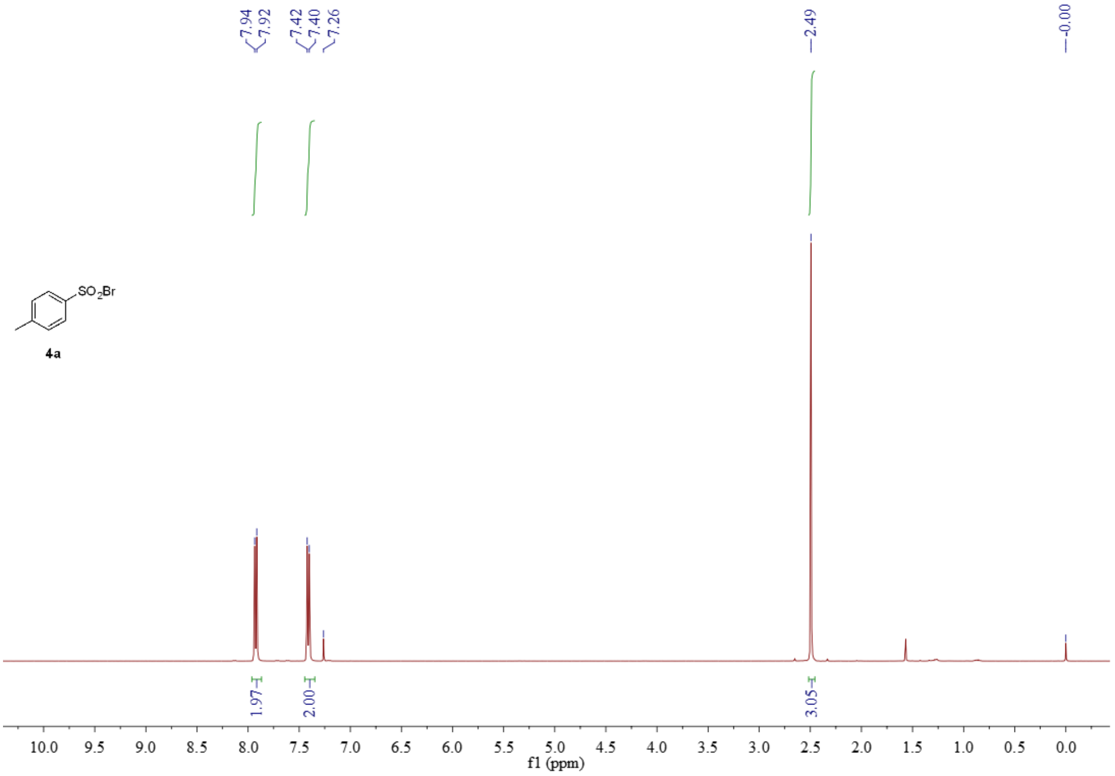
7.94
7.92
7.42
7.40
7.26
2.49
-0.00
SO2Br
4a
1.97
2.00
3.05
10.0 9.5 9.0 8.5 8.0 7.5 7.0 6.5 6.0 5.5 5.0 4.5 4.0 3.5 3.0 2.5 2.0 1.5 1.0 0.5 0.0
f1 (ppm)

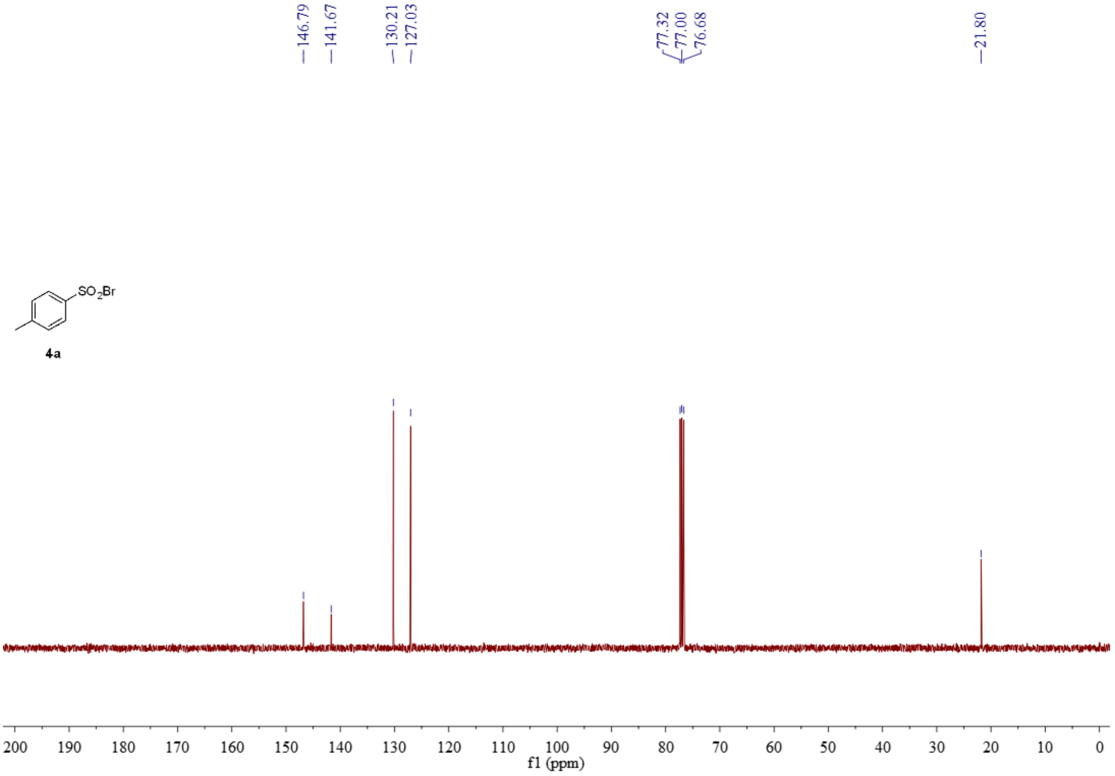
146.79
141.67
130.21
127.03
77.32
77.00
76.68
21.80
SO2Br
4a
200 190 180 170 160 150 140 130 120 110 100 90 80 70 60 50 40 30 20 10 0
f1 (ppm)

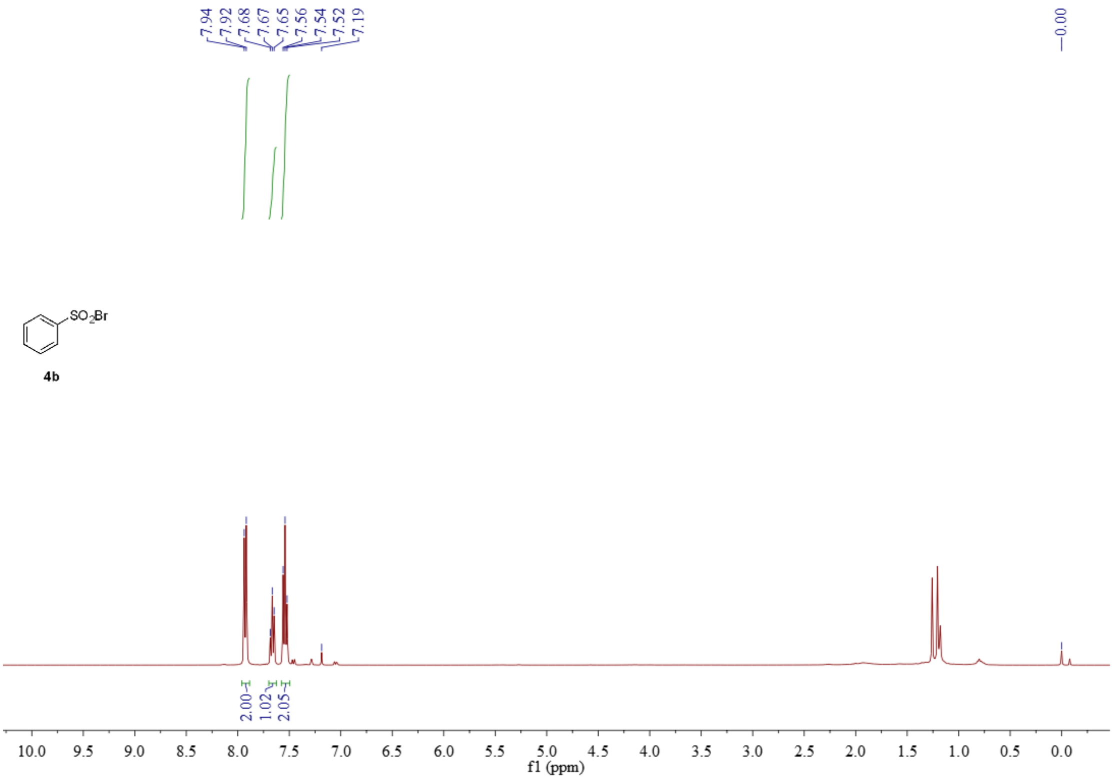
7.94
7.92
7.68
7.67
7.65
7.56
7.54
7.52
7.19
0.00
SO2Br
4b
2.00
1.02
2.05
10.0 9.5 9.0 8.5 8.0 7.5 7.0 6.5 6.0 5.5 5.0 4.5 4.0 3.5 3.0 2.5 2.0 1.5 1.0 0.5 0.0
f1 (ppm)

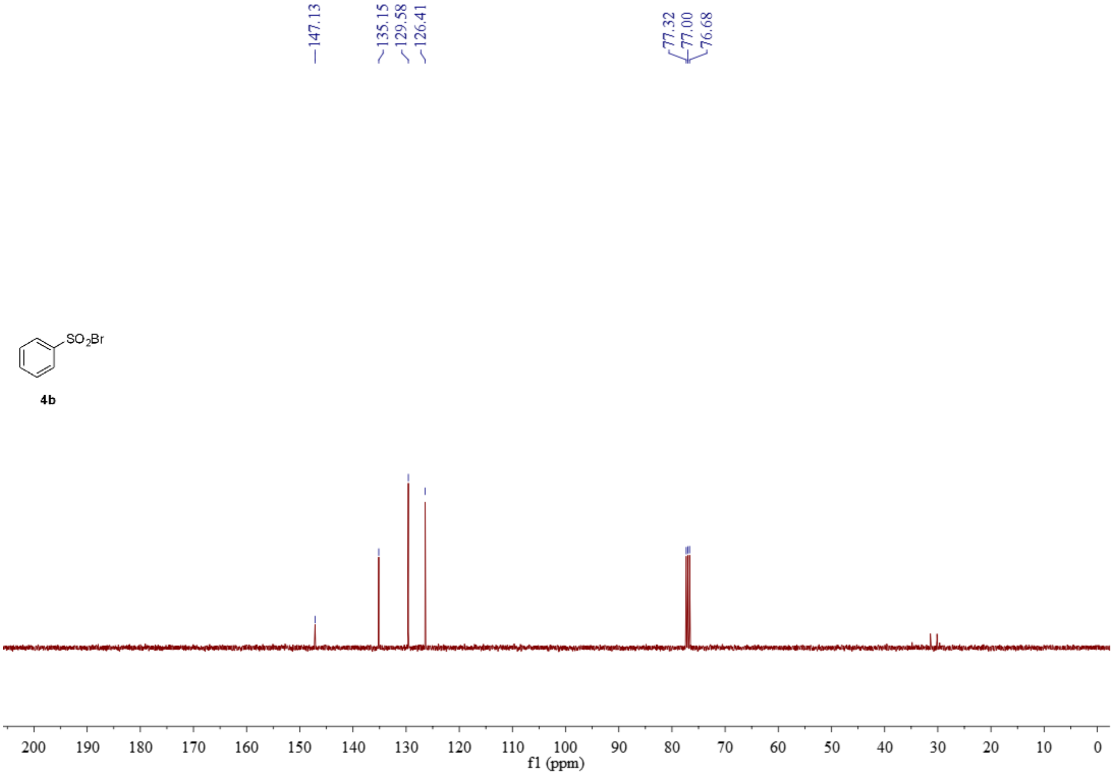
147.13
135.15
129.58
126.41
77.32
77.00
76.68
SO2Br
4b
200 190 180 170 160 150 140 130 120 110 100 90 80 70 60 50 40 30 20 10 0
f1 (ppm)

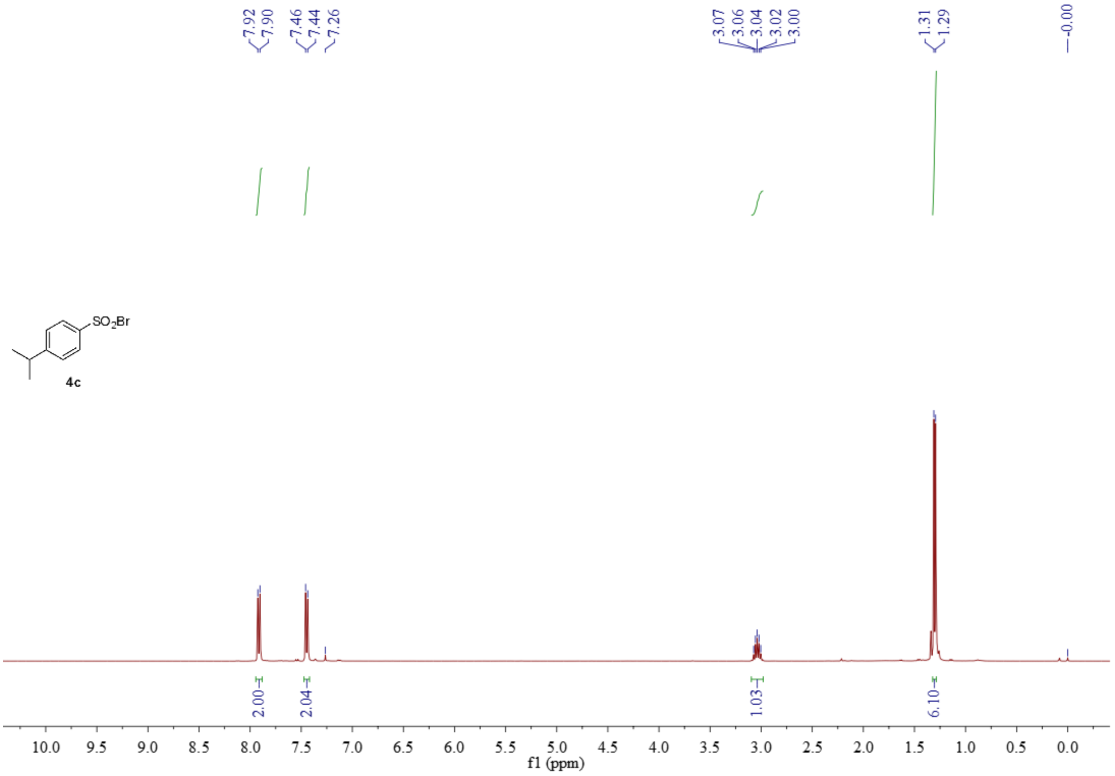
7.92
7.90
7.46
7.44
7.26
3.07
3.06
3.04
3.02
3.00
1.31
1.29
0.00
SO2Br
4c
2.00
2.04
1.03
6.10
10.0 9.5 9.0 8.5 8.0 7.5 7.0 6.5 6.0 5.5 5.0 4.5 4.0 3.5 3.0 2.5 2.0 1.5 1.0 0.5 0.0
f1 (ppm)

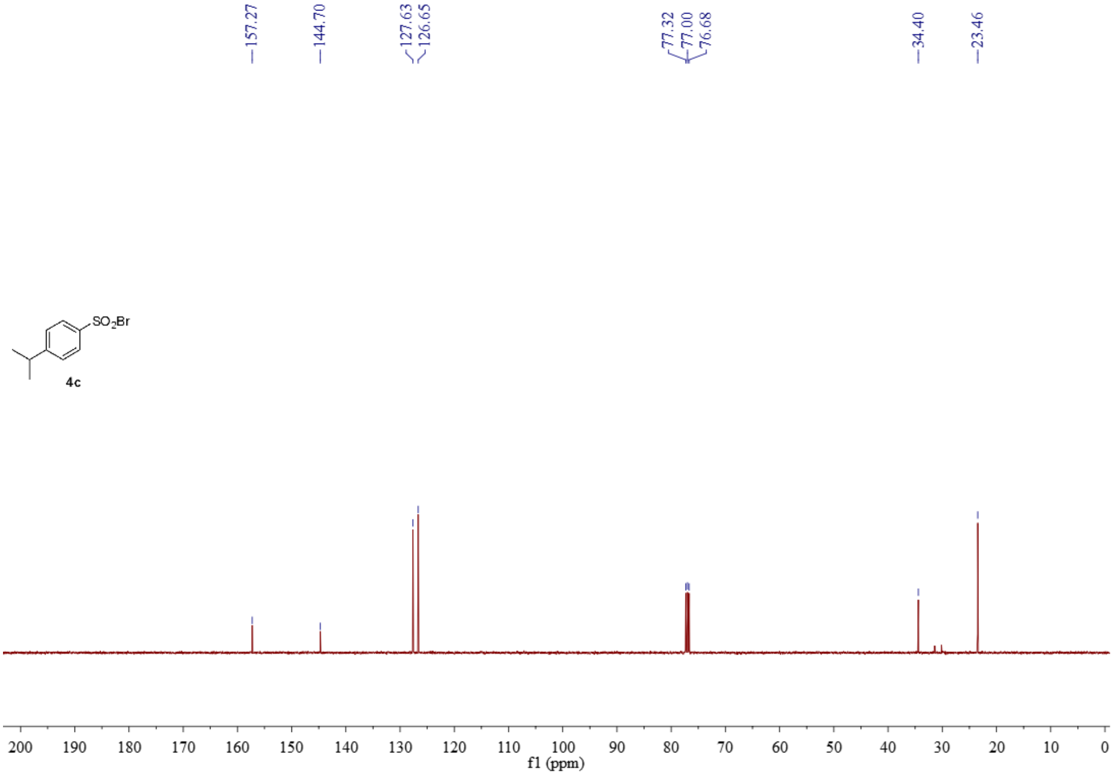
157.27
144.70
127.63
126.65
77.32
77.00
76.68
34.40
23.46
SO2Br
4c
200 190 180 170 160 150 140 130 120 110 100 90 80 70 60 50 40 30 20 10 0
f1 (ppm)

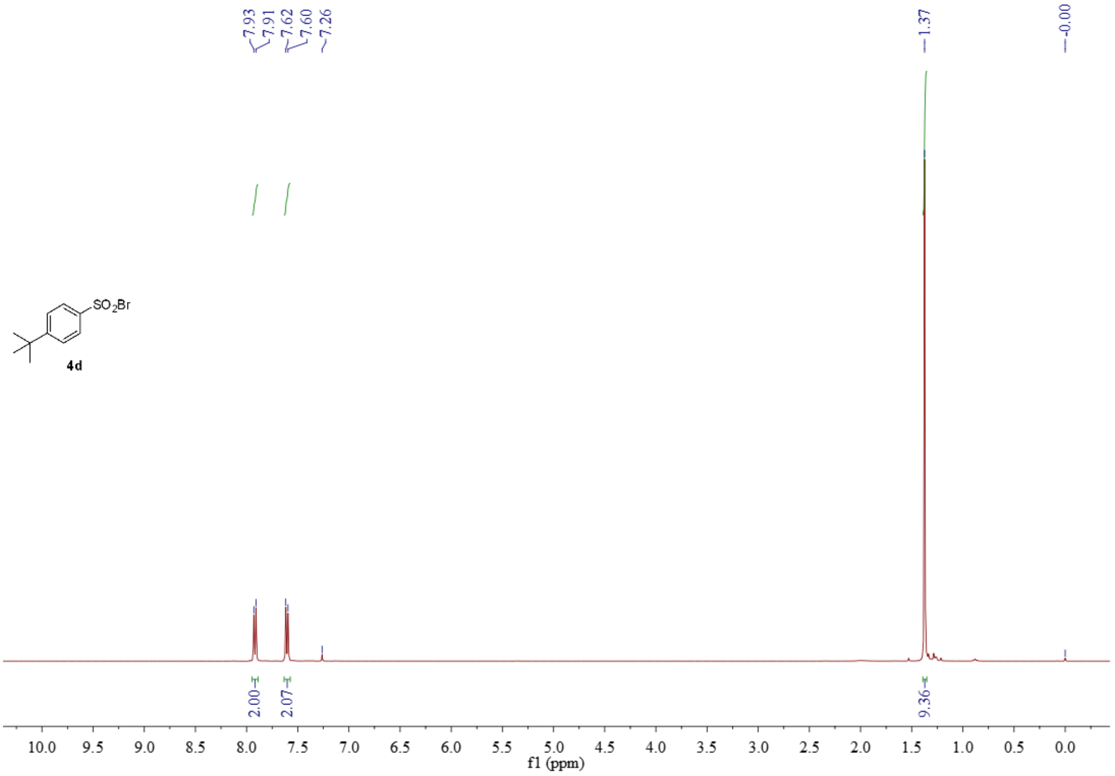
7.93
7.91
7.62
7.60
7.26
1.37
0.00
SO2Br
4d
2.00
2.07
9.36
10.0 9.5 9.0 8.5 8.0 7.5 7.0 6.5 6.0 5.5 5.0 4.5 4.0 3.5 3.0 2.5 2.0 1.5 1.0 0.5 0.0
f1 (ppm)

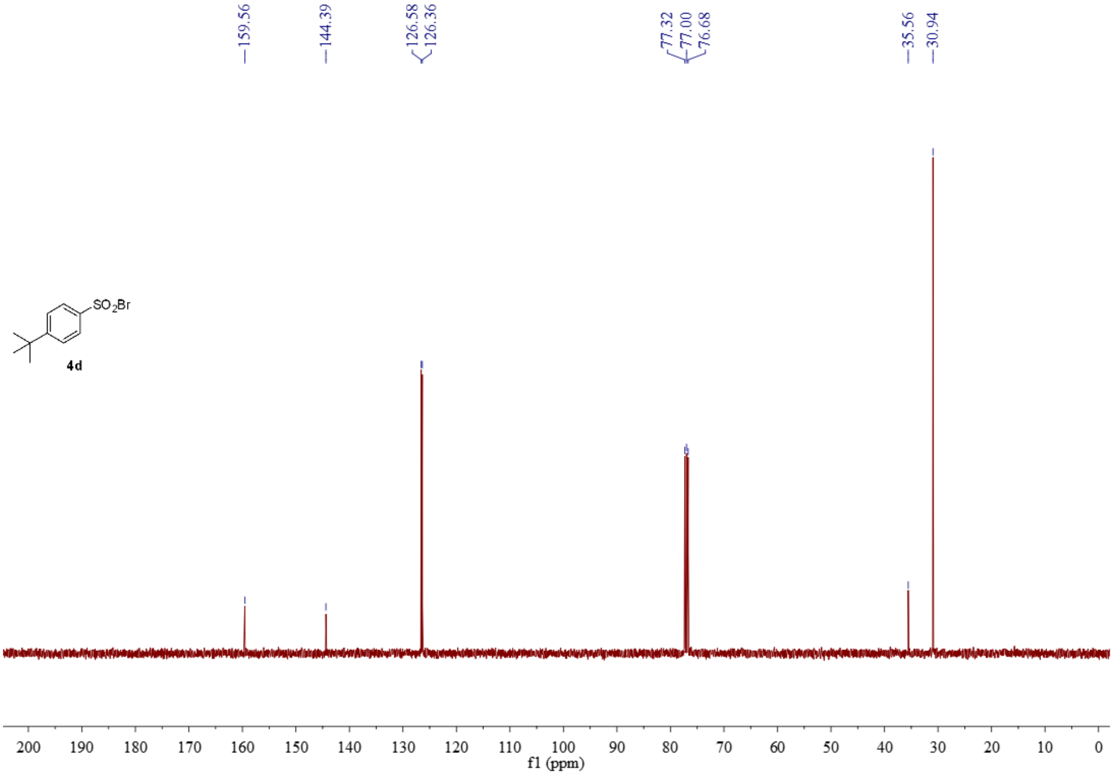
159.56
144.39
126.58
126.36
77.32
77.00
76.68
35.56
30.94
SO2Br
4d
200 190 180 170 160 150 140 130 120 110 100 90 80 70 60 50 40 30 20 10 0
f1 (ppm)

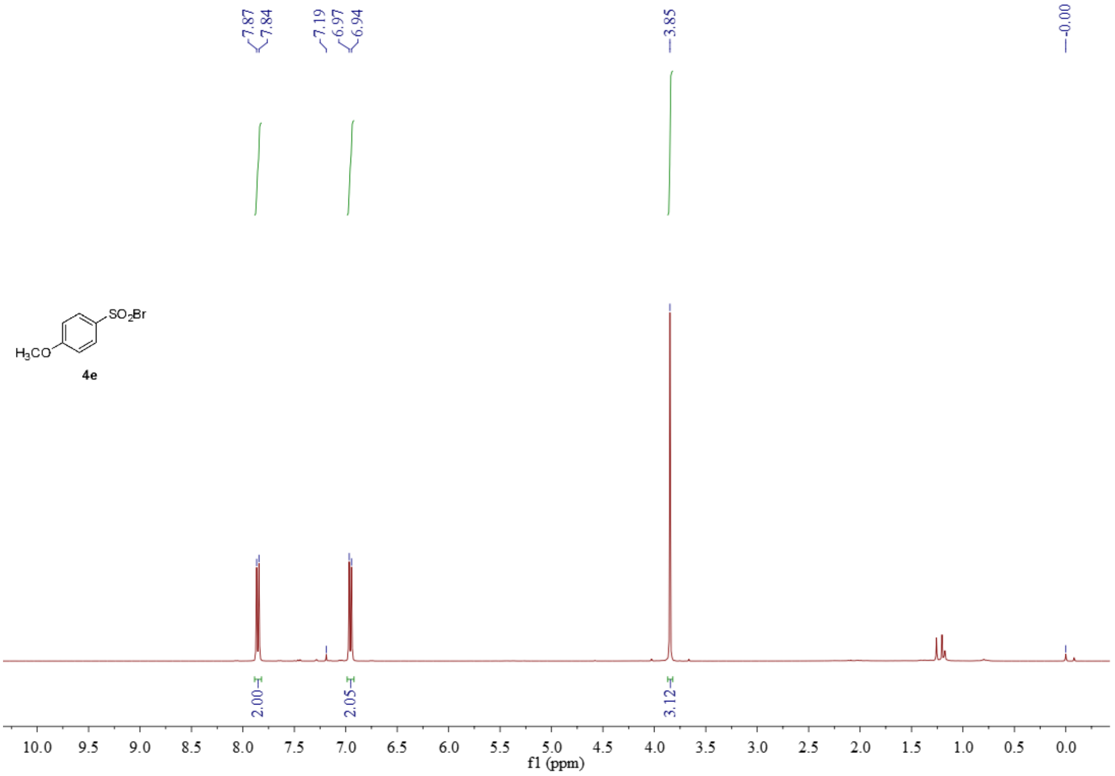
7.87
7.84
7.19
6.97
6.94
3.85
-0.00
SO2Br
H3CO
4e
2.00
2.05
3.12
10.0 9.5 9.0 8.5 8.0 7.5 7.0 6.5 6.0 5.5 5.0 4.5 4.0 3.5 3.0 2.5 2.0 1.5 1.0 0.5 0.0
f1 (ppm)

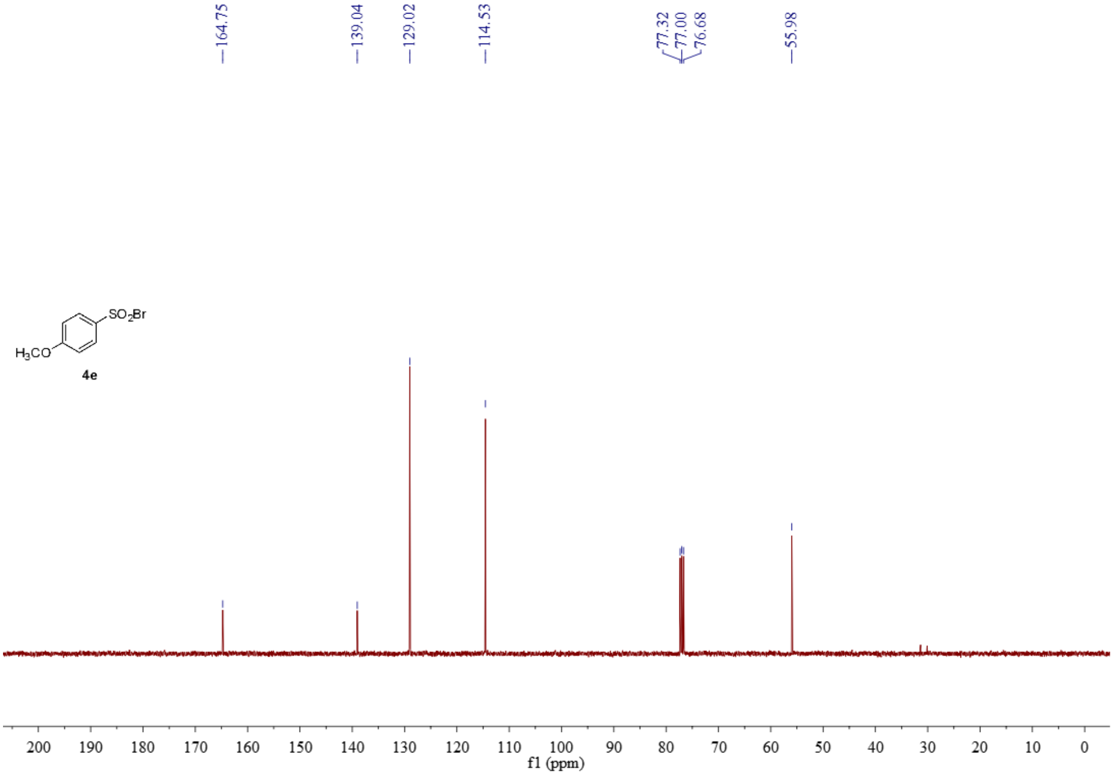
164.75
139.04
129.02
114.53
77.32
77.00
76.68
55.98
SO2Br
H3CO
4e
200 190 180 170 160 150 140 130 120 110 100 90 80 70 60 50 40 30 20 10 0
f1 (ppm)

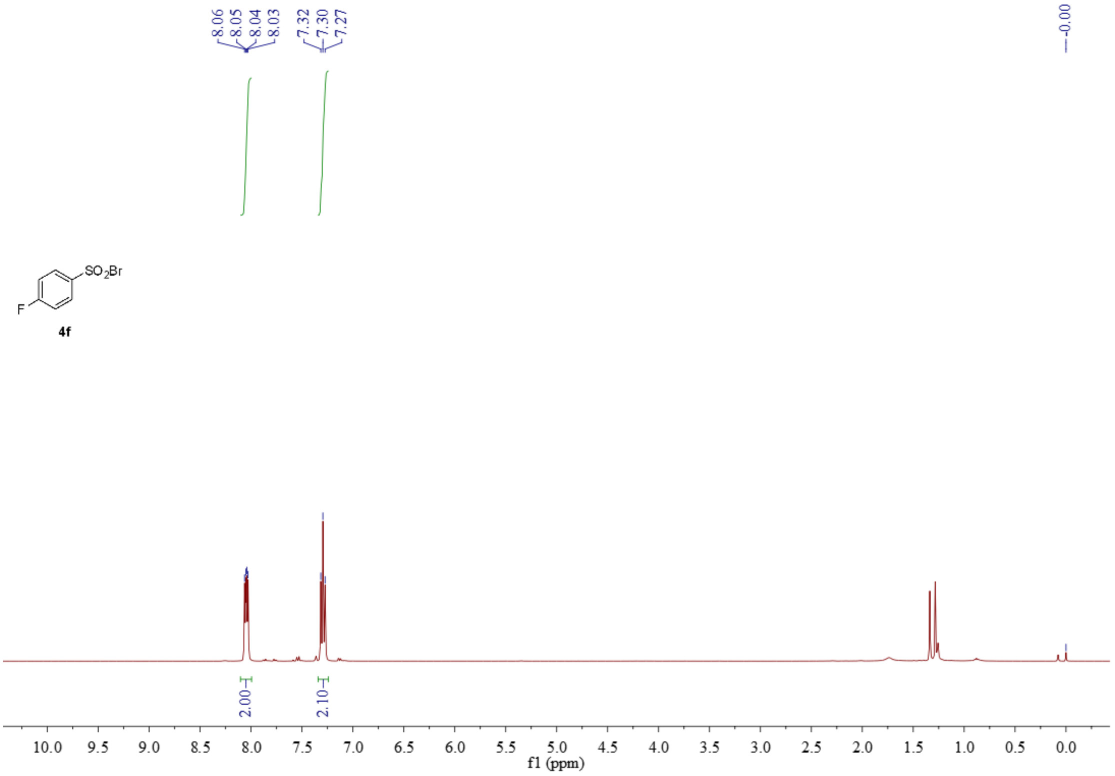
8.06
8.05
8.04
8.03
7.32
7.30
7.27
0.00
SO2Br
F
4f
2.00
2.10
10.0 9.5 9.0 8.5 8.0 7.5 7.0 6.5 6.0 5.5 5.0 4.5 4.0 3.5 3.0 2.5 2.0 1.5 1.0 0.5 0.0
f1 (ppm)

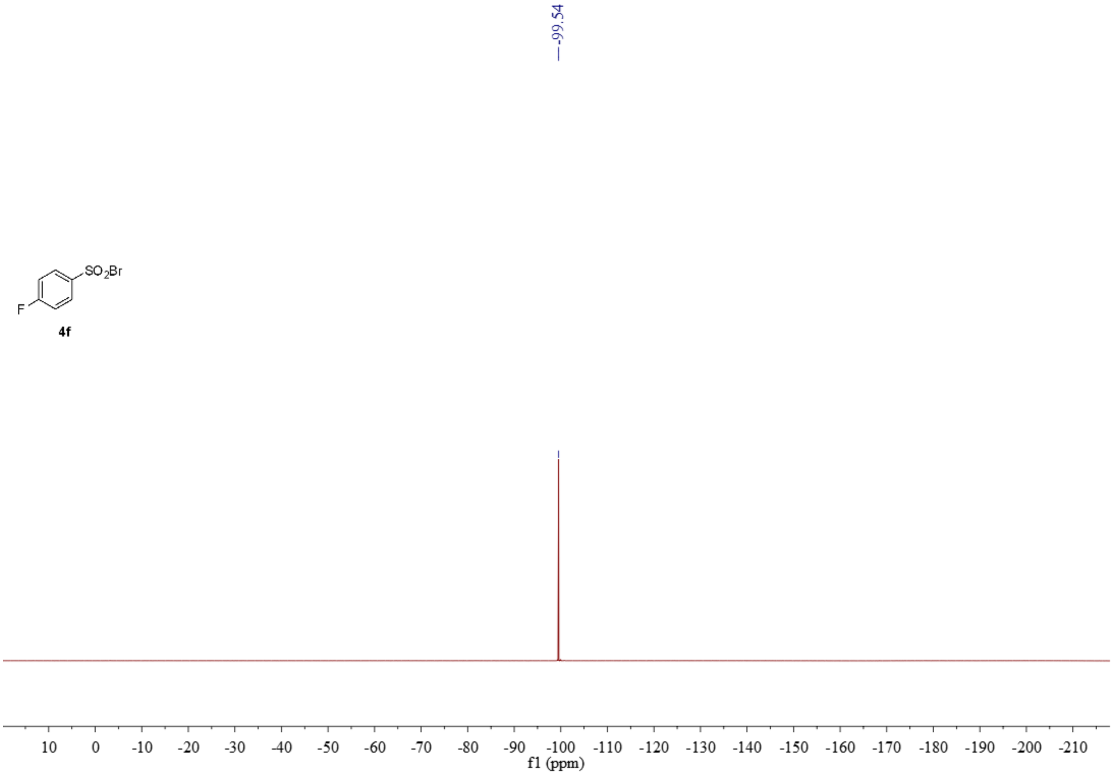
-99.54
SO2Br
F
4f
10 0 -10 -20 -30 -40 -50 -60 -70 -80 -90 -100 -110 -120 -130 -140 -150 -160 -170 -180 -190 -200 -210
f1 (ppm)

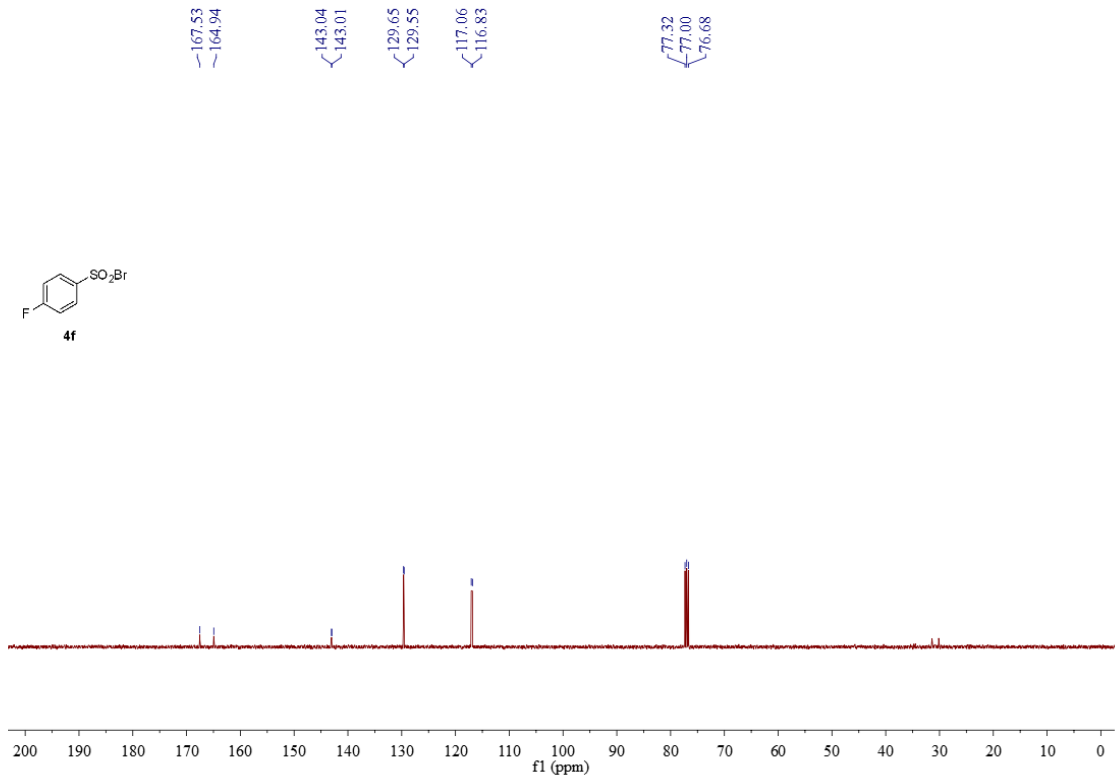

167.53
164.94
143.04
143.01
129.65
129.55
117.06
116.83
77.32
77.00
76.68
SO2Br
F
4f
200 190 180 170 160 150 140 130 120 110 100 90 80 70 60 50 40 30 20 10 0
f1 (ppm)

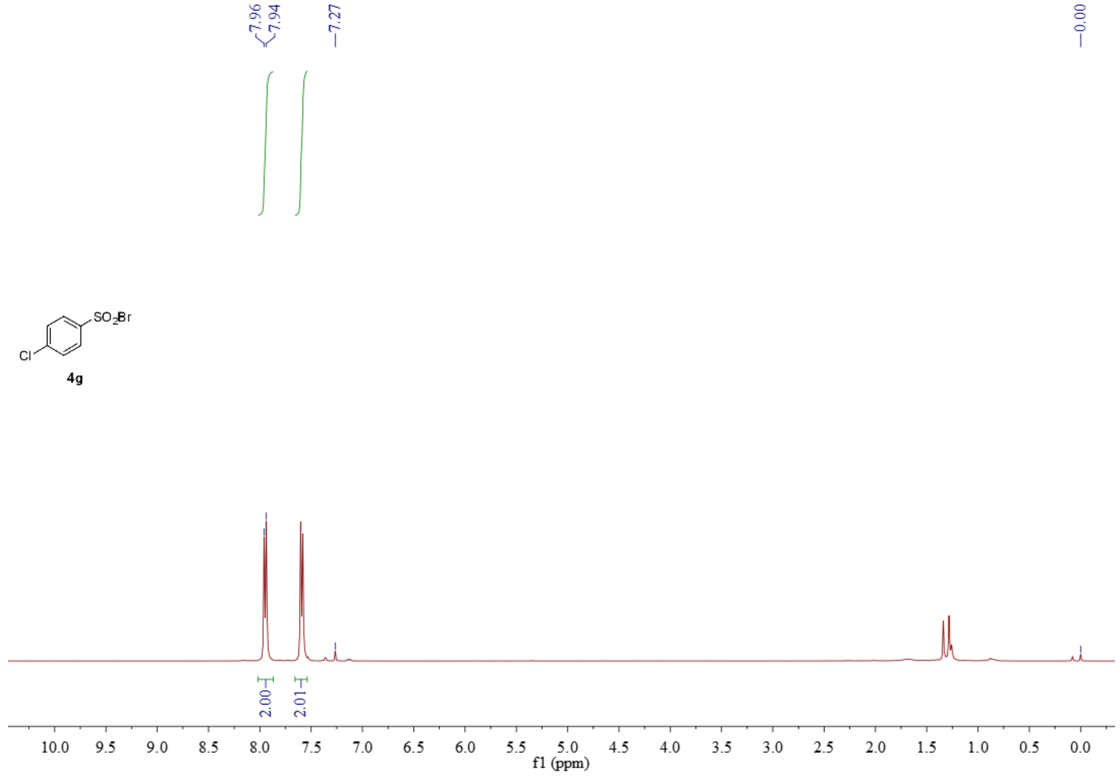

7.96
7.94
7.27
0.00
SO2Br
Cl
4g
2.00
2.01
10.0 9.5 9.0 8.5 8.0 7.5 7.0 6.5 6.0 5.5 5.0 4.5 4.0 3.5 3.0 2.5 2.0 1.5 1.0 0.5 0.0
f1 (ppm)

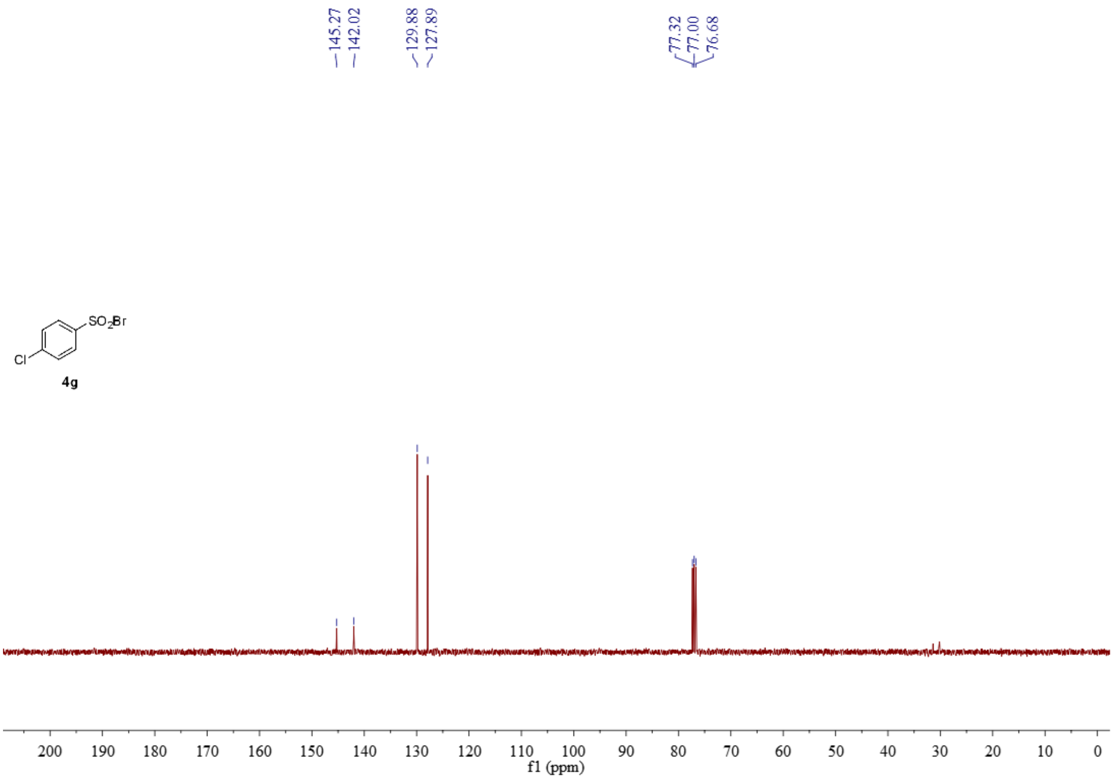
145.27
142.02
129.88
127.89
77.32
77.00
76.68
SO2Br
Cl
4g
200 190 180 170 160 150 140 130 120 110 100 90 80 70 60 50 40 30 20 10 0
f1 (ppm)

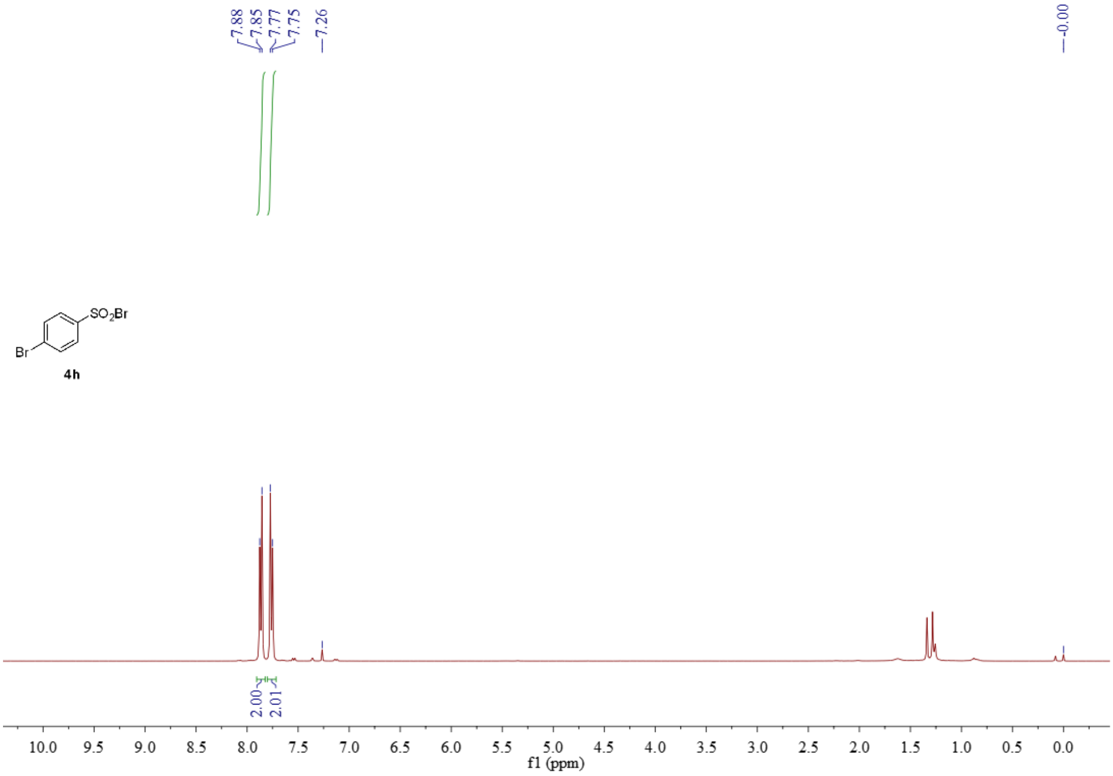
7.88
7.85
7.77
7.75
7.26
0.00
SO2Br
Br
4h
2.00
2.01
10.0 9.5 9.0 8.5 8.0 7.5 7.0 6.5 6.0 5.5 5.0 4.5 4.0 3.5 3.0 2.5 2.0 1.5 1.0 0.5 0.0
f1 (ppm)

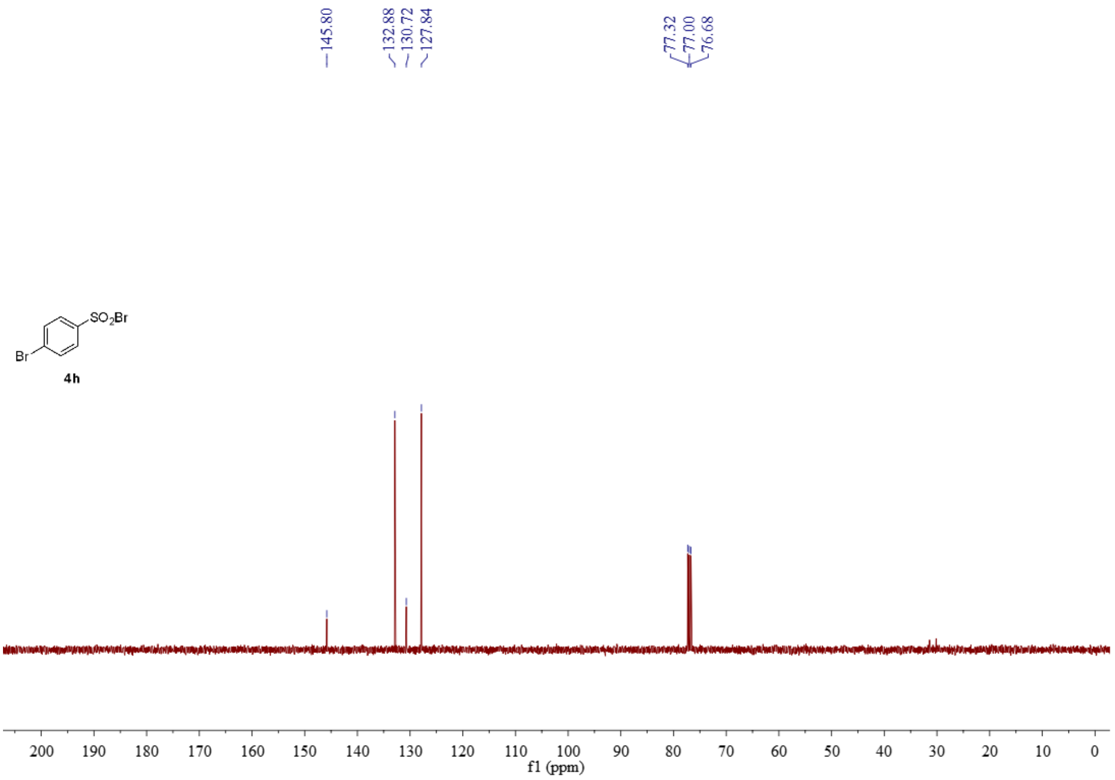
145.80
132.88
130.72
127.84
77.32
77.00
76.68
SO2Br
Br
4h
200 190 180 170 160 150 140 130 120 110 100 90 80 70 60 50 40 30 20 10 0
f1 (ppm)

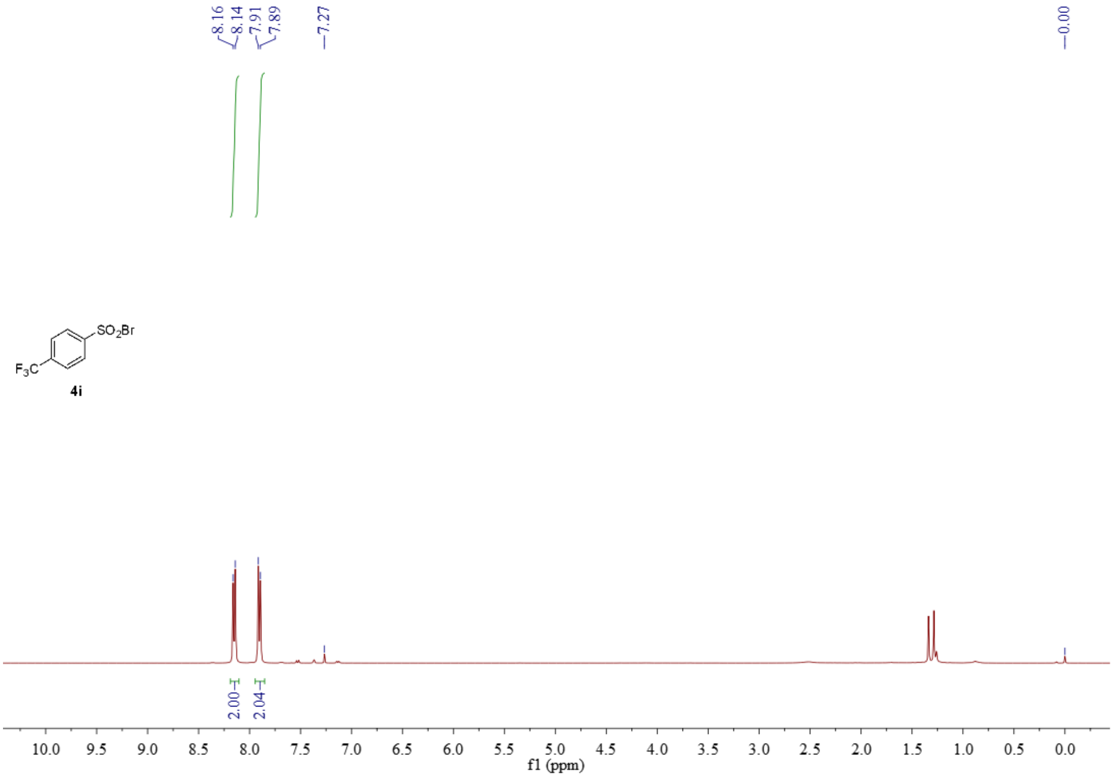
8.16
8.14
7.91
7.89
7.27
0.00
SO2Br
F3C
4i
2.00
2.04
10.0 9.5 9.0 8.5 8.0 7.5 7.0 6.5 6.0 5.5 5.0 4.5 4.0 3.5 3.0 2.5 2.0 1.5 1.0 0.5 0.0
f1 (ppm)

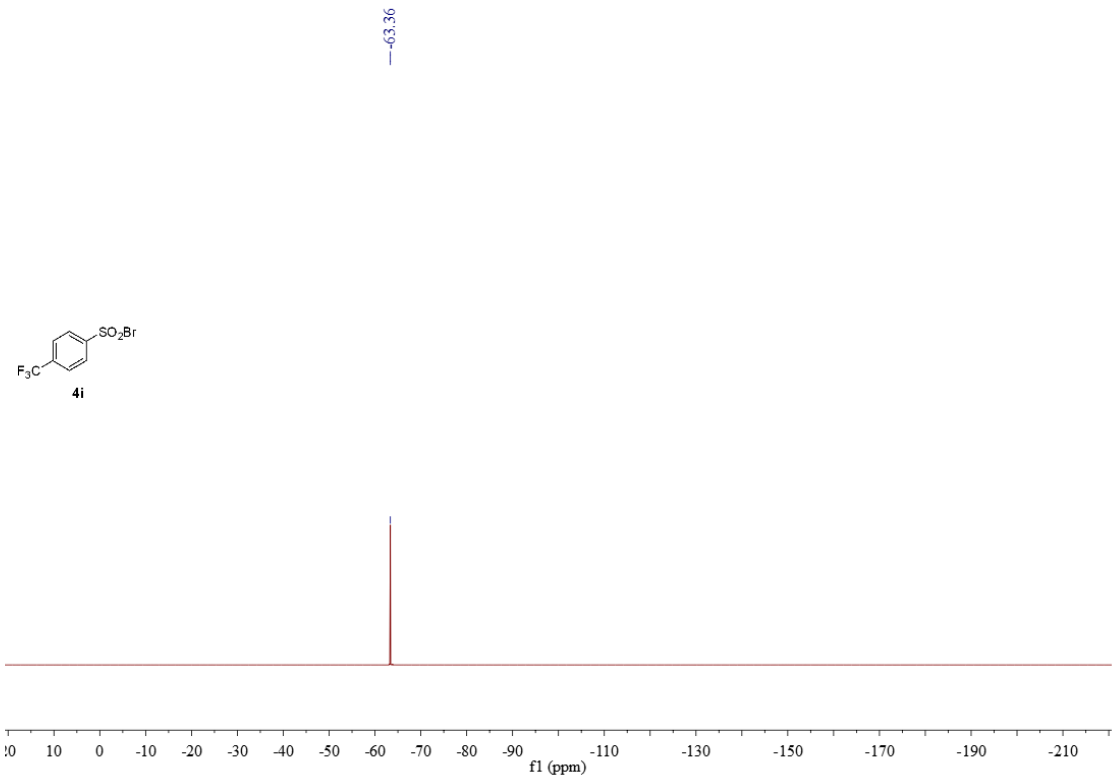
-63.36
SO2Br
F3C
4i
f1 (ppm)

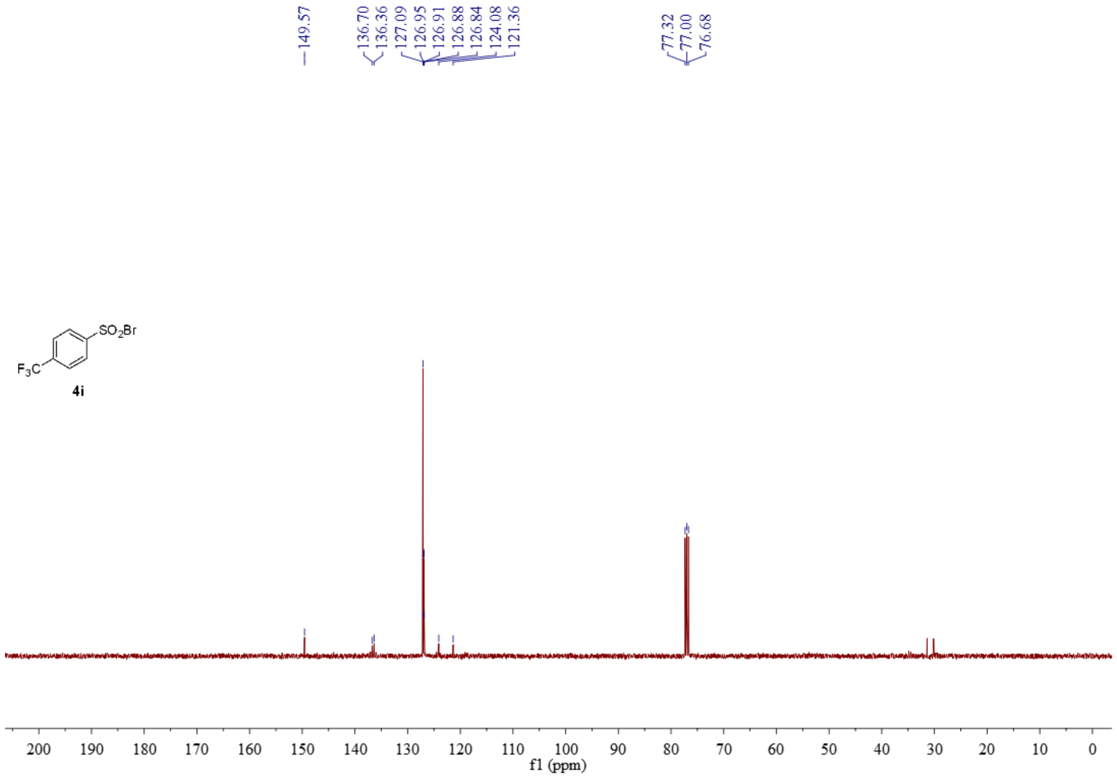
149.57
136.70
136.36
127.09
126.95
126.91
126.88
126.84
124.08
121.36
77.32
77.00
76.68
SO2Br
F3C
4i
f1 (ppm)

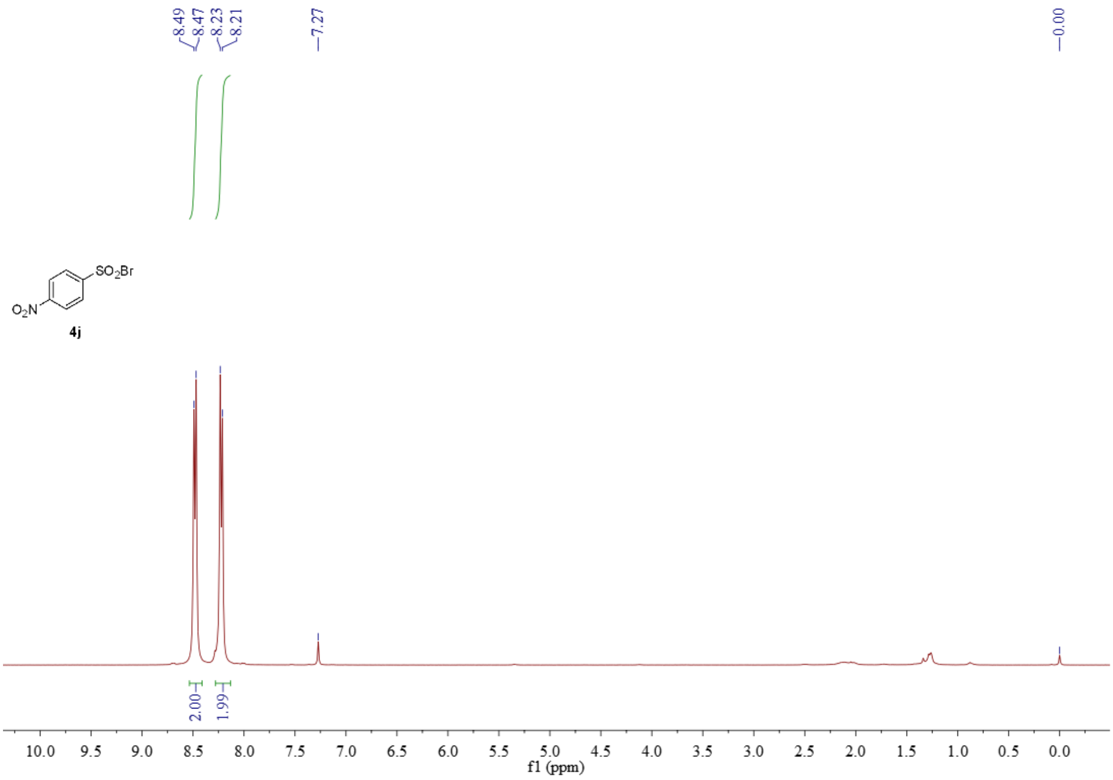
8.49
8.47
8.23
8.21
7.27
0.00
SO2Br
O2N
4j
2.00
1.99
10.0
9.5
9.0
8.5
8.0
7.5
7.0
6.5
6.0
5.5
5.0
4.5
4.0
3.5
3.0
2.5
2.0
1.5
1.0
0.5
0.0
f1 (ppm)

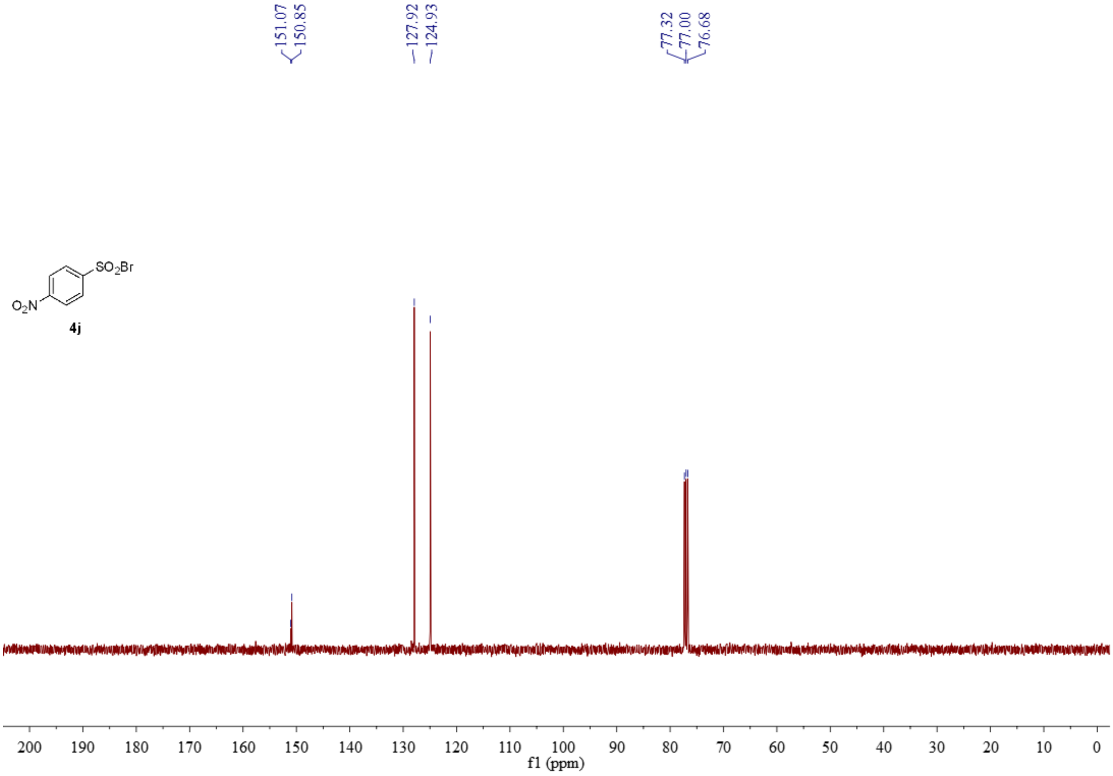
151.07
150.85
127.92
124.93
77.32
77.00
76.68
SO2Br
O2N
4j
200
190
180
170
160
150
140
130
120
110
100
90
80
70
60
50
40
30
20
10
0
f1 (ppm)

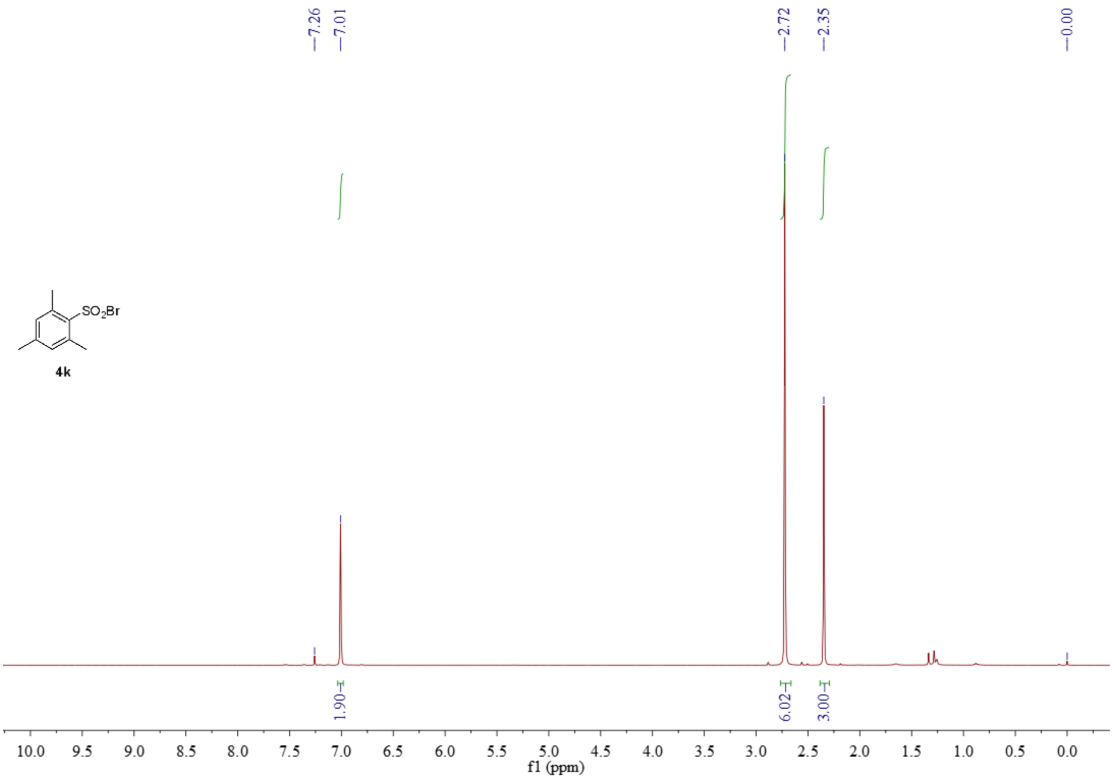
7.26
7.01
2.72
2.35
0.00
SO2Br
4k
1.90
6.02
3.00
f1 (ppm)

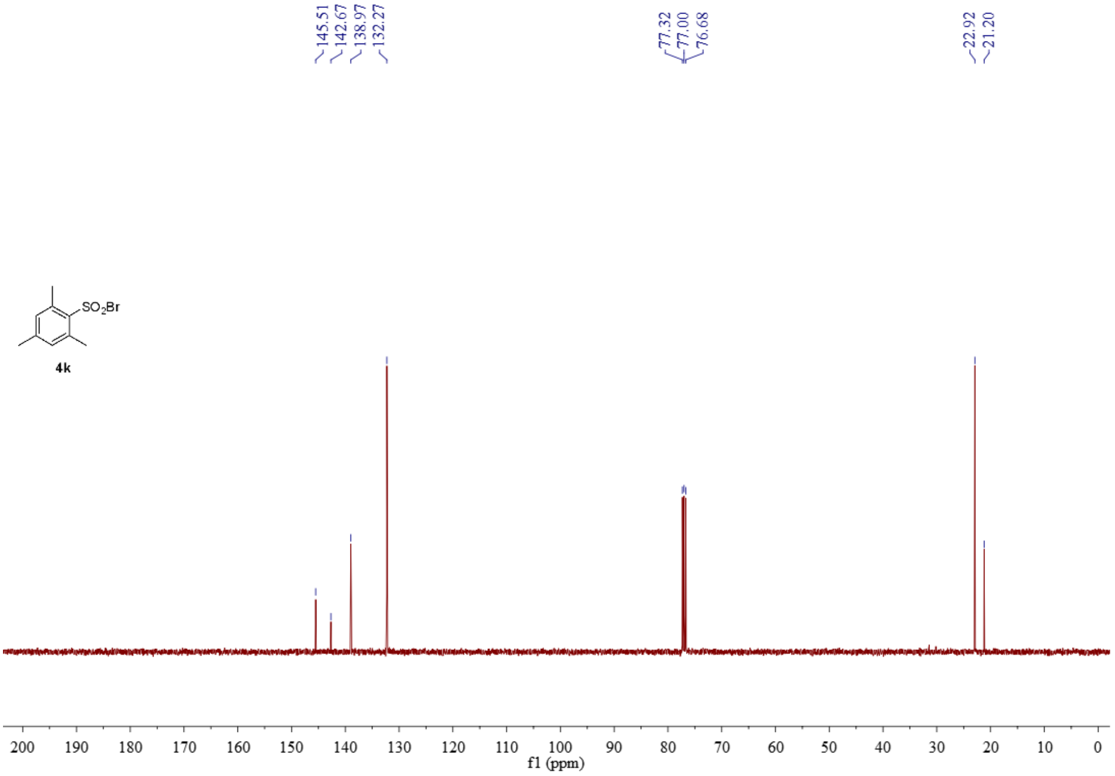
145.51
142.67
138.97
132.27
77.32
77.00
76.68
22.92
21.20
SO2Br
4k
f1 (ppm)

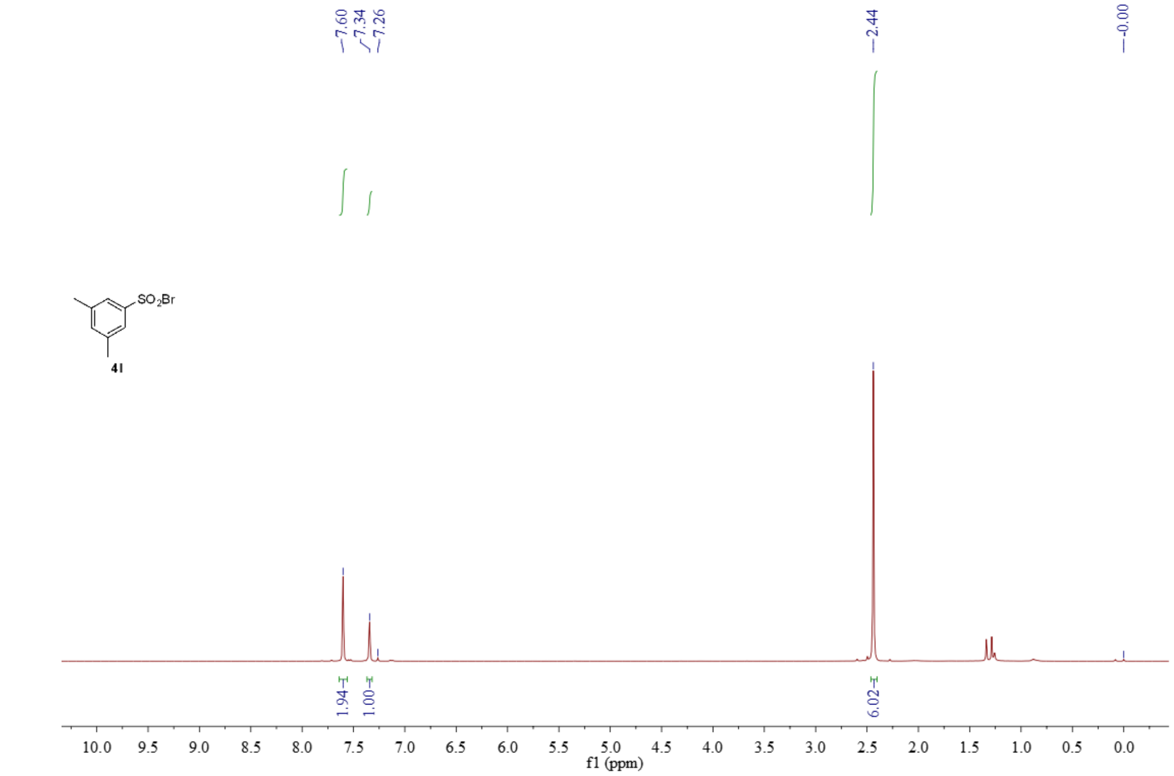
7.60
7.34
7.26
2.44
0.00
SO2Br
4l
1.94
1.00
6.02
10.0 9.5 9.0 8.5 8.0 7.5 7.0 6.5 6.0 5.5 5.0 4.5 4.0 3.5 3.0 2.5 2.0 1.5 1.0 0.5 0.0
f1 (ppm)

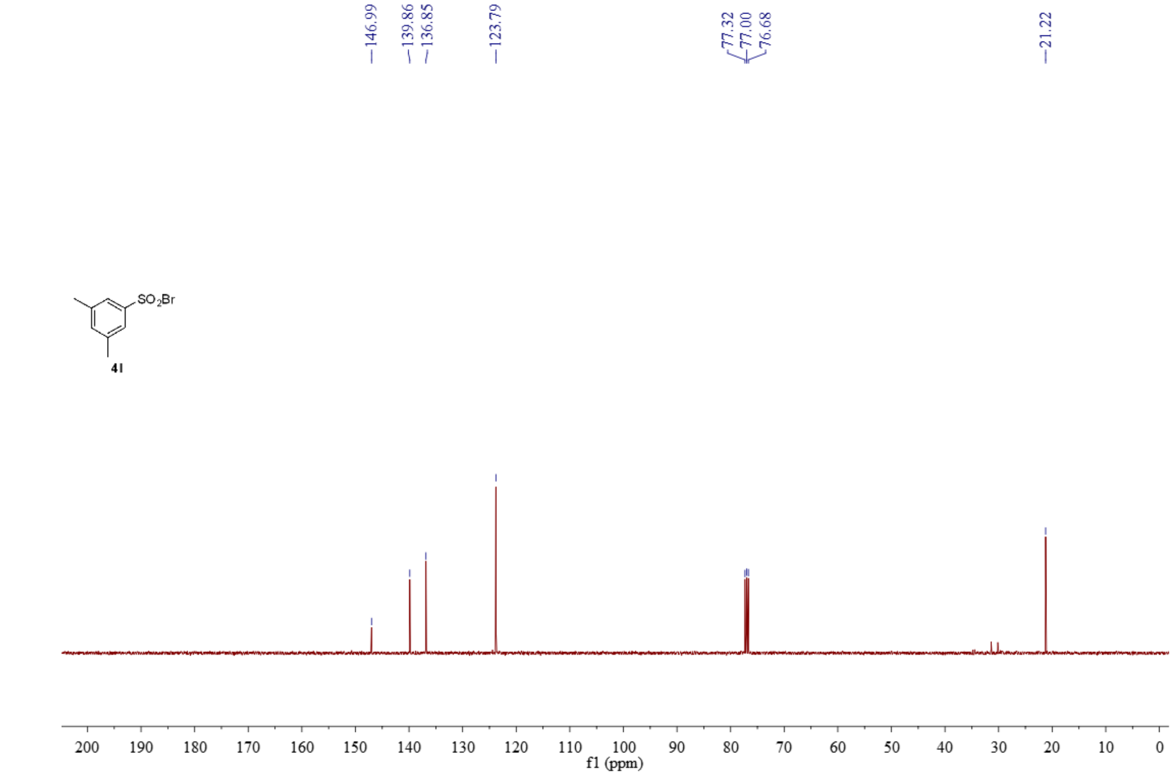
146.99
139.86
136.85
123.79
77.32
77.00
76.68
21.22
SO2Br
4l
200 190 180 170 160 150 140 130 120 110 100 90 80 70 60 50 40 30 20 10 0
f1 (ppm)

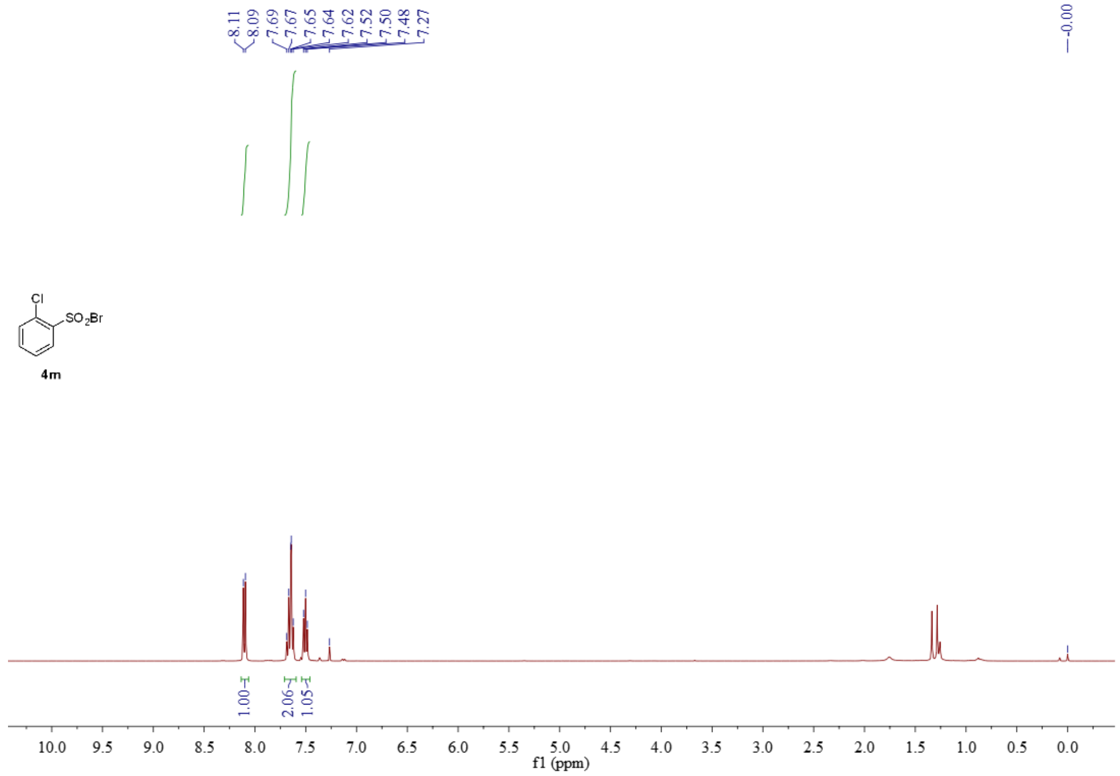
8.11
8.09
7.69
7.67
7.65
7.64
7.62
7.52
7.50
7.48
7.27
0.00
Cl
SO2Br
4m
1.00
2.06
1.05
f1 (ppm)

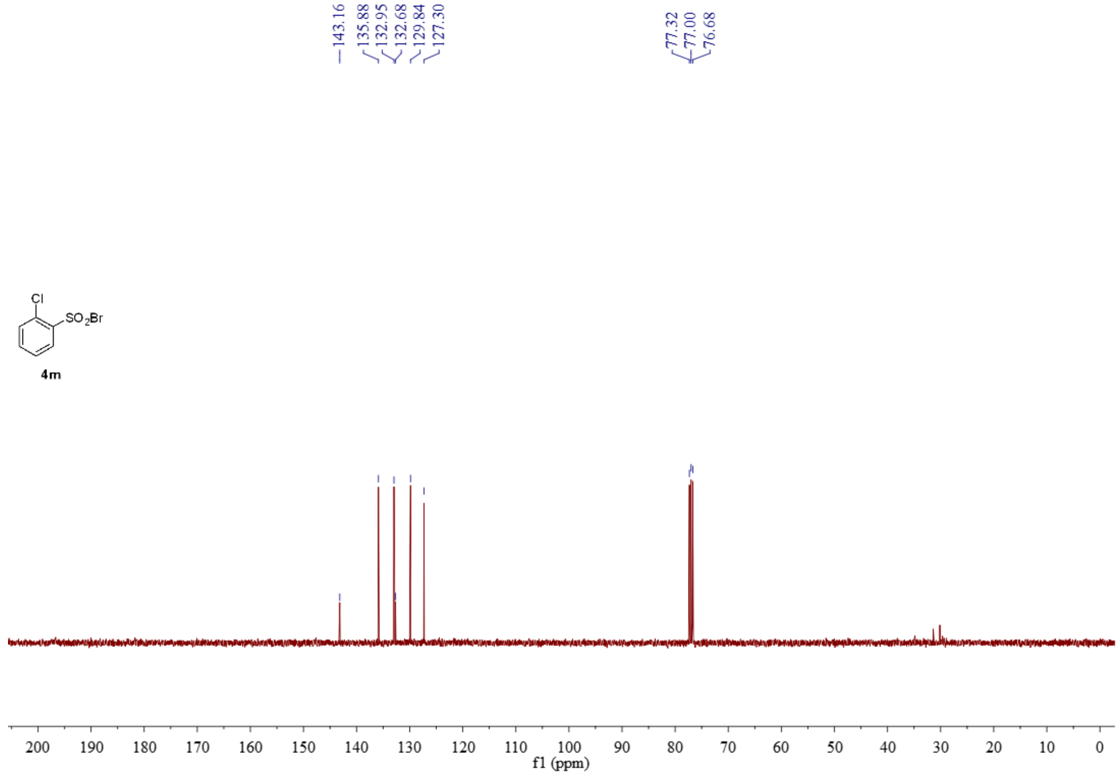
143.16
135.88
132.95
132.68
129.84
127.30
77.32
77.00
76.68
Cl
SO2Br
4m
f1 (ppm)

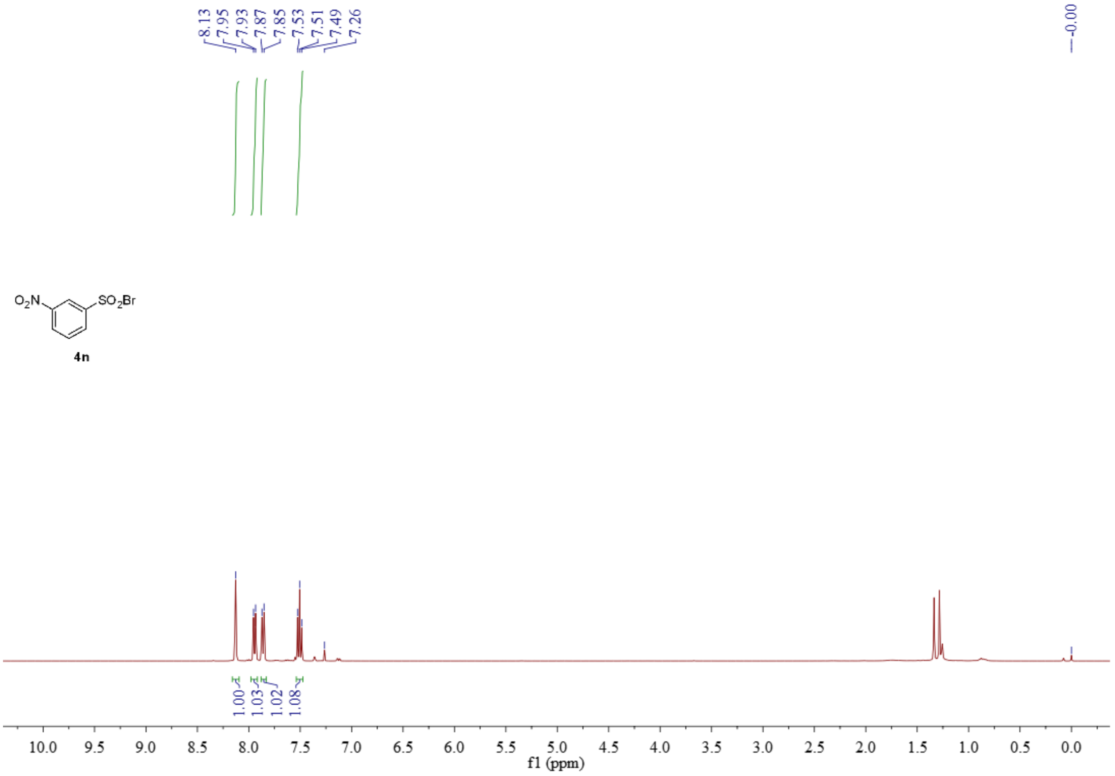
8.13
7.95
7.93
7.87
7.85
7.53
7.51
7.49
7.26
-0.00
O_2N
SO_2Br
4n
1.00
1.03
1.02
1.08
f1 (ppm)

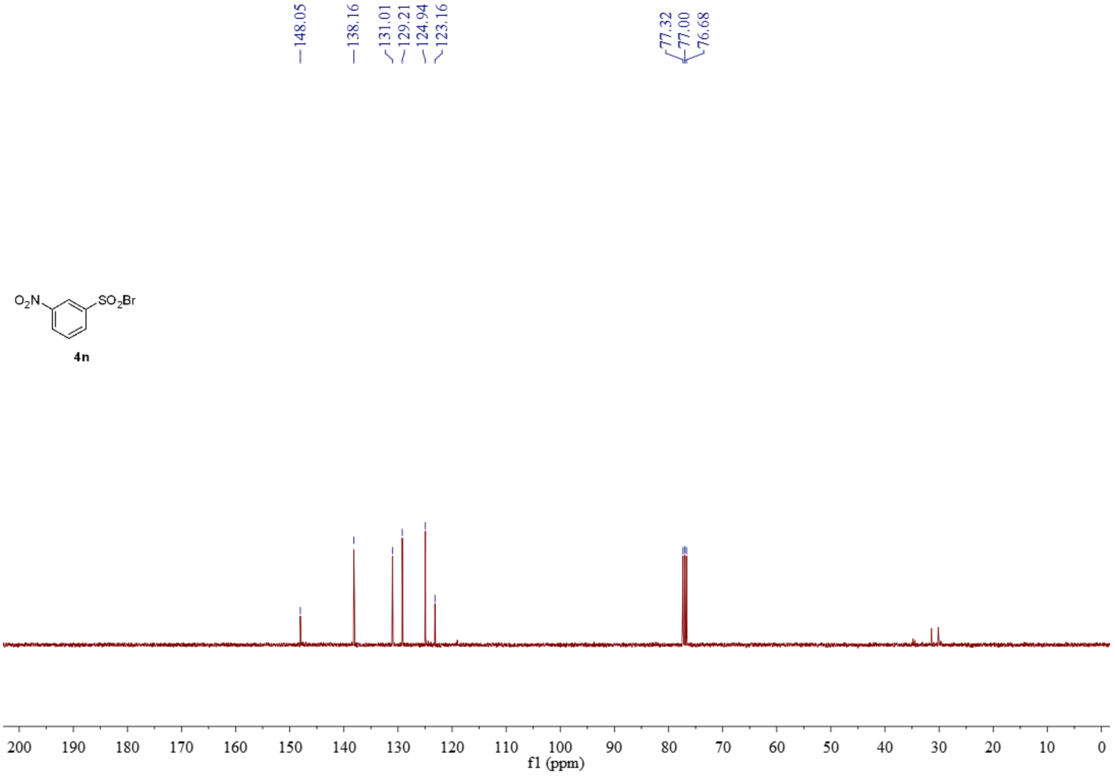
148.05
138.16
131.01
129.21
124.94
123.16
77.32
77.00
76.68
O_2N
SO_2Br
4n
f1 (ppm)

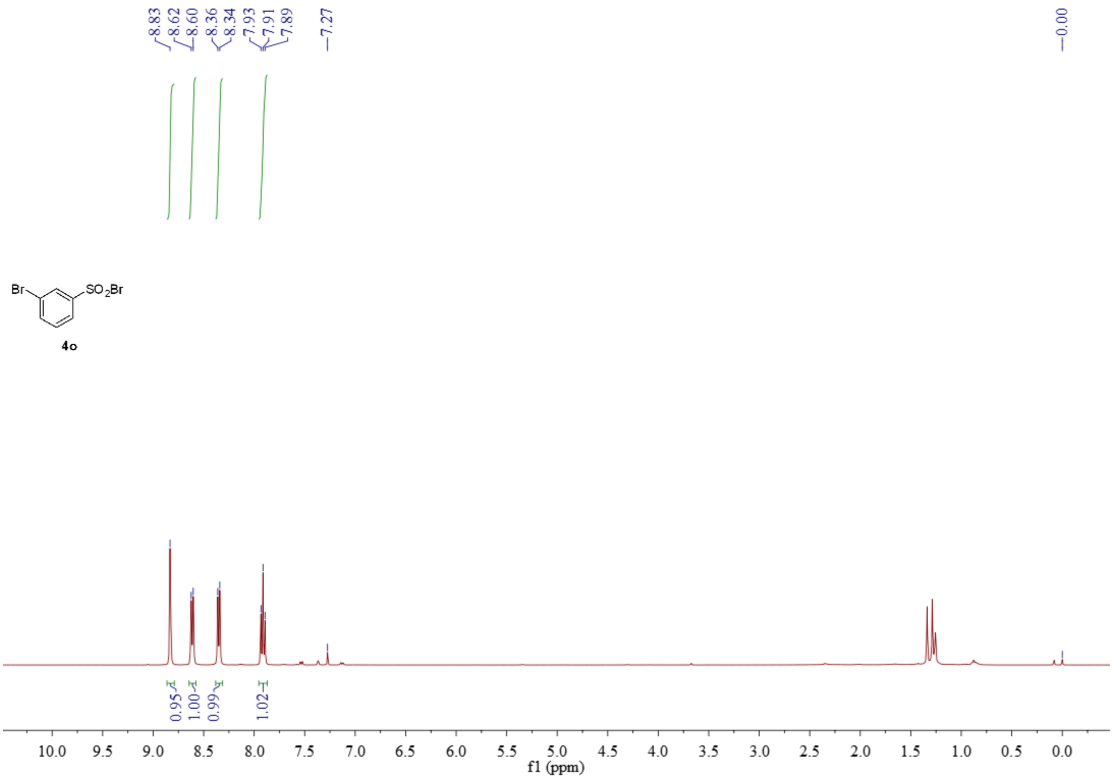
8.83
8.62
8.60
8.36
8.34
7.93
7.91
7.89
7.27
0.00
Br
SO2Br
4o
0.95
1.00
0.99
1.02
10.0 9.5 9.0 8.5 8.0 7.5 7.0 6.5 6.0 5.5 5.0 4.5 4.0 3.5 3.0 2.5 2.0 1.5 1.0 0.5 0.0
f1 (ppm)

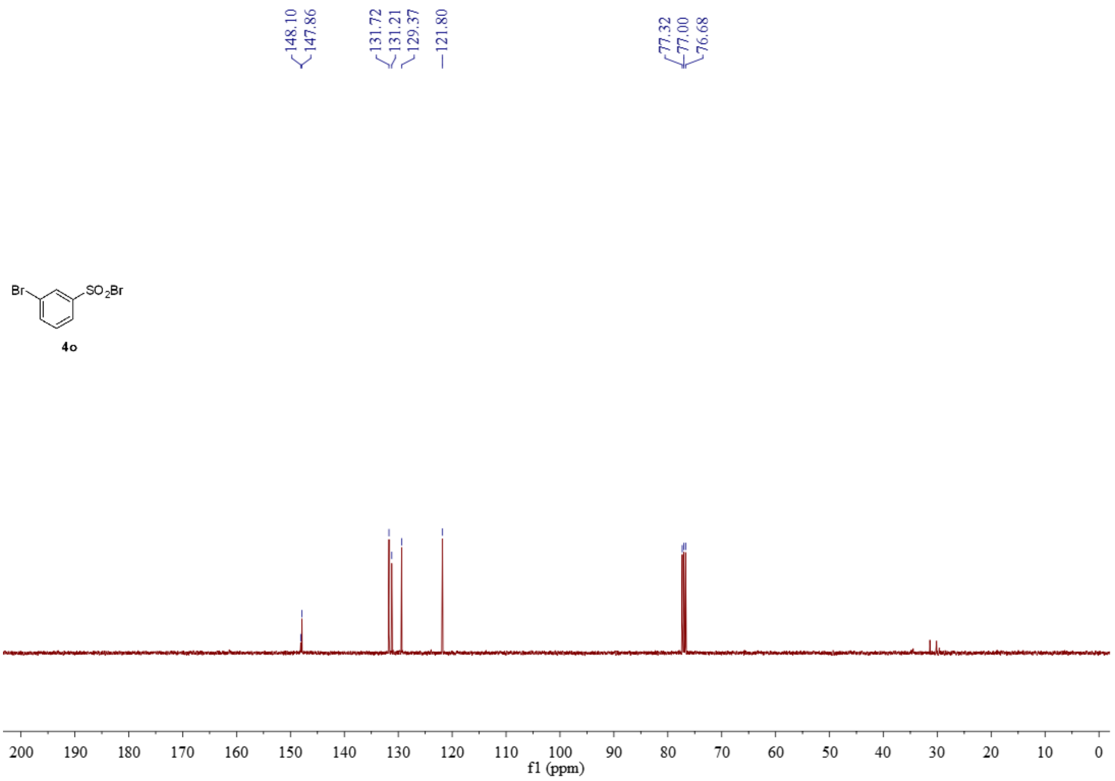
148.10
147.86
131.72
131.21
129.37
121.80
77.32
77.00
76.68
Br
SO2Br
4o
200 190 180 170 160 150 140 130 120 110 100 90 80 70 60 50 40 30 20 10 0
f1 (ppm)

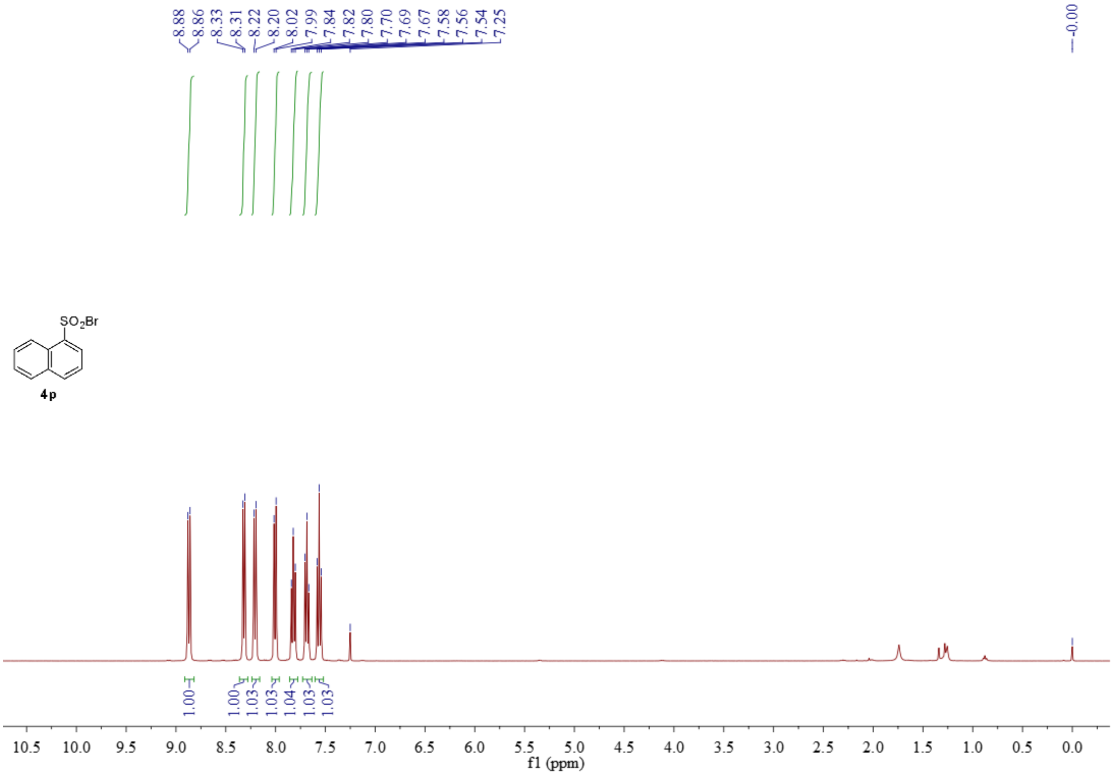
SO2Br
4p
f1 (ppm)

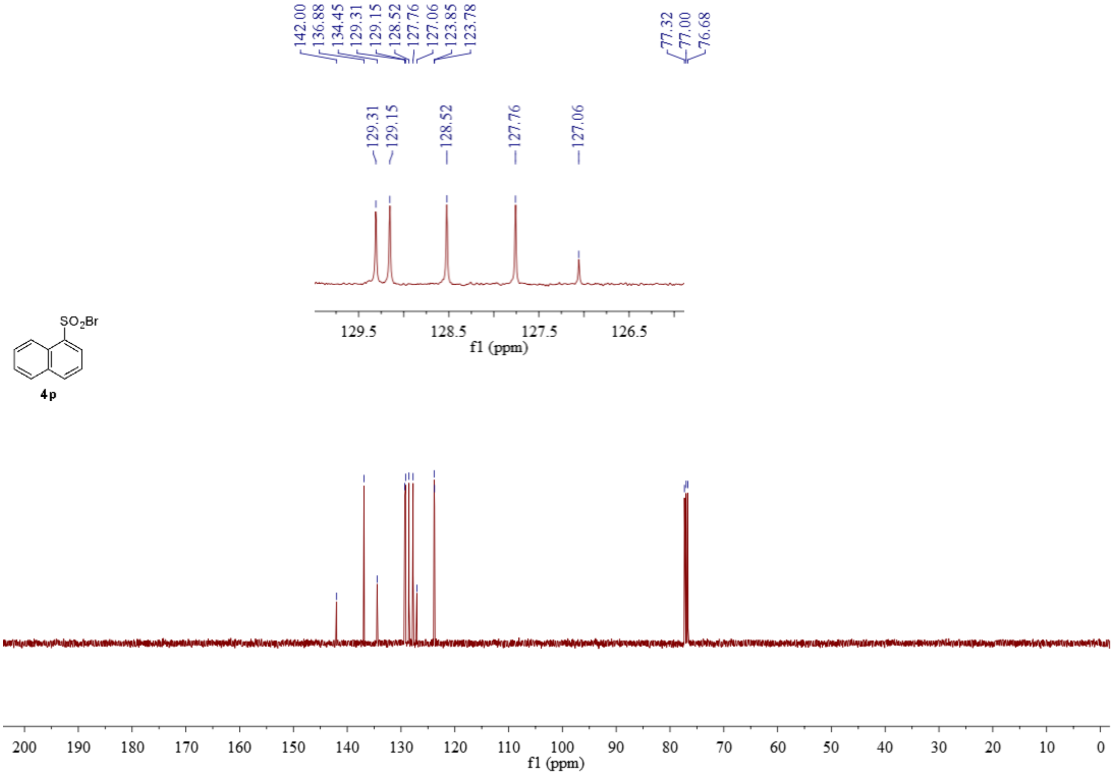
SO2Br
4p
f1 (ppm)

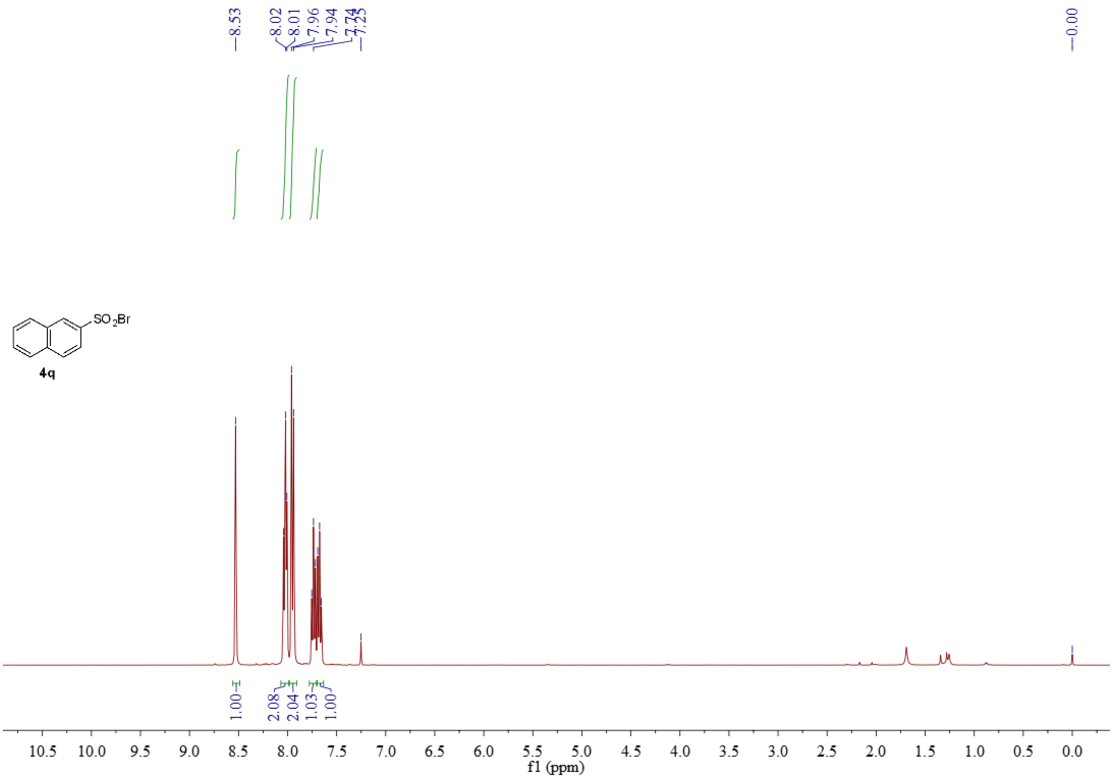
8.53
8.02
8.01
7.96
7.94
7.74
7.25
0.00
SO2Br
4q
1.00
2.08
2.04
1.03
1.00
10.5 10.0 9.5 9.0 8.5 8.0 7.5 7.0 6.5 6.0 5.5 5.0 4.5 4.0 3.5 3.0 2.5 2.0 1.5 1.0 0.5 0.0
f1 (ppm)

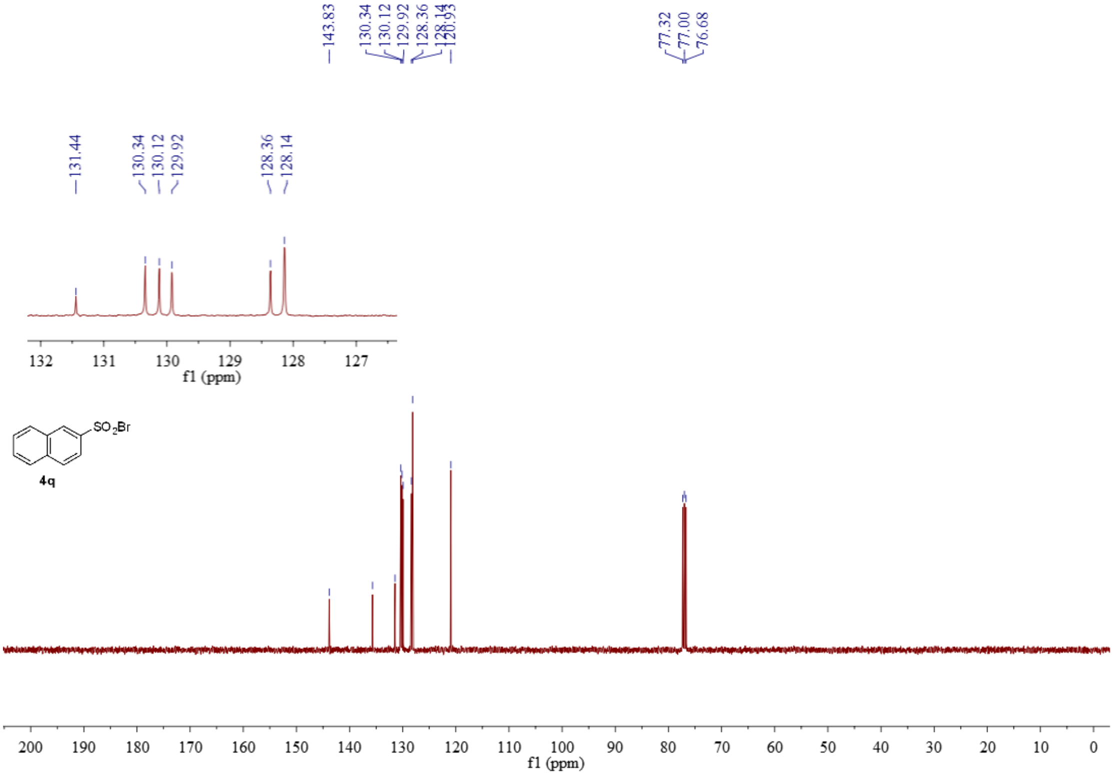
143.83
130.34
130.12
129.92
128.36
128.14
120.93
77.32
77.00
76.68
131.44
130.34
130.12
129.92
128.36
128.14
132 131 130 129 128 127
f1 (ppm)
SO2Br
4q
200 190 180 170 160 150 140 130 120 110 100 90 80 70 60 50 40 30 20 10 0
f1 (ppm)

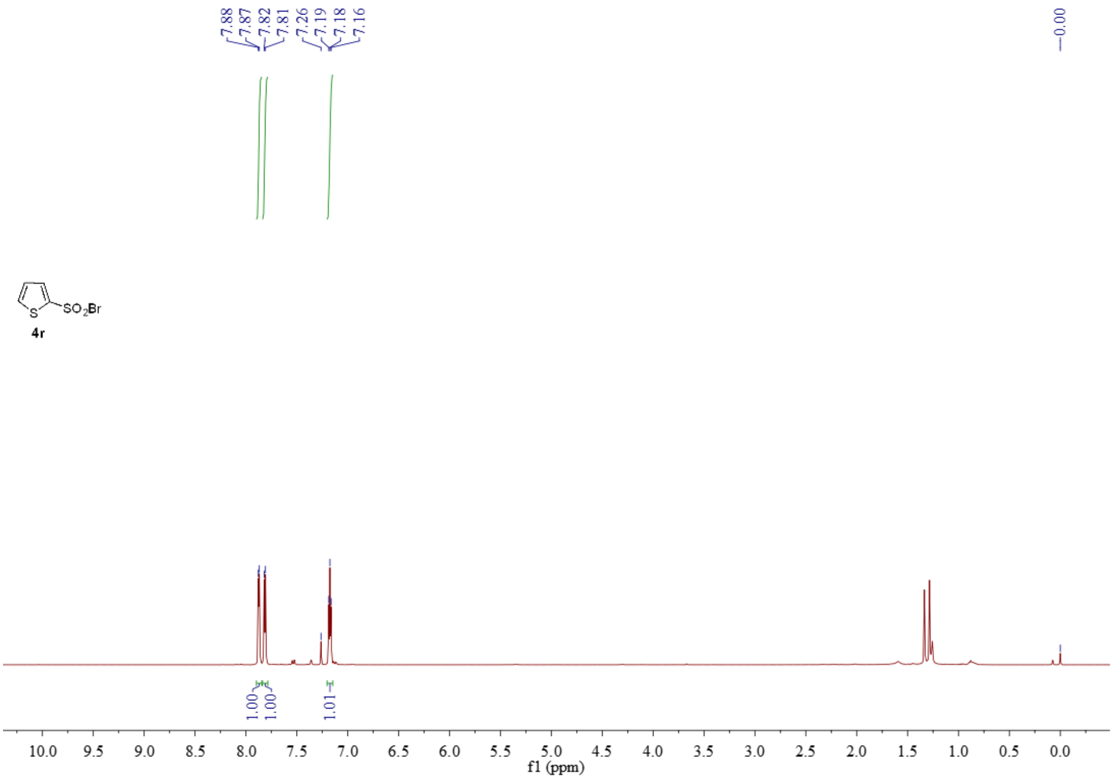
7.88
7.87
7.82
7.81
7.26
7.19
7.18
7.16
0.00
SO2Br
S
4r
1.00
1.00
1.01
f1 (ppm)

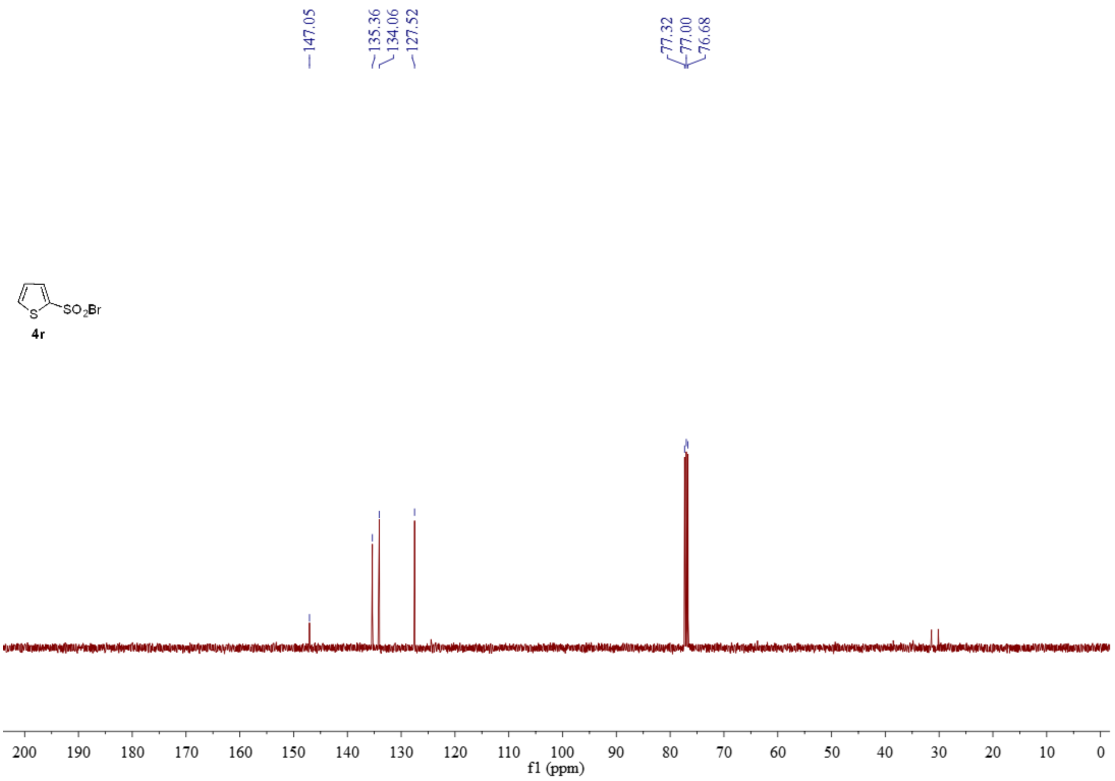
147.05
135.36
134.06
127.52
77.32
77.00
76.68
SO2Br
S
4r
f1 (ppm)

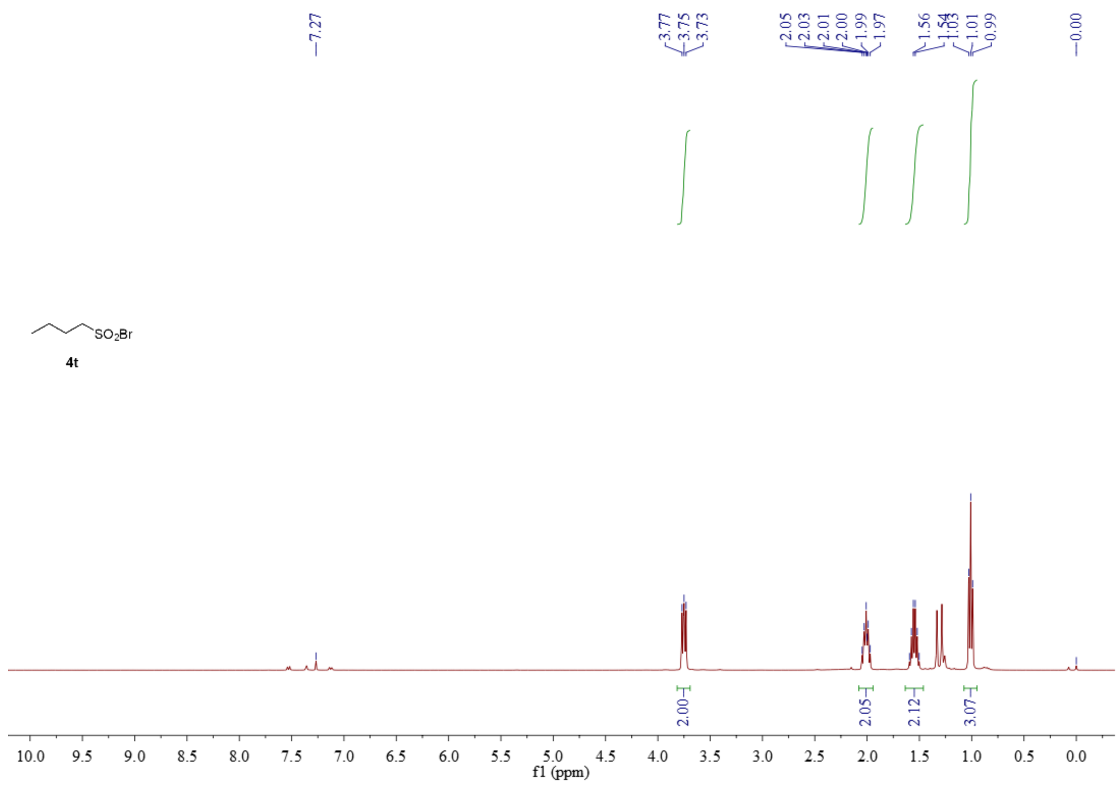
7.27
3.77
3.75
3.73
2.05
2.03
2.01
2.00
1.99
1.97
1.56
1.54
1.01
0.99
0.00
SO2Br
4t
2.00
2.05
2.12
3.07
10.0 9.5 9.0 8.5 8.0 7.5 7.0 6.5 6.0 5.5 5.0 4.5 4.0 3.5 3.0 2.5 2.0 1.5 1.0 0.5 0.0
f1 (ppm)

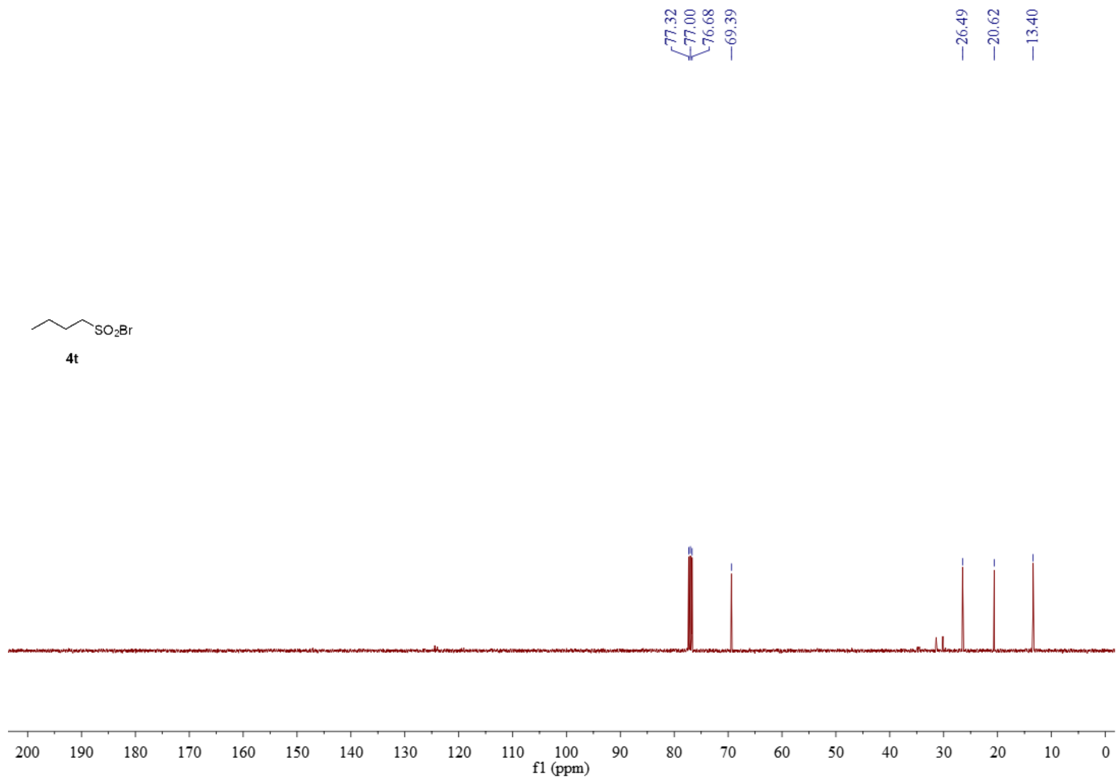
77.32
77.00
76.68
69.39
26.49
20.62
13.40
SO2Br
4t
200 190 180 170 160 150 140 130 120 110 100 90 80 70 60 50 40 30 20 10 0
f1 (ppm)

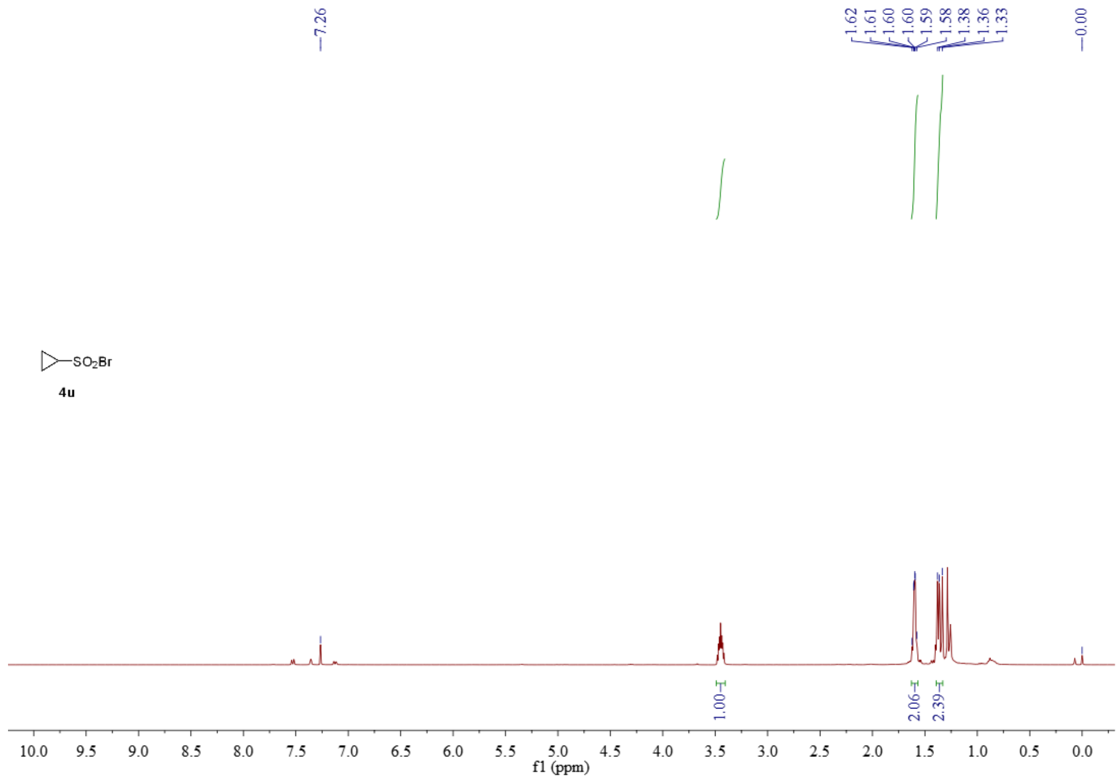

7.26
1.62
1.61
1.60
1.60
1.59
1.58
1.38
1.36
1.33
0.00
SO2Br
4u
1.00
2.06
2.39
10.0 9.5 9.0 8.5 8.0 7.5 7.0 6.5 6.0 5.5 5.0 4.5 4.0 3.5 3.0 2.5 2.0 1.5 1.0 0.5 0.0
f1 (ppm)

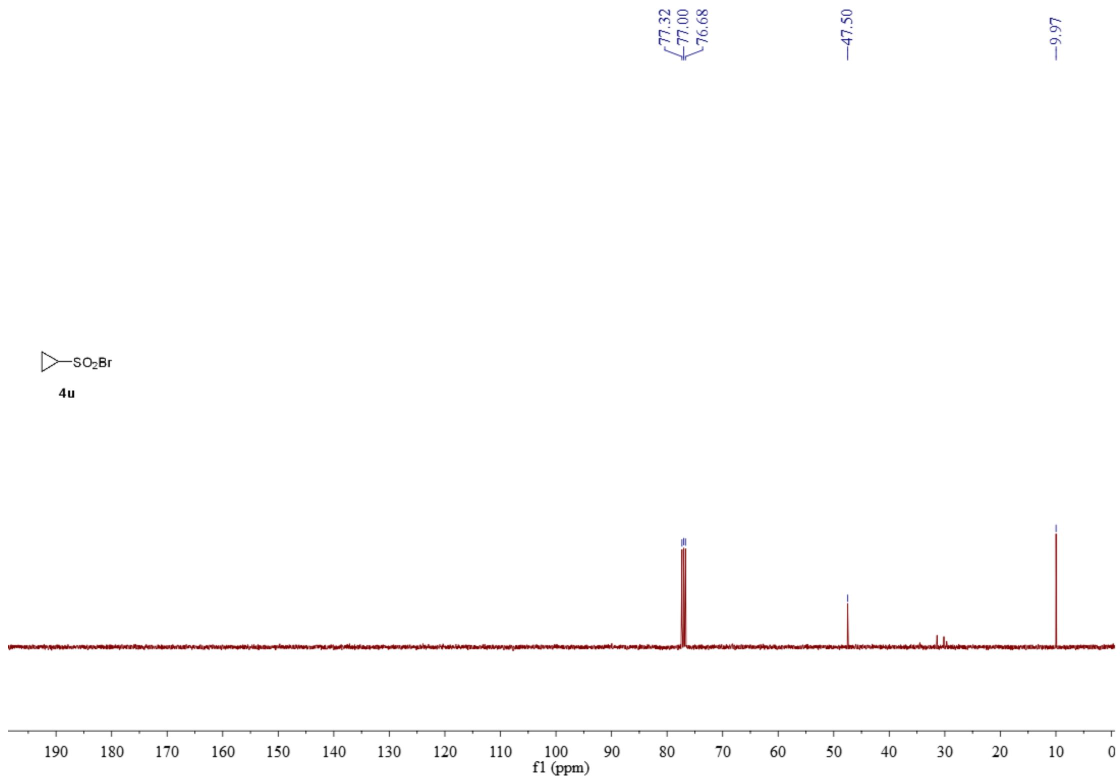

77.32
77.00
76.68
47.50
9.97
SO2Br
4u
190 180 170 160 150 140 130 120 110 100 90 80 70 60 50 40 30 20 10 0
f1 (ppm)

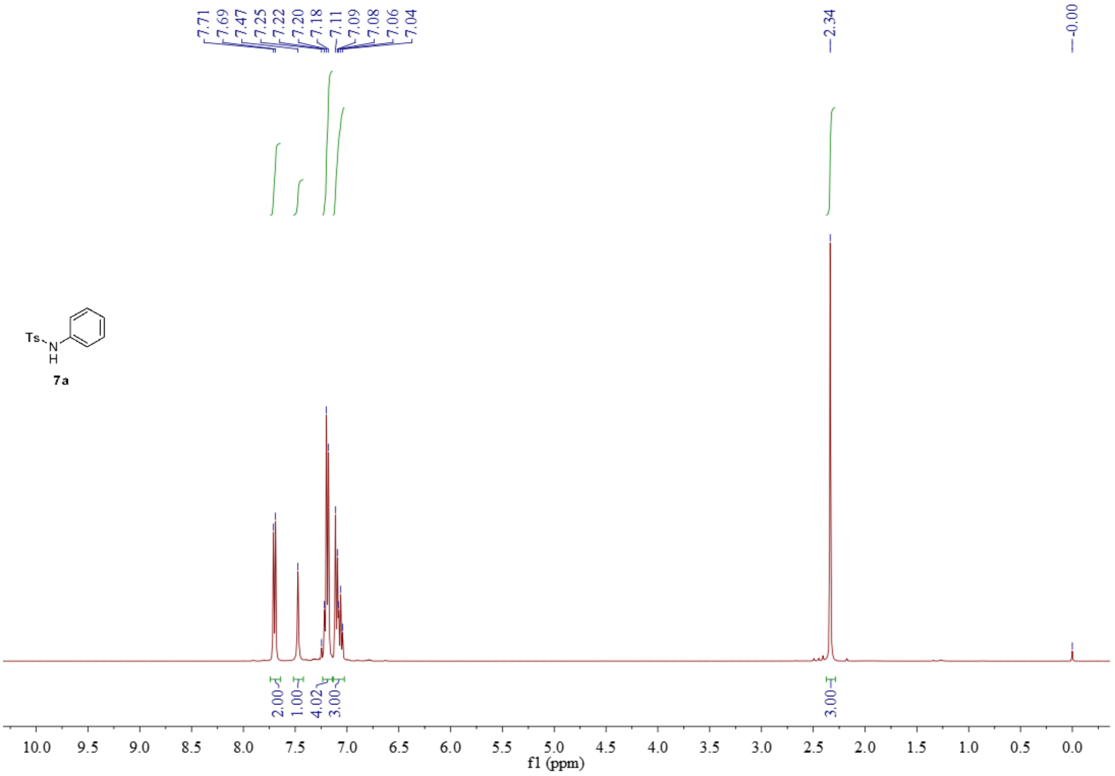
7.71 7.69 7.47 7.25 7.22 7.20 7.18 7.11 7.09 7.08 7.06 7.04
2.34
-0.00
Ts N H
7a
2.00 1.00 4.02 3.00
3.00
10.0 9.5 9.0 8.5 8.0 7.5 7.0 6.5 6.0 5.5 5.0 4.5 4.0 3.5 3.0 2.5 2.0 1.5 1.0 0.5 0.0
f1 (ppm)

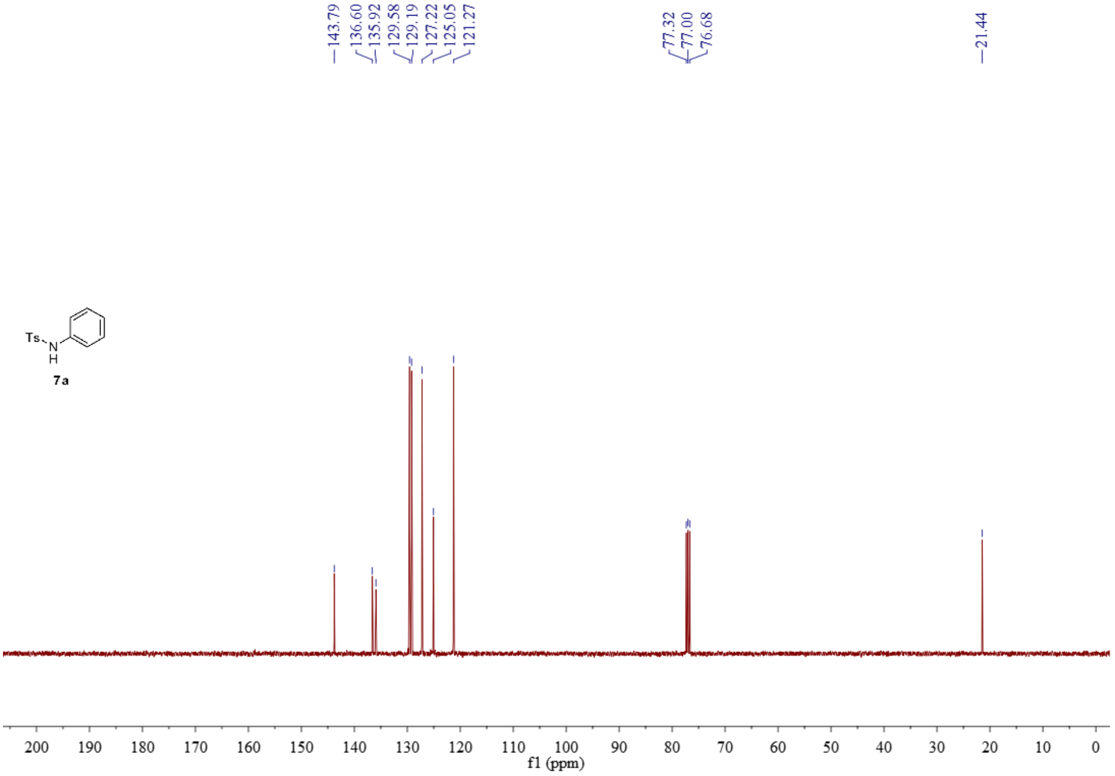
143.79 136.60 135.92 129.58 129.19 127.22 125.05 121.27
77.32 77.00 76.68
21.44
Ts N H
7a
200 190 180 170 160 150 140 130 120 110 100 90 80 70 60 50 40 30 20 10 0
f1 (ppm)

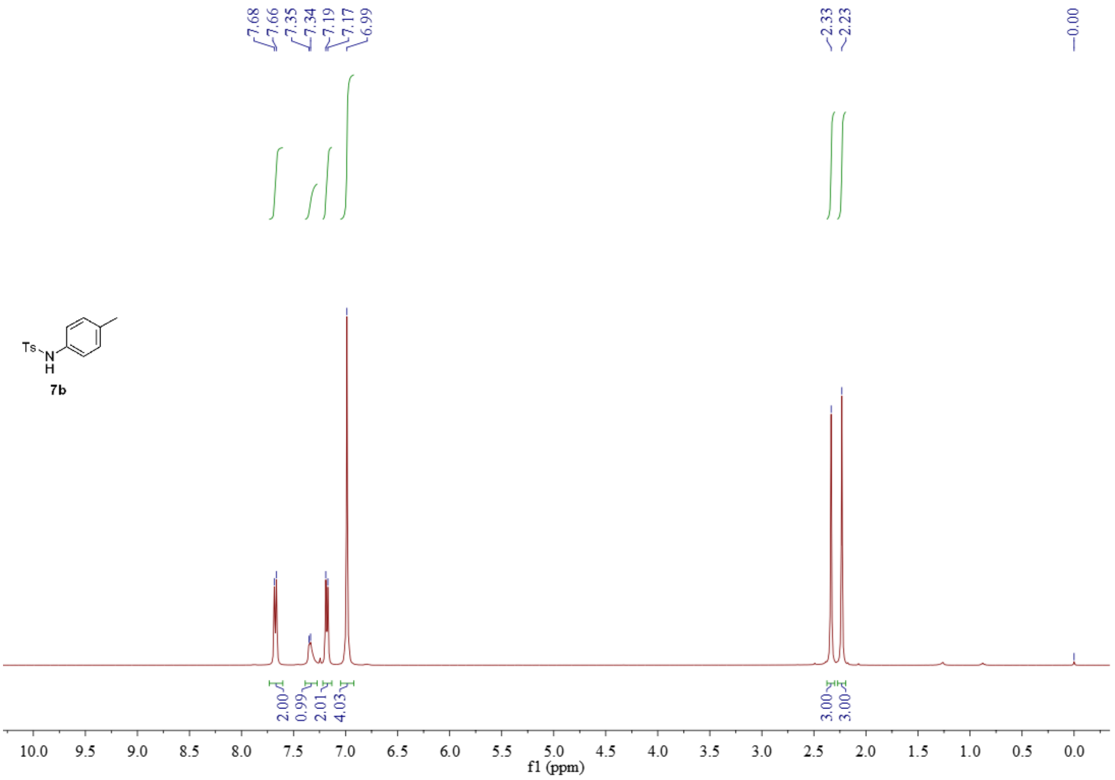
7.68
7.66
7.35
7.34
7.19
7.17
6.99
2.33
2.23
0.00
Ts
N
H
7b
2.00
0.99
2.01
4.03
3.00
3.00
10.0 9.5 9.0 8.5 8.0 7.5 7.0 6.5 6.0 5.5 5.0 4.5 4.0 3.5 3.0 2.5 2.0 1.5 1.0 0.5 0.0
f1 (ppm)

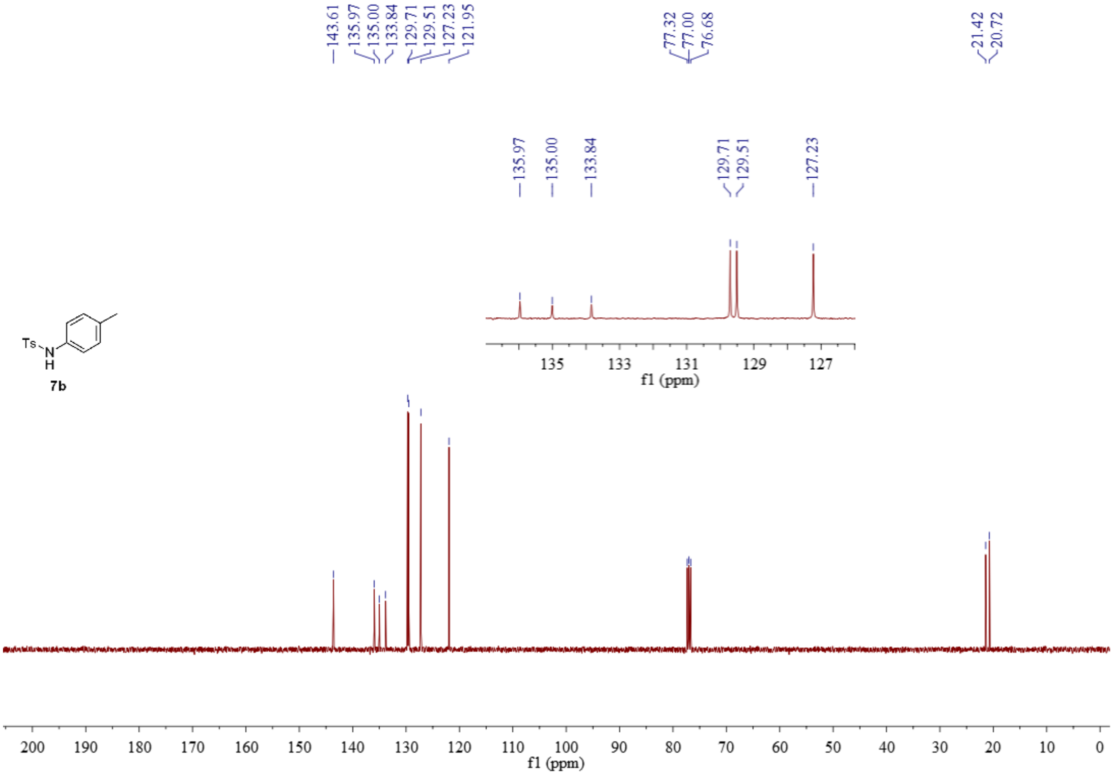
143.61
135.97
135.00
133.84
129.71
129.51
127.23
121.95
77.32
77.00
76.68
21.42
20.72
135.97
135.00
133.84
129.71
129.51
127.23
135 133 131 129 127
f1 (ppm)
Ts
N
H
7b
200 190 180 170 160 150 140 130 120 110 100 90 80 70 60 50 40 30 20 10 0
f1 (ppm)

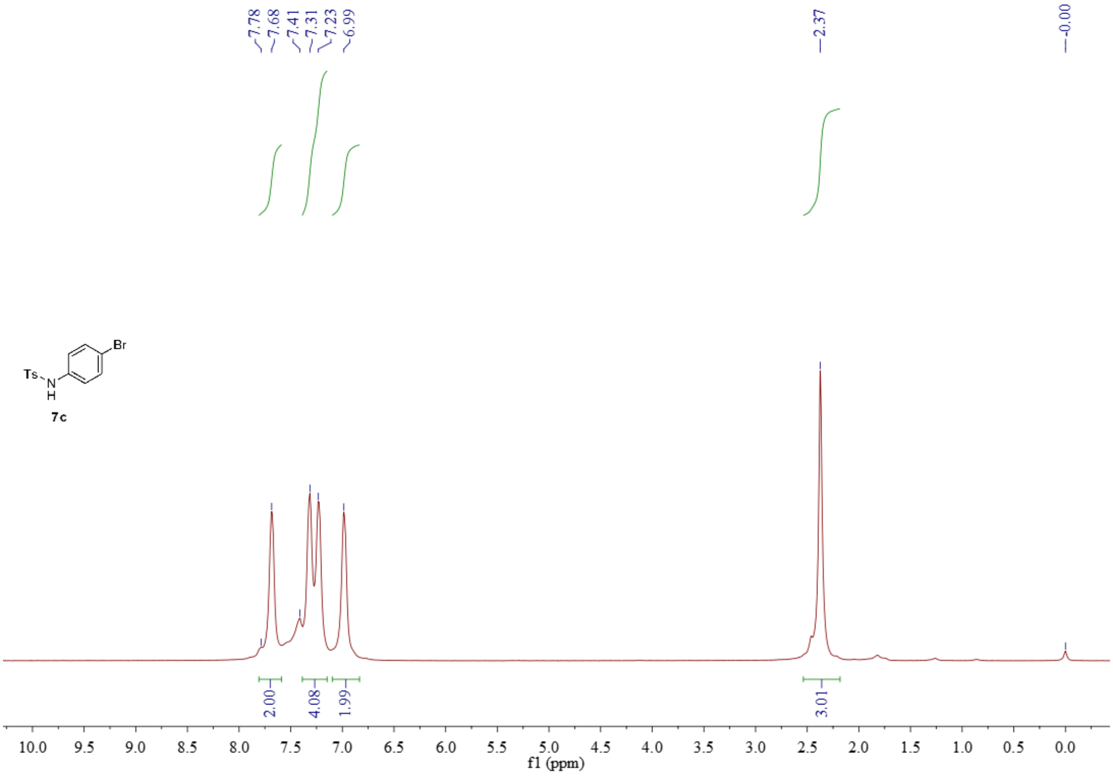
7.78
7.68
7.41
7.31
7.23
6.99
2.37
0.00
Ts
N
H
Br
7c
2.00
4.08
1.99
3.01
10.0
9.5
9.0
8.5
8.0
7.5
7.0
6.5
6.0
5.5
5.0
4.5
4.0
3.5
3.0
2.5
2.0
1.5
1.0
0.5
0.0
f1 (ppm)

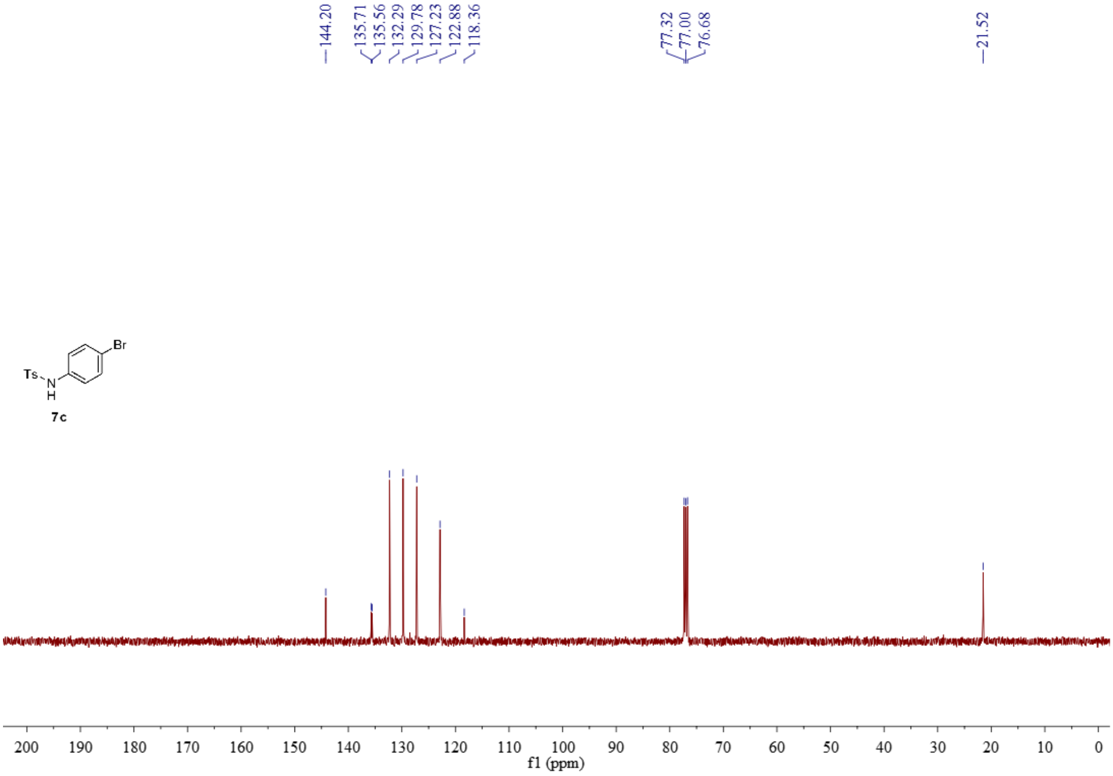
144.20
135.71
135.56
132.29
129.78
127.23
122.88
118.36
77.32
77.00
76.68
21.52
Ts
N
H
Br
7c
200
190
180
170
160
150
140
130
120
110
100
90
80
70
60
50
40
30
20
10
0
f1 (ppm)

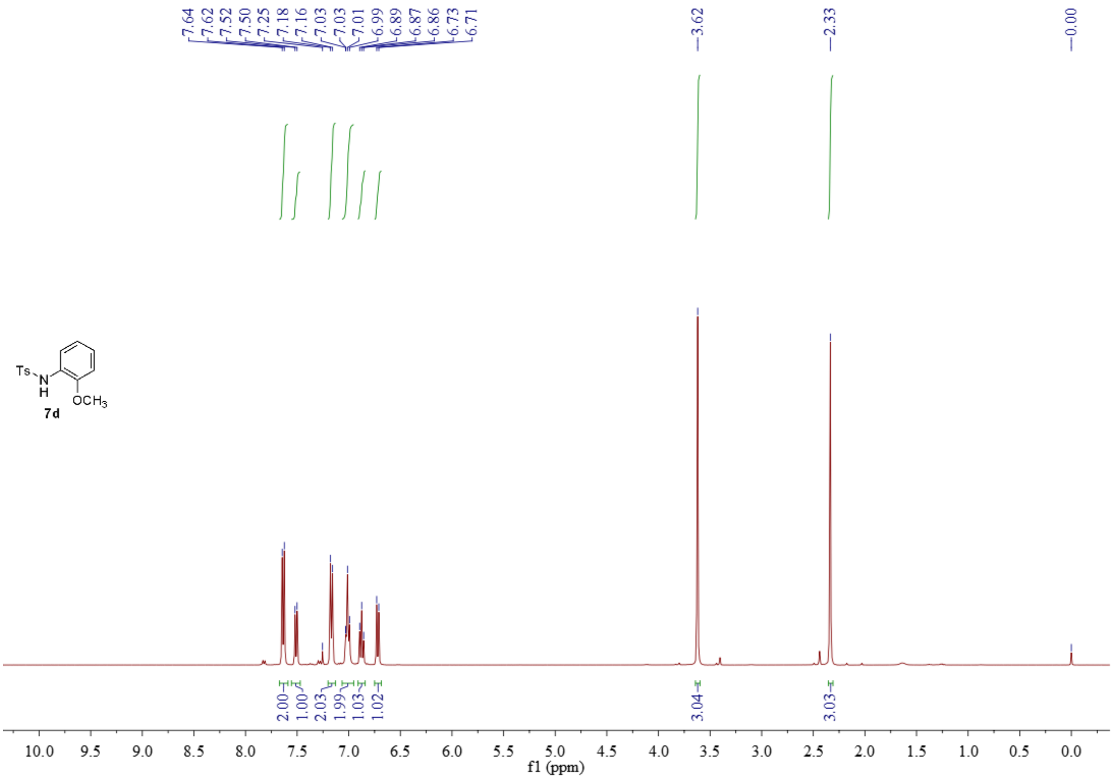
7.64
7.62
7.52
7.50
7.25
7.18
7.16
7.03
7.03
7.01
6.99
6.89
6.87
6.86
6.73
6.71
3.62
2.33
0.00
Ts
N
H
OCH3
7d
2.00
1.00
2.03
1.99
1.03
1.02
3.04
3.03
f1 (ppm)

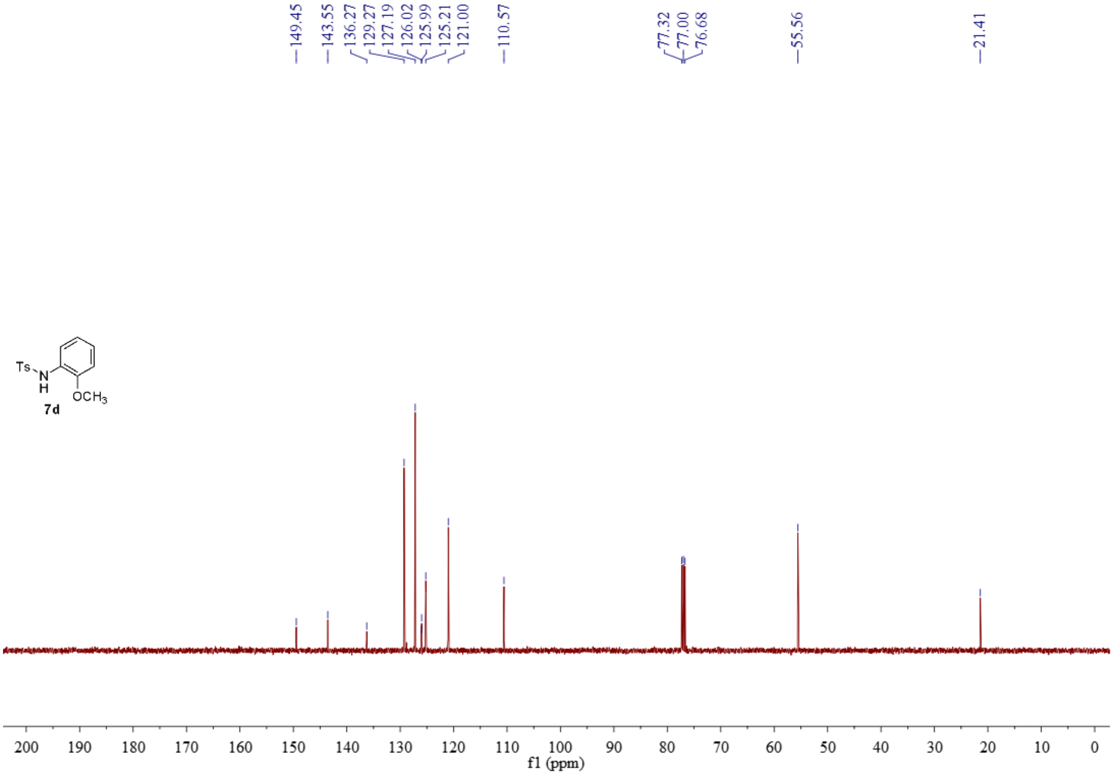
149.45
143.55
136.27
129.27
127.19
126.02
125.99
125.21
121.00
110.57
77.32
77.00
76.68
55.56
21.41
Ts
N
H
OCH3
7d
f1 (ppm)

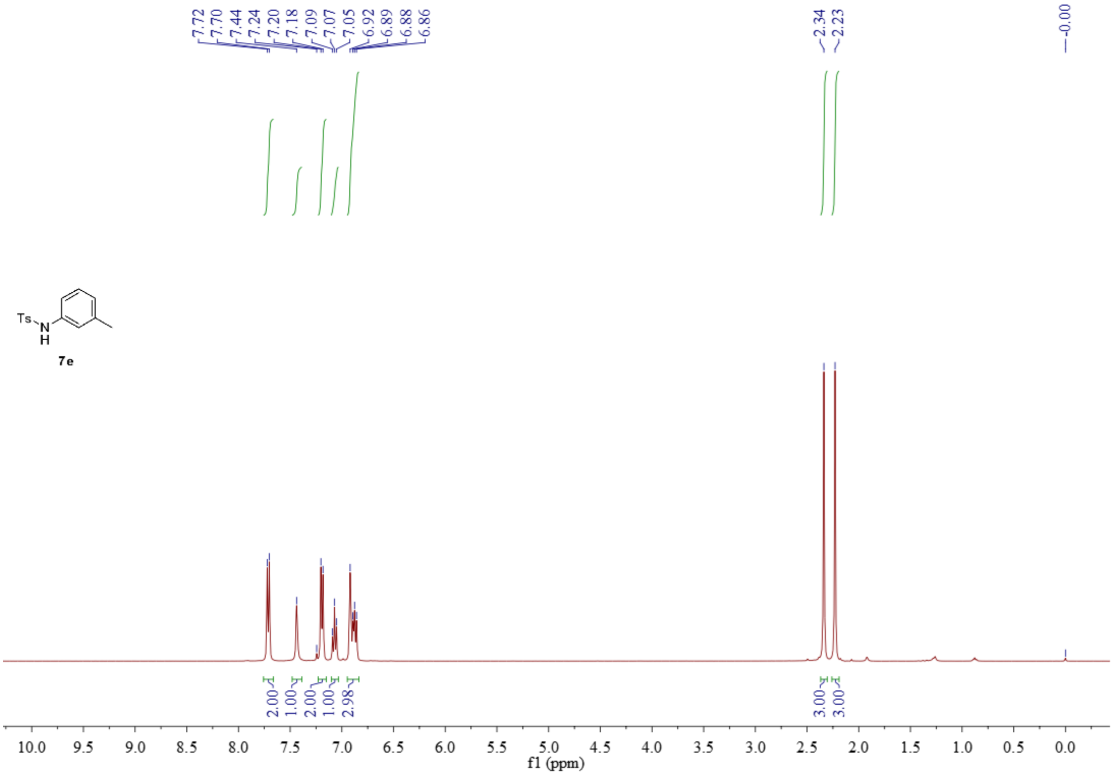
7.72
7.70
7.44
7.24
7.20
7.18
7.09
7.07
7.05
6.92
6.89
6.88
6.86
2.34
2.23
0.00
Ts
N
H
7e
2.00
1.00
2.00
1.00
2.98
3.00
3.00
10.0 9.5 9.0 8.5 8.0 7.5 7.0 6.5 6.0 5.5 5.0 4.5 4.0 3.5 3.0 2.5 2.0 1.5 1.0 0.5 0.0
f1 (ppm)

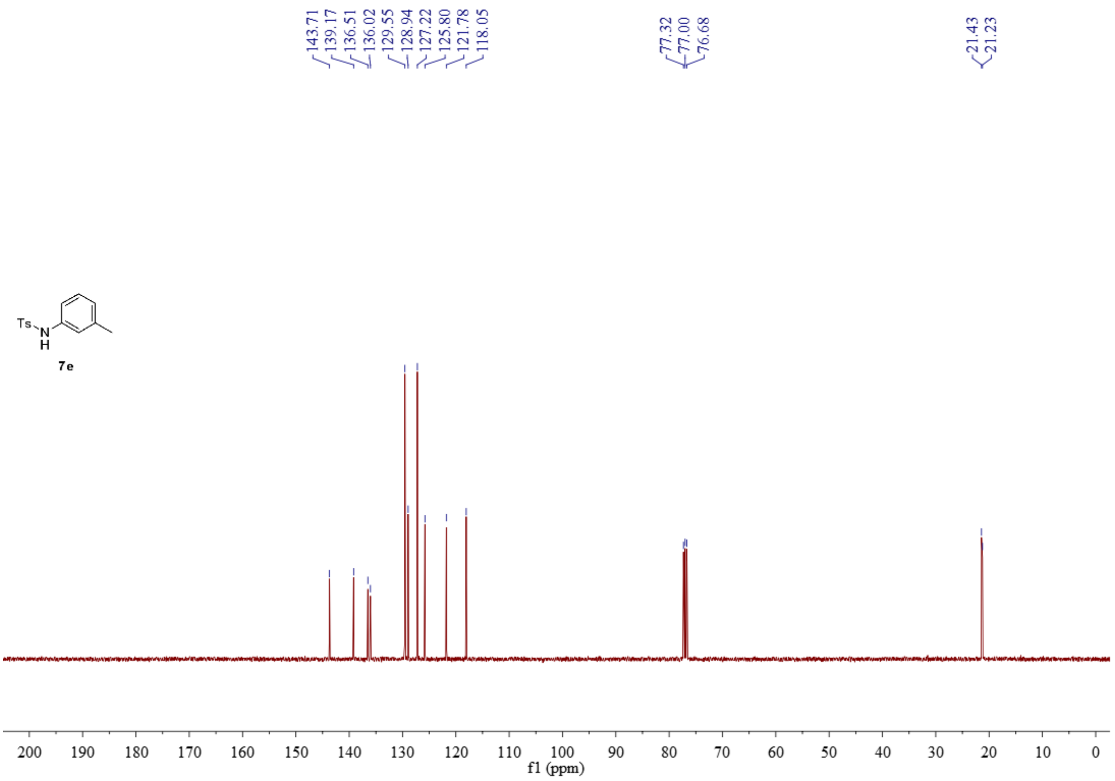
143.71
139.17
136.51
136.02
129.55
128.94
127.22
125.80
121.78
118.05
77.32
77.00
76.68
21.43
21.23
Ts
N
H
7e
200 190 180 170 160 150 140 130 120 110 100 90 80 70 60 50 40 30 20 10 0
f1 (ppm)

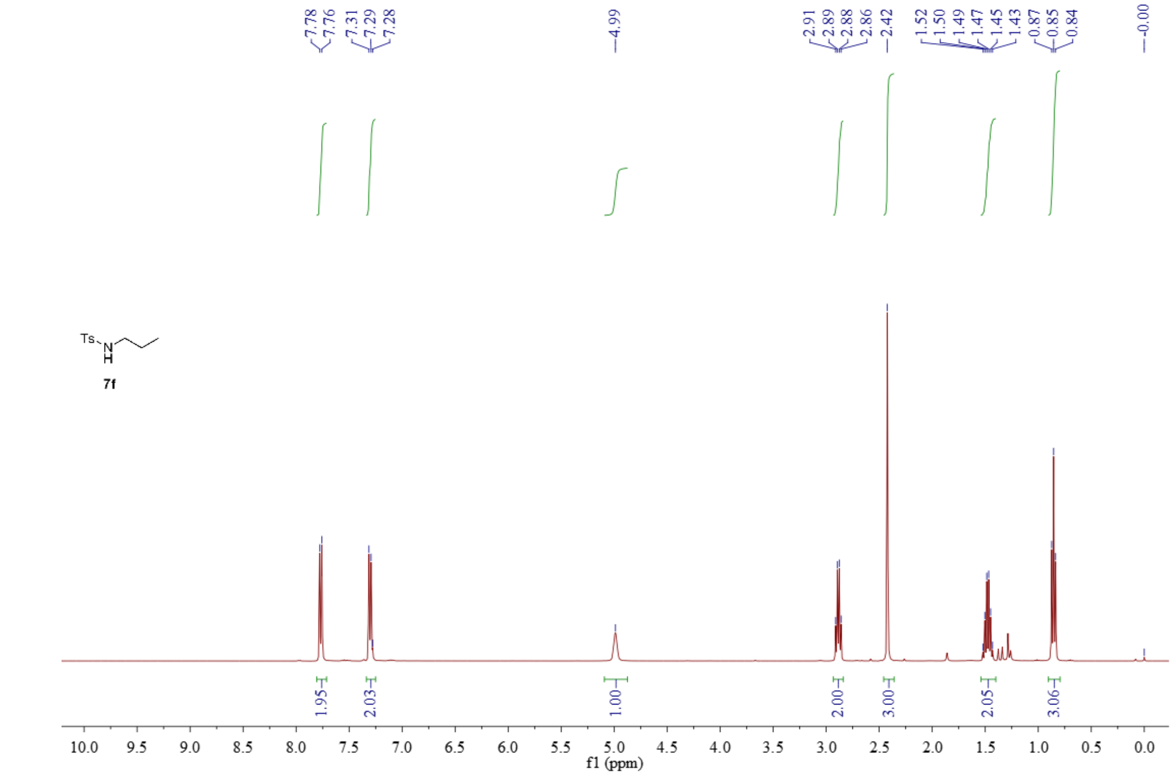
7.78
7.76
7.31
7.29
7.28
4.99
2.91
2.89
2.88
2.86
2.42
1.52
1.50
1.49
1.47
1.45
1.43
0.87
0.85
0.84
-0.00
Ts
N
H
7f
1.95
2.03
1.00
2.00
3.00
2.05
3.06
10.0 9.5 9.0 8.5 8.0 7.5 7.0 6.5 6.0 5.5 5.0 4.5 4.0 3.5 3.0 2.5 2.0 1.5 1.0 0.5 0.0
f1 (ppm)

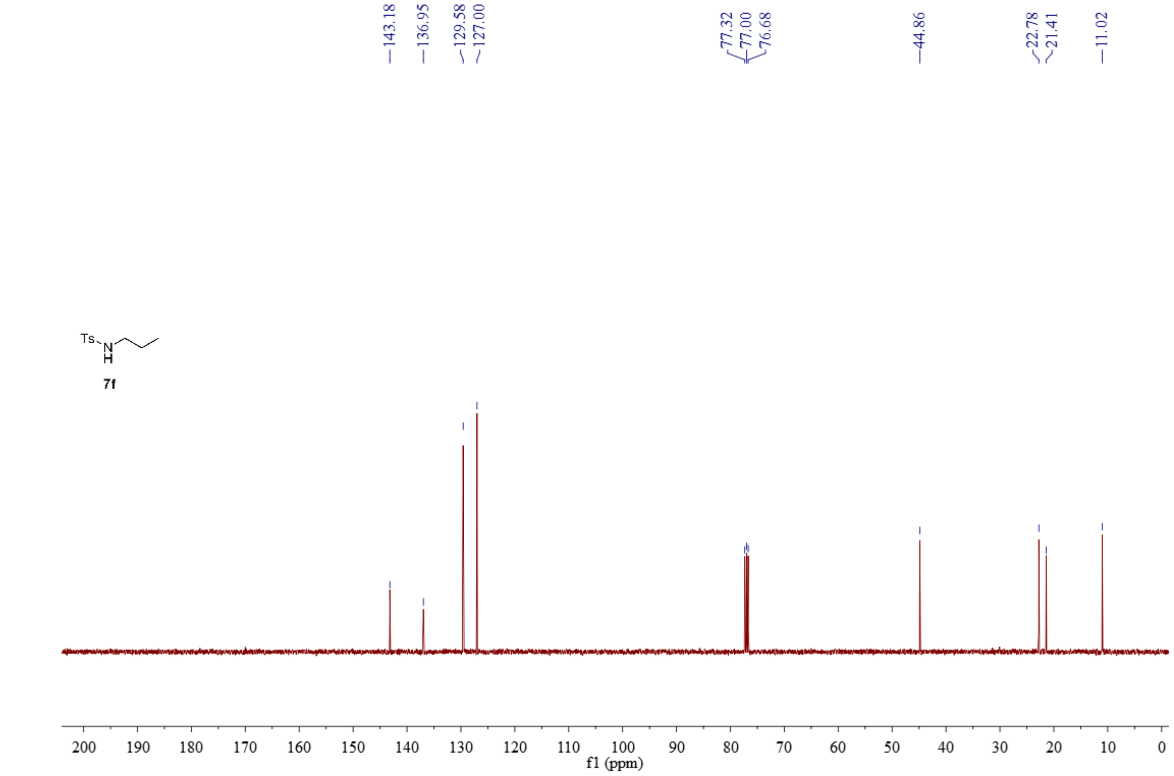
143.18
136.95
129.58
127.00
77.32
77.00
76.68
44.86
22.78
21.41
11.02
Ts
N
H
7f
200 190 180 170 160 150 140 130 120 110 100 90 80 70 60 50 40 30 20 10 0
f1 (ppm)

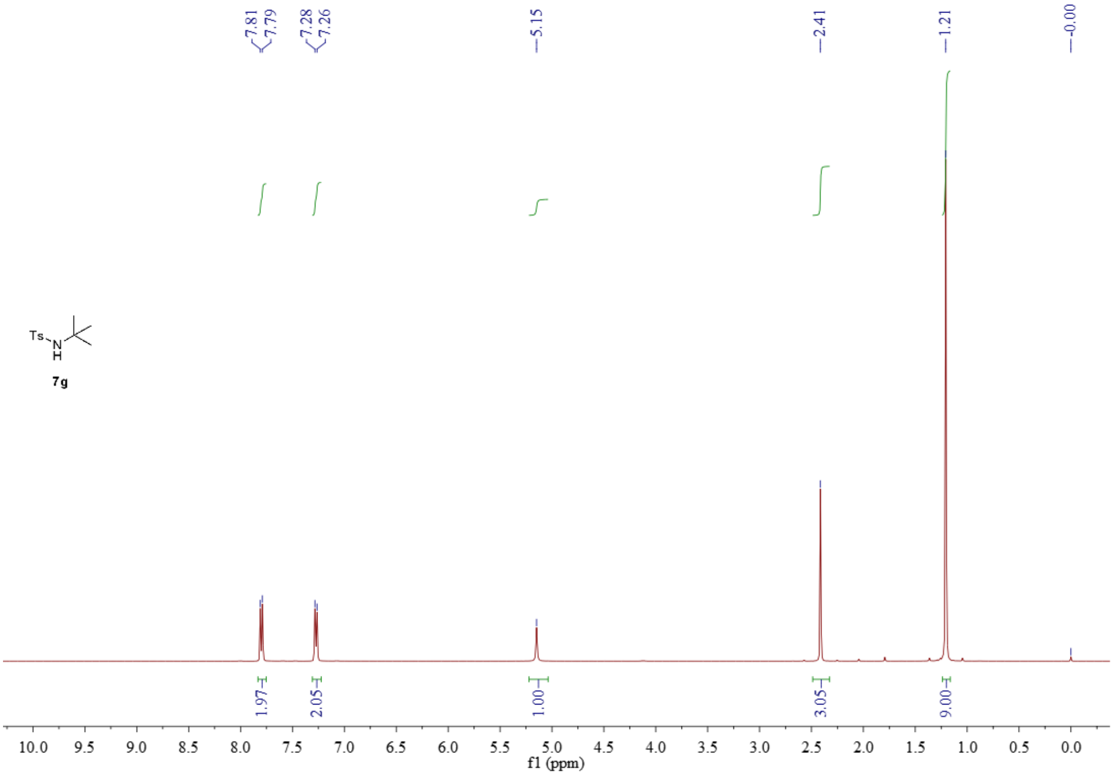
7.81
7.79
7.28
7.26
5.15
2.41
1.21
-0.00
Ts
N
H
7g
1.97
2.05
1.00
3.05
9.00
f1 (ppm)

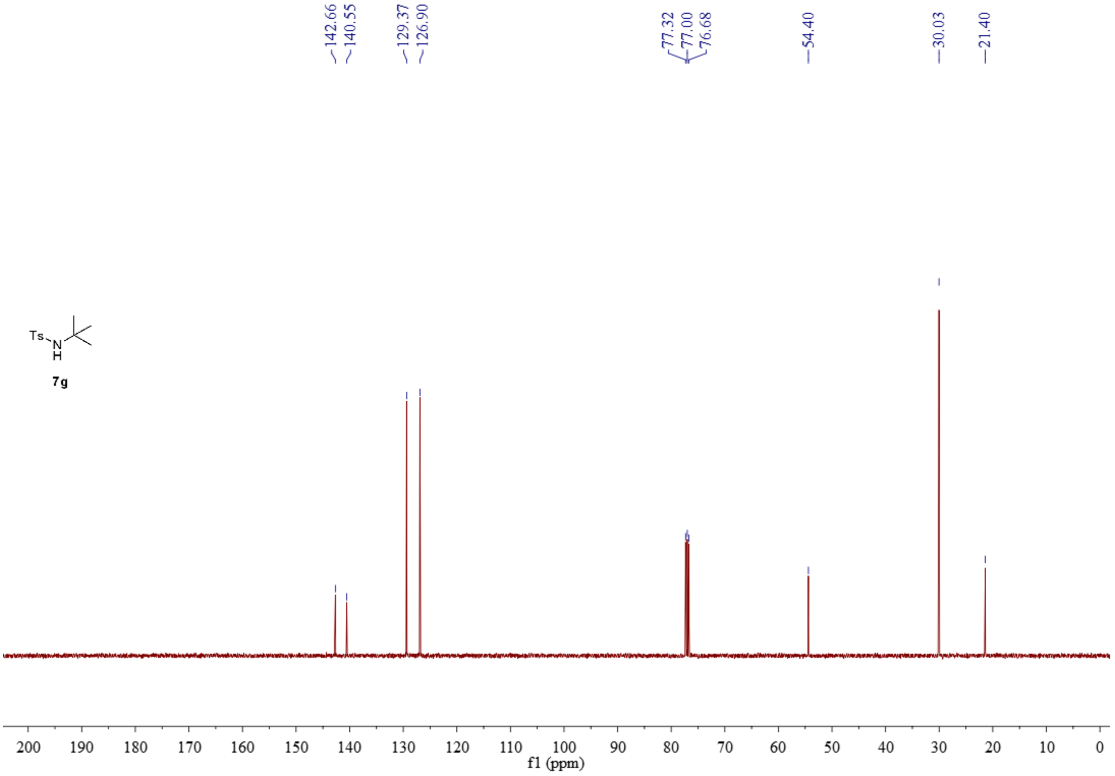
142.66
140.55
129.37
126.90
77.32
77.00
76.68
54.40
30.03
21.40
Ts
N
H
7g
f1 (ppm)

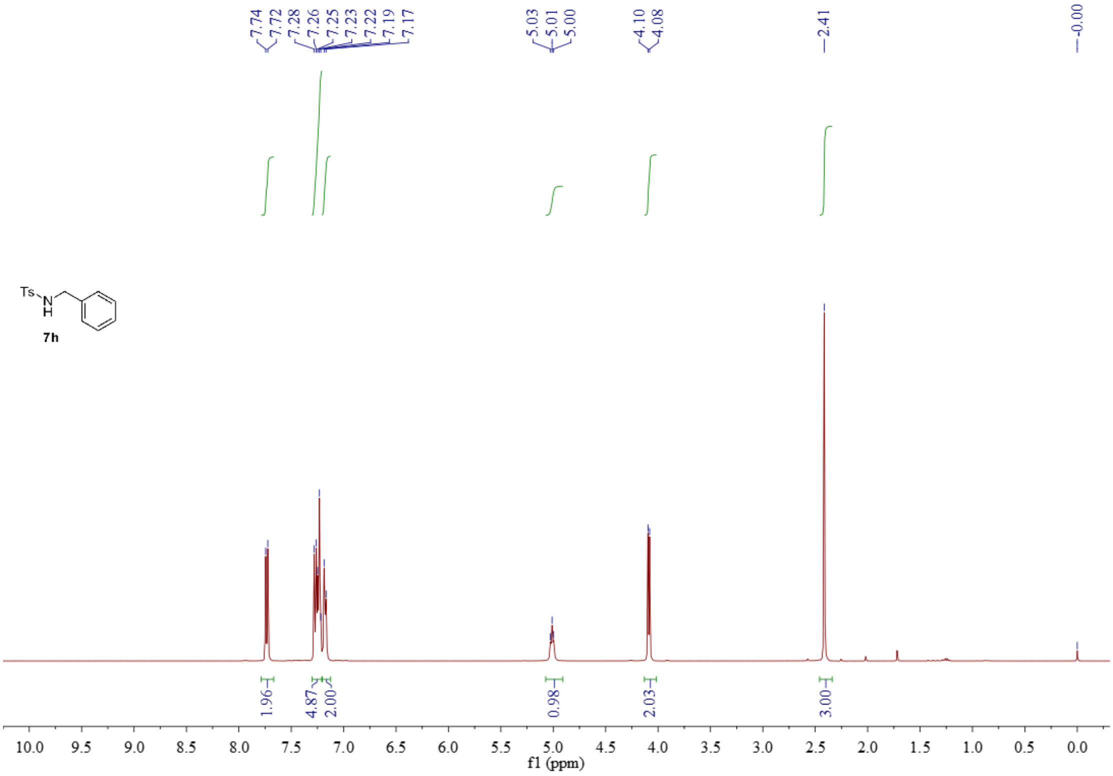
7.74
7.72
7.28
7.26
7.25
7.23
7.22
7.19
7.17
5.03
5.01
5.00
4.10
4.08
2.41
-0.00
Ts
N
H
7h
1.96
4.87
2.00
0.98
2.03
3.00
10.0 9.5 9.0 8.5 8.0 7.5 7.0 6.5 6.0 5.5 5.0 4.5 4.0 3.5 3.0 2.5 2.0 1.5 1.0 0.5 0.0
f1 (ppm)

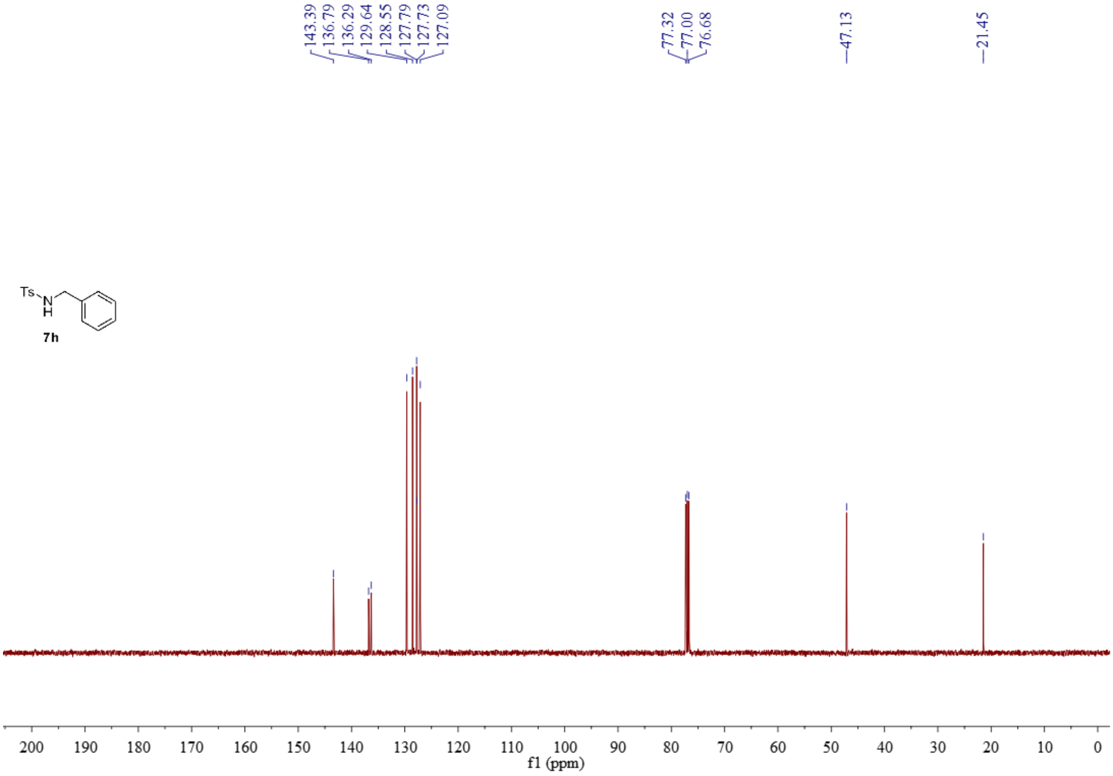
143.39
136.79
136.29
129.64
128.55
127.79
127.73
127.09
77.32
77.00
76.68
47.13
21.45
Ts
N
H
7h
200 190 180 170 160 150 140 130 120 110 100 90 80 70 60 50 40 30 20 10 0
f1 (ppm)

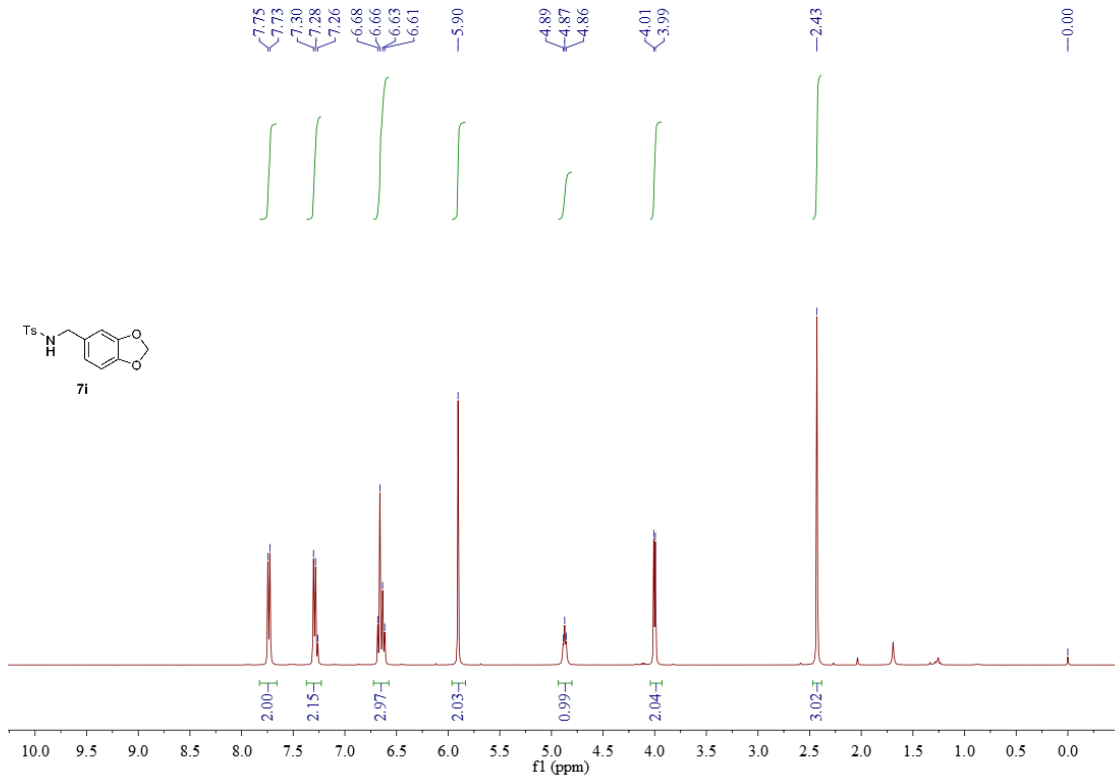
7.75
7.73
7.30
7.28
7.26
6.68
6.66
6.63
6.61
5.90
4.89
4.87
4.86
4.01
3.99
2.43
0.00
Ts
N
H
O
O
7i
2.00
2.15
2.97
2.03
0.99
2.04
3.02
10.0 9.5 9.0 8.5 8.0 7.5 7.0 6.5 6.0 5.5 5.0 4.5 4.0 3.5 3.0 2.5 2.0 1.5 1.0 0.5 0.0
f1 (ppm)

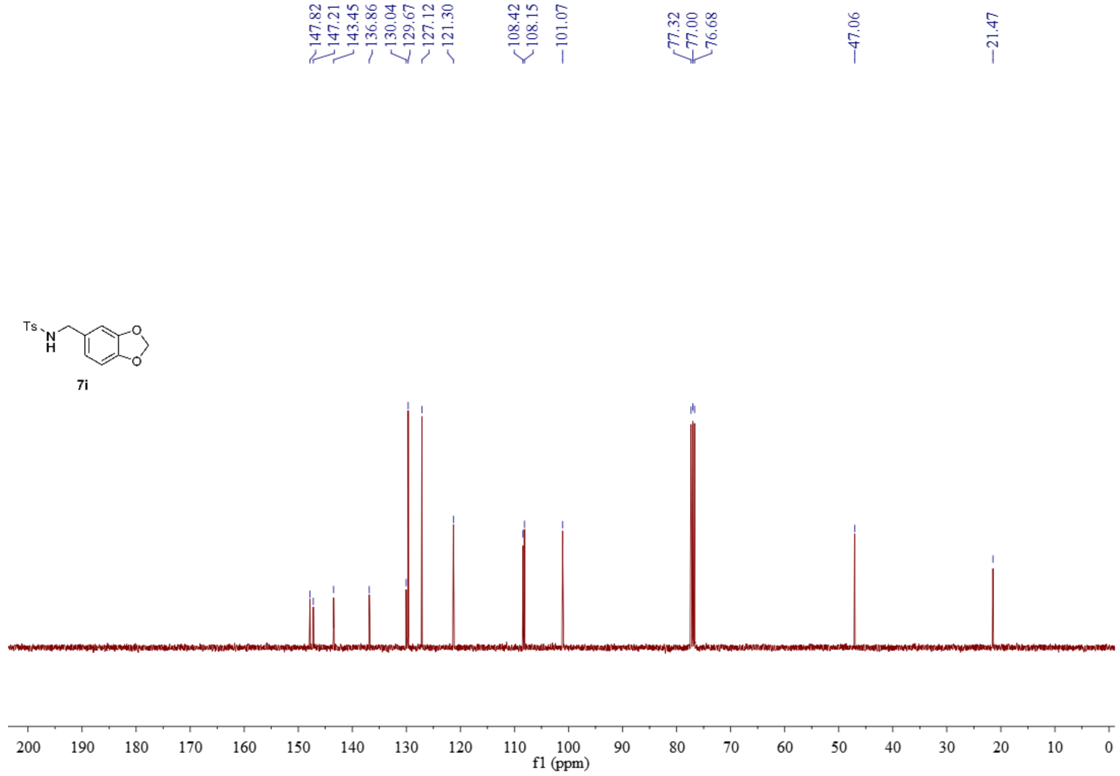
147.82
147.21
143.45
136.86
130.04
129.67
127.12
121.30
108.42
108.15
101.07
77.32
77.00
76.68
47.06
21.47
Ts
N
H
O
O
7i
200 190 180 170 160 150 140 130 120 110 100 90 80 70 60 50 40 30 20 10 0
f1 (ppm)

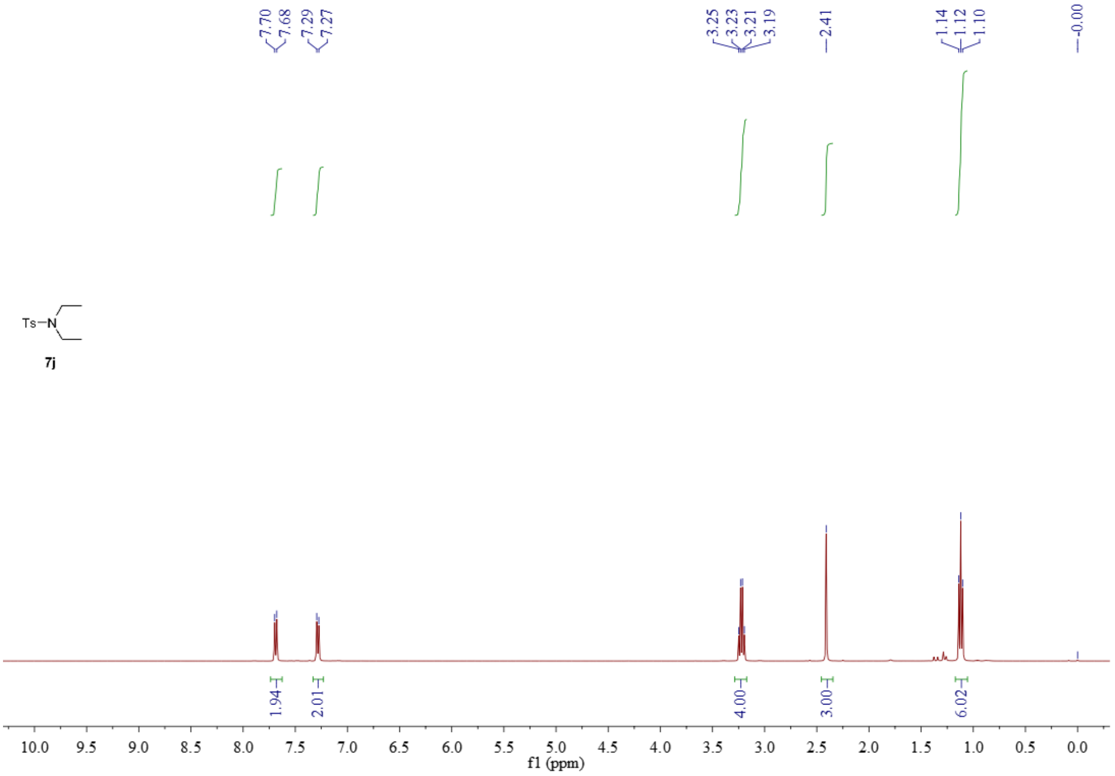
7.70
7.68
7.29
7.27
3.25
3.23
3.21
3.19
2.41
1.14
1.12
1.10
0.00
Ts–N
7j
1.94
2.01
4.00
3.00
6.02
10.0 9.5 9.0 8.5 8.0 7.5 7.0 6.5 6.0 5.5 5.0 4.5 4.0 3.5 3.0 2.5 2.0 1.5 1.0 0.5 0.0
f1 (ppm)

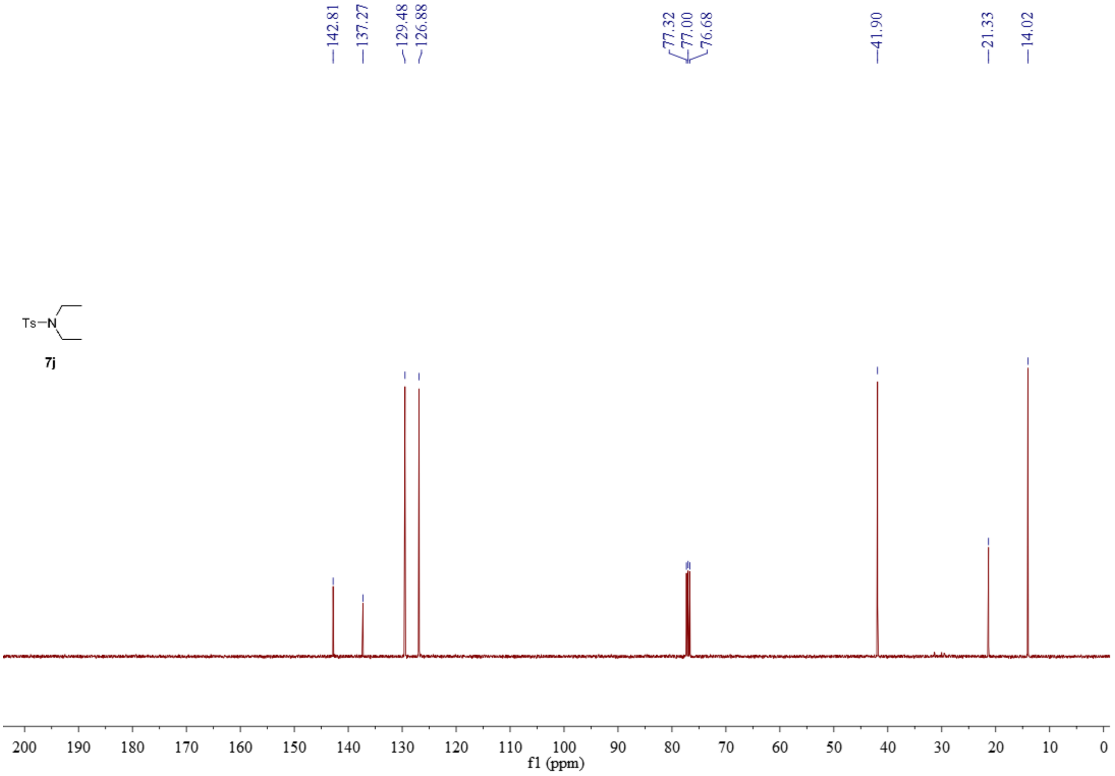
142.81
137.27
129.48
126.88
77.32
77.00
76.68
41.90
21.33
14.02
Ts–N
7j
200 190 180 170 160 150 140 130 120 110 100 90 80 70 60 50 40 30 20 10 0
f1 (ppm)

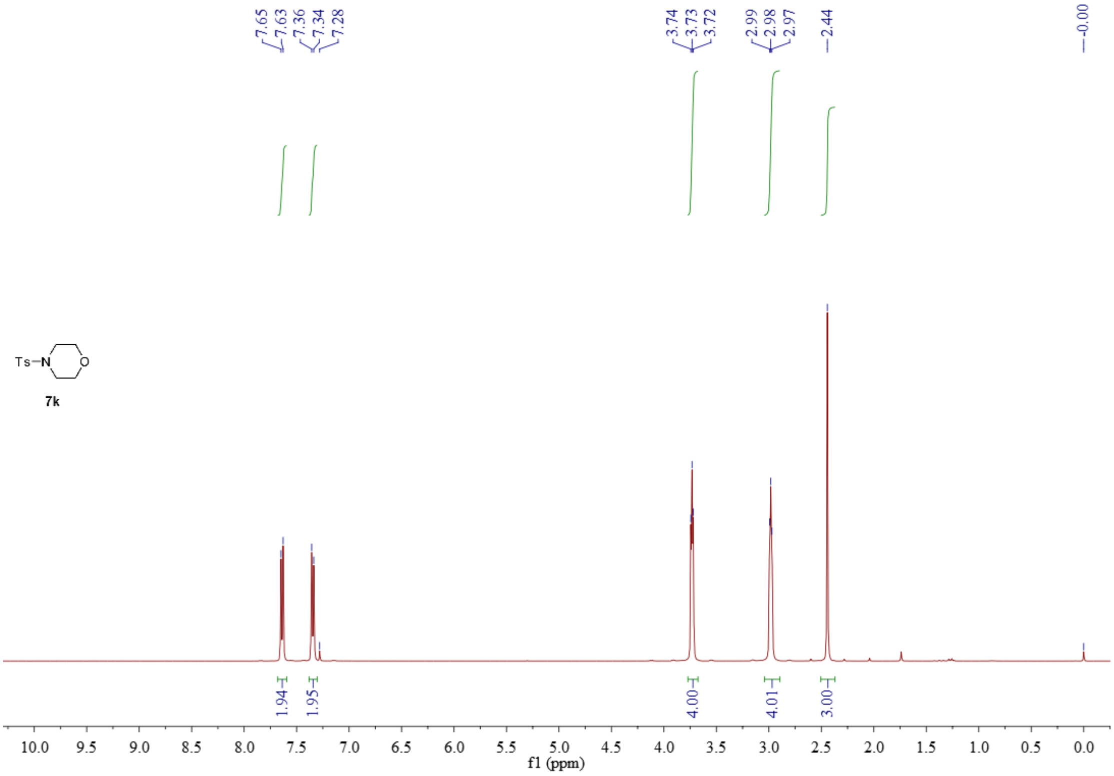
Ts–N O
7.65
7.63
7.36
7.34
7.28
3.74
3.73
3.72
2.99
2.98
2.97
2.44
-0.00
1.94
1.95
4.00
4.01
3.00
10.0 9.5 9.0 8.5 8.0 7.5 7.0 6.5 6.0 5.5 5.0 4.5 4.0 3.5 3.0 2.5 2.0 1.5 1.0 0.5 0.0
f1 (ppm)

7k

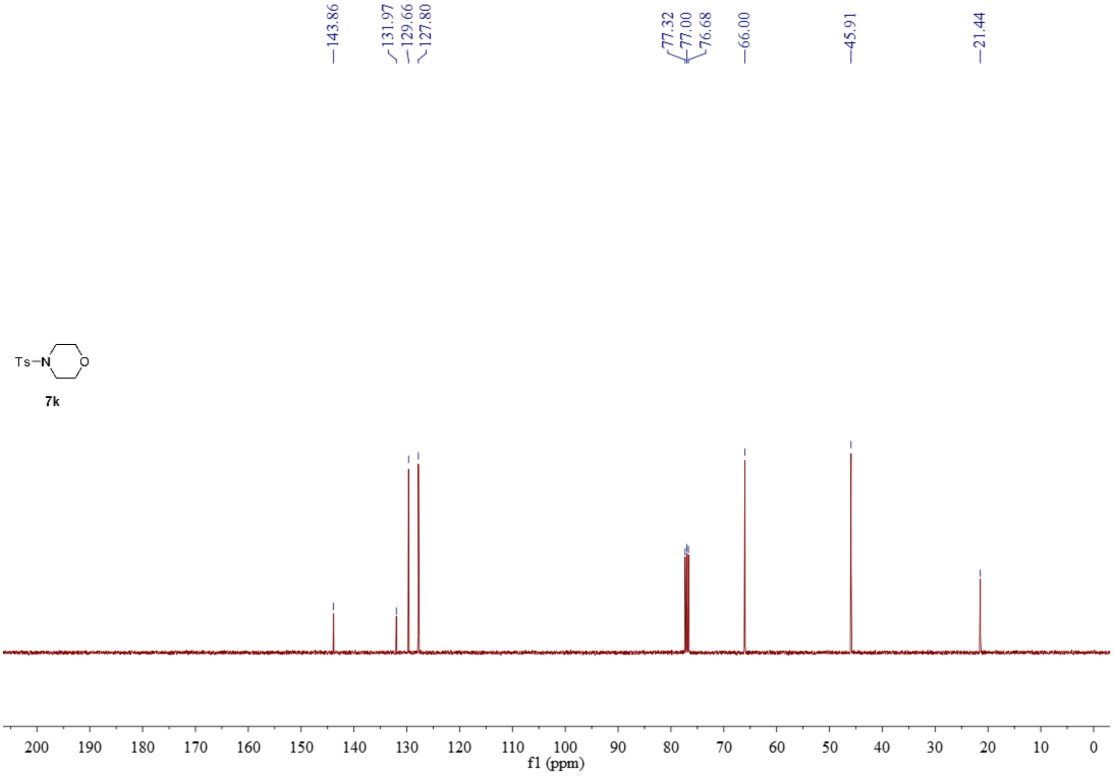
Ts–N O
143.86
131.97
129.66
127.80
77.32
77.00
76.68
66.00
45.91
21.44
200 190 180 170 160 150 140 130 120 110 100 90 80 70 60 50 40 30 20 10 0
f1 (ppm)

7k

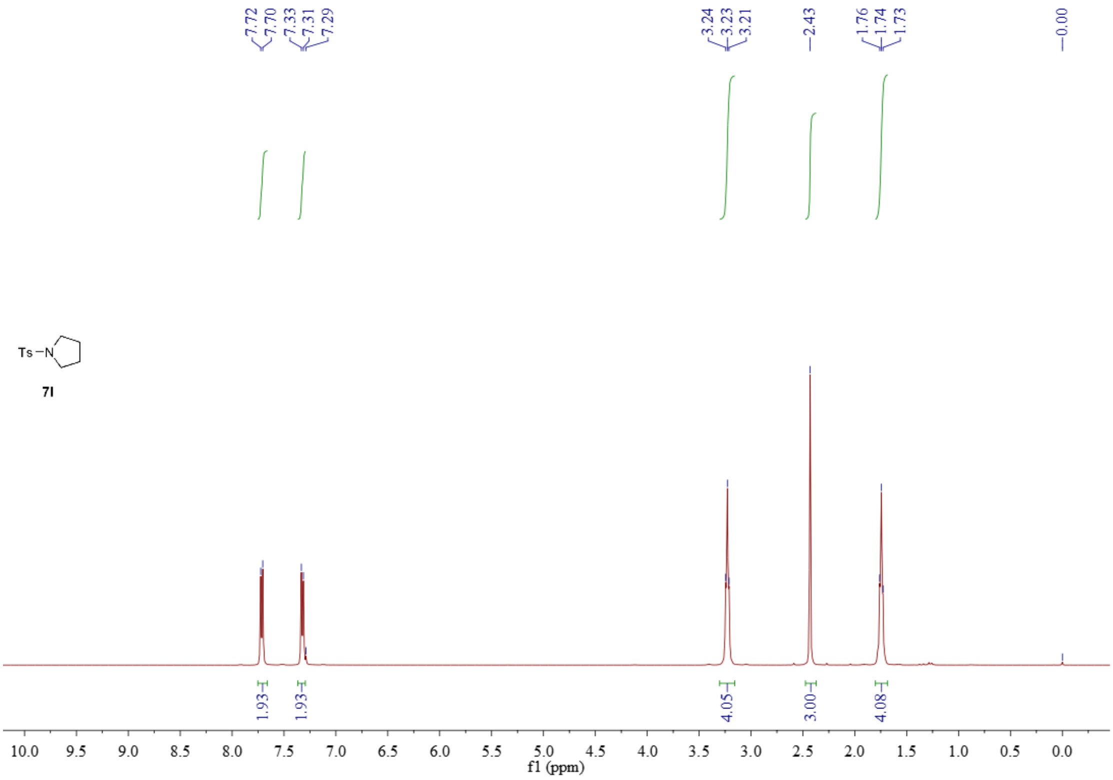

7.72
7.70
7.33
7.31
7.29
3.24
3.23
3.21
2.43
1.76
1.74
1.73
0.00
Ts–N
7l
1.93
1.93
4.05
3.00
4.08
10.0 9.5 9.0 8.5 8.0 7.5 7.0 6.5 6.0 5.5 5.0 4.5 4.0 3.5 3.0 2.5 2.0 1.5 1.0 0.5 0.0
f1 (ppm)

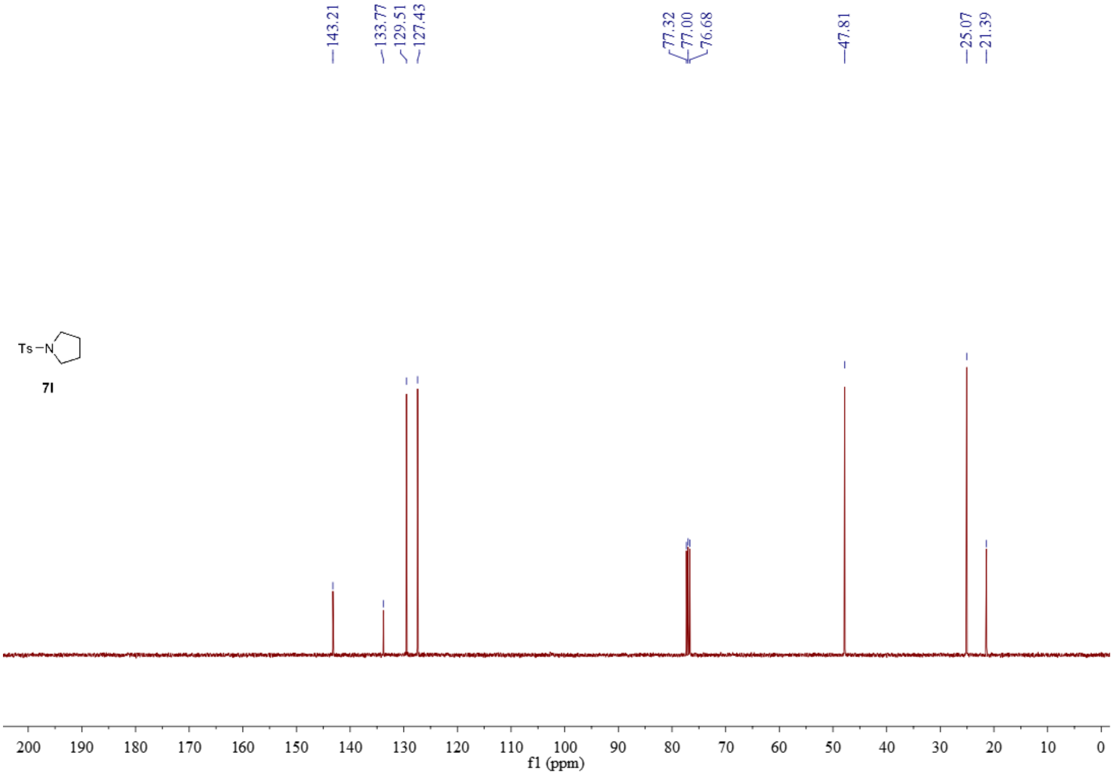

143.21
133.77
129.51
127.43
77.32
77.00
76.68
47.81
25.07
21.39
Ts–N
7l
200 190 180 170 160 150 140 130 120 110 100 90 80 70 60 50 40 30 20 10 0
f1 (ppm)

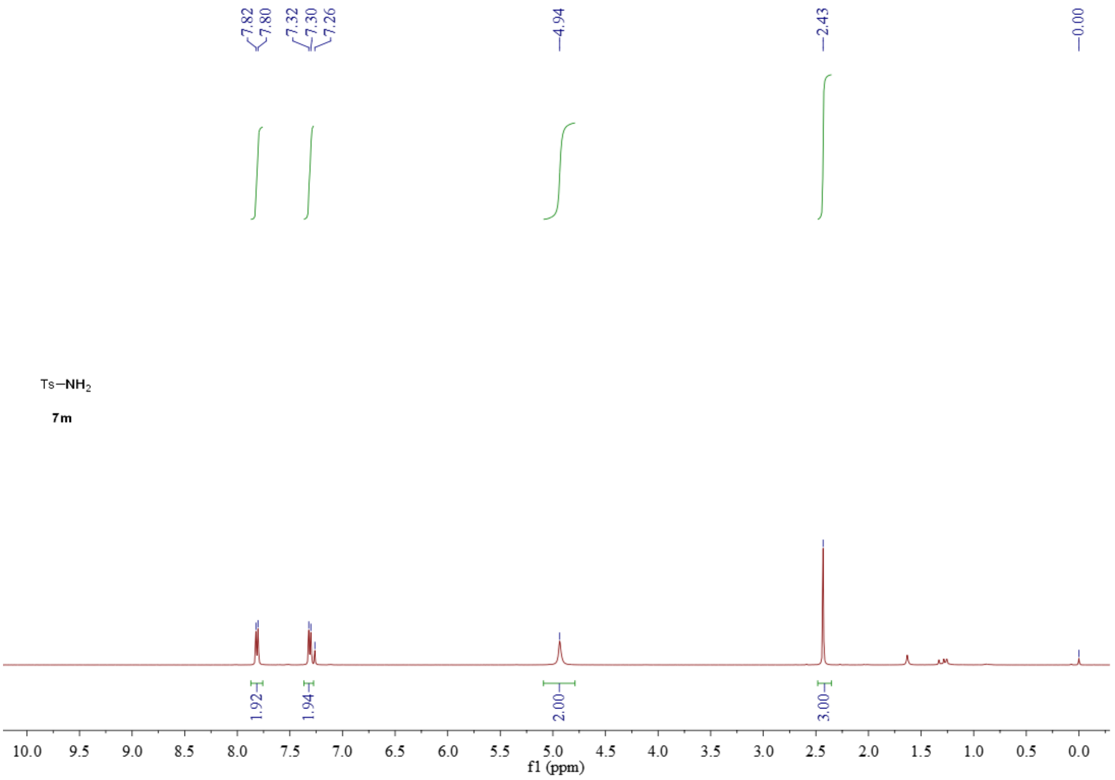
7.82
7.80
7.32
7.30
7.26
4.94
2.43
0.00
Ts−NH2
7m
1.92
1.94
2.00
3.00
10.0 9.5 9.0 8.5 8.0 7.5 7.0 6.5 6.0 5.5 5.0 4.5 4.0 3.5 3.0 2.5 2.0 1.5 1.0 0.5 0.0
f1 (ppm)

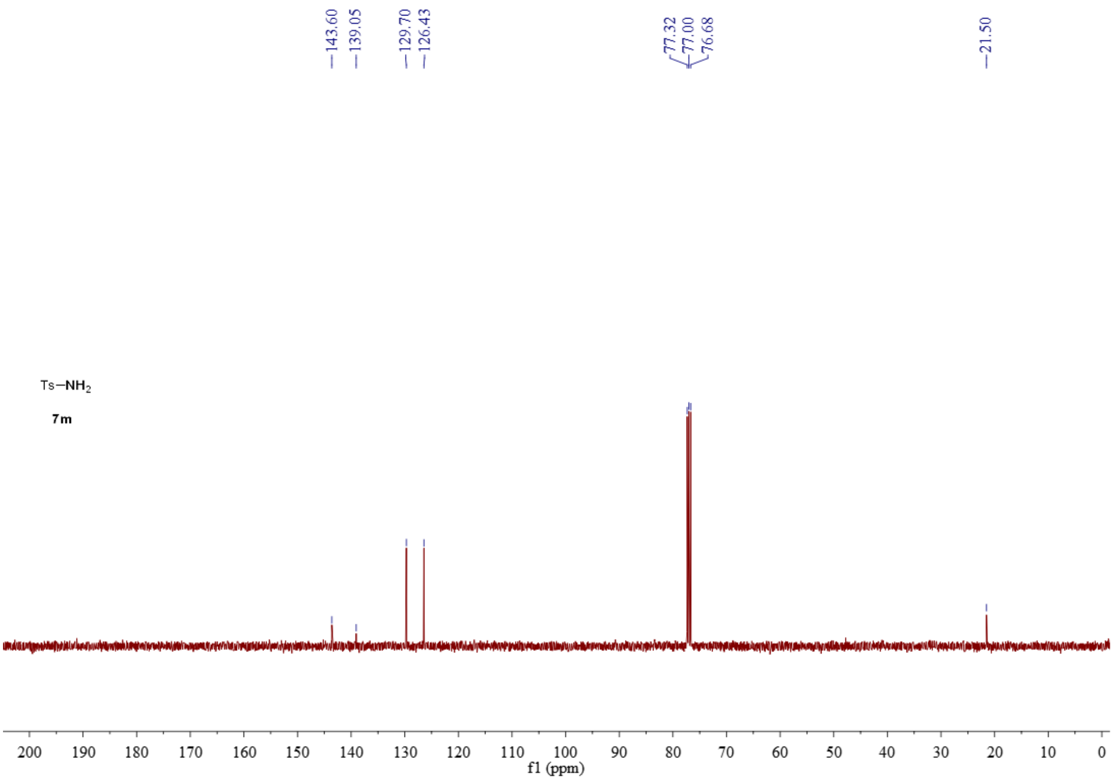
143.60
139.05
129.70
126.43
77.32
77.00
76.68
21.50
Ts−NH2
7m
200 190 180 170 160 150 140 130 120 110 100 90 80 70 60 50 40 30 20 10 0
f1 (ppm)

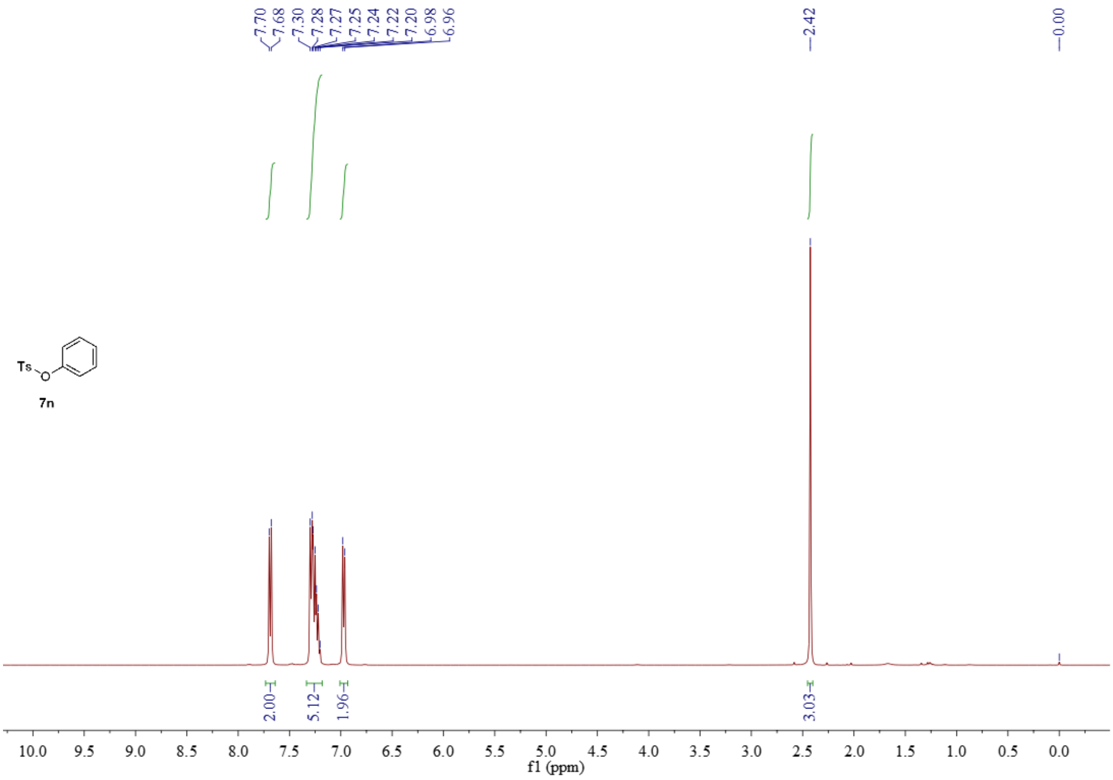
7.70
7.68
7.30
7.28
7.27
7.25
7.24
7.22
7.20
6.98
6.96
2.42
0.00
Ts
O
7n
2.00
5.12
1.96
3.03
10.0 9.5 9.0 8.5 8.0 7.5 7.0 6.5 6.0 5.5 5.0 4.5 4.0 3.5 3.0 2.5 2.0 1.5 1.0 0.5 0.0
f1 (ppm)

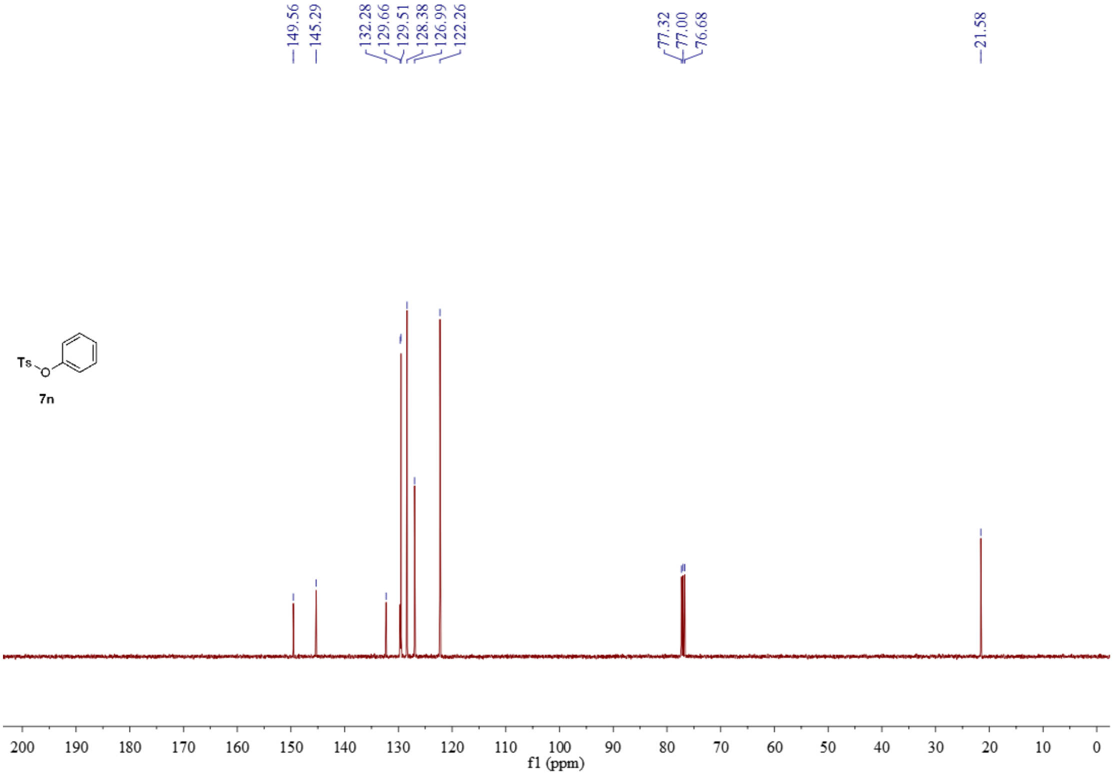
149.56
145.29
132.28
129.66
129.51
128.38
126.99
122.26
77.32
77.00
76.68
21.58
Ts
O
7n
200 190 180 170 160 150 140 130 120 110 100 90 80 70 60 50 40 30 20 10 0
f1 (ppm)

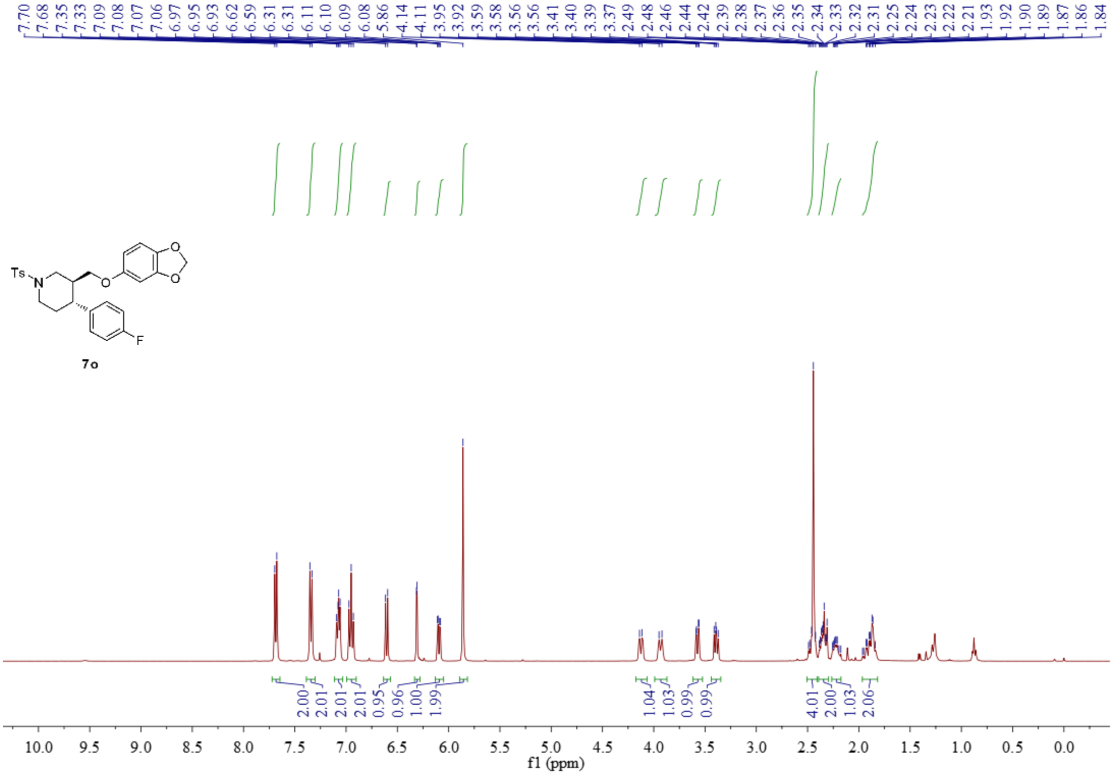
7.70 7.68 7.35 7.33 7.09 7.08 7.07 7.06 6.97 6.95 6.93 6.62 6.59 6.31 6.31 6.11 6.10 6.09 6.08 5.86 4.14 4.11 3.95 3.92 3.59 3.58 3.56 3.56 3.41 3.40 3.39 3.37 2.49 2.48 2.46 2.44 2.42 2.39 2.38 2.37 2.36 2.35 2.34 2.33 2.32 2.31 2.25 2.24 2.23 2.22 2.21 1.93 1.92 1.90 1.89 1.87 1.86 1.84
Ts
N
O
O
O
F
7o
2.00 2.01 2.01 2.01 0.95 0.96 1.00 1.99 1.04 1.03 0.99 0.99 4.01 2.00 1.03 2.06
10.0 9.5 9.0 8.5 8.0 7.5 7.0 6.5 6.0 5.5 5.0 4.5 4.0 3.5 3.0 2.5 2.0 1.5 1.0 0.5 0.0
f1 (ppm)

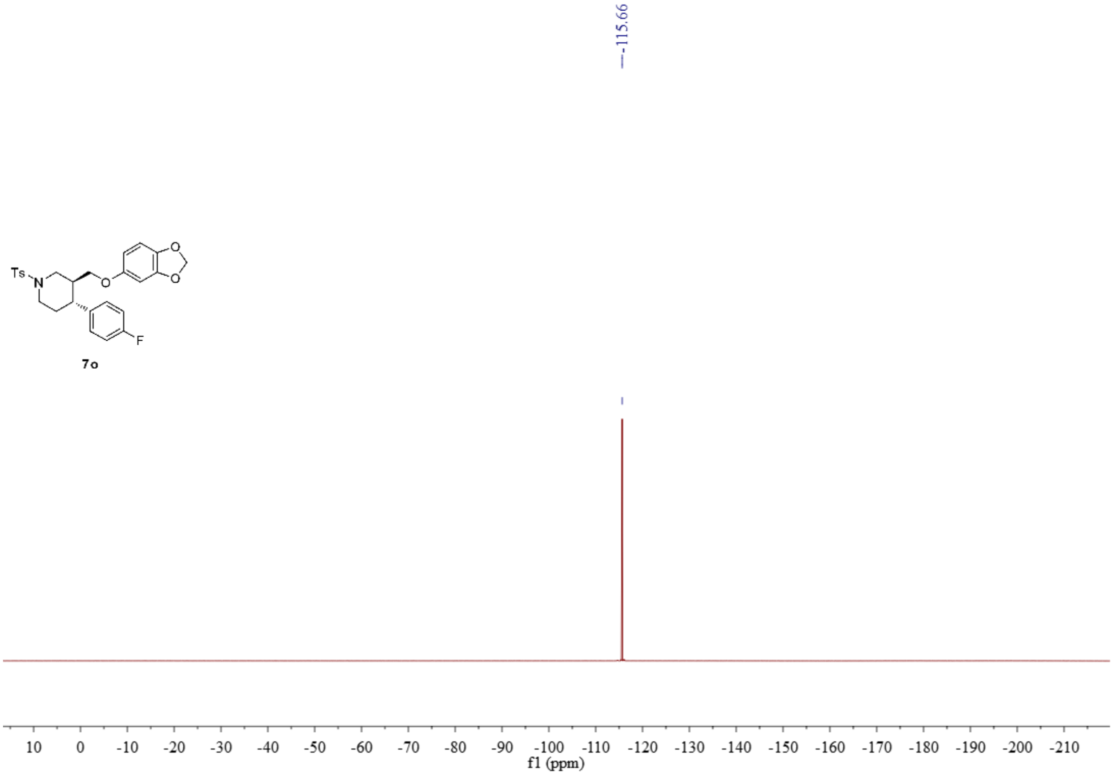
-115.66
Ts
N
O
O
O
F
7o
10 0 -10 -20 -30 -40 -50 -60 -70 -80 -90 -100 -110 -120 -130 -140 -150 -160 -170 -180 -190 -200 -210
f1 (ppm)

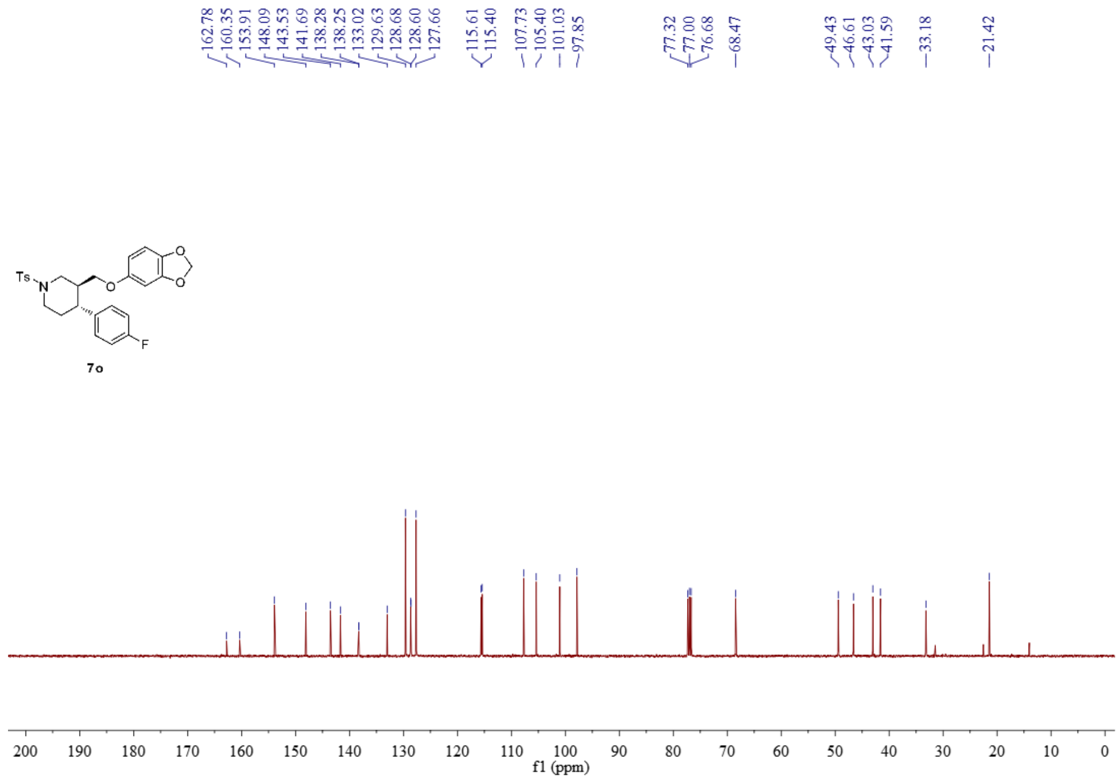
162.78
160.35
153.91
148.09
143.53
141.69
138.28
138.25
133.02
129.63
128.68
128.60
127.66
115.61
115.40
107.73
105.40
101.03
97.85
77.32
77.00
76.68
68.47
49.43
46.61
43.03
41.59
33.18
21.42
Ts
N
O
O
O
F
7o
200 190 180 170 160 150 140 130 120 110 100 90 80 70 60 50 40 30 20 10 0
f1 (ppm)

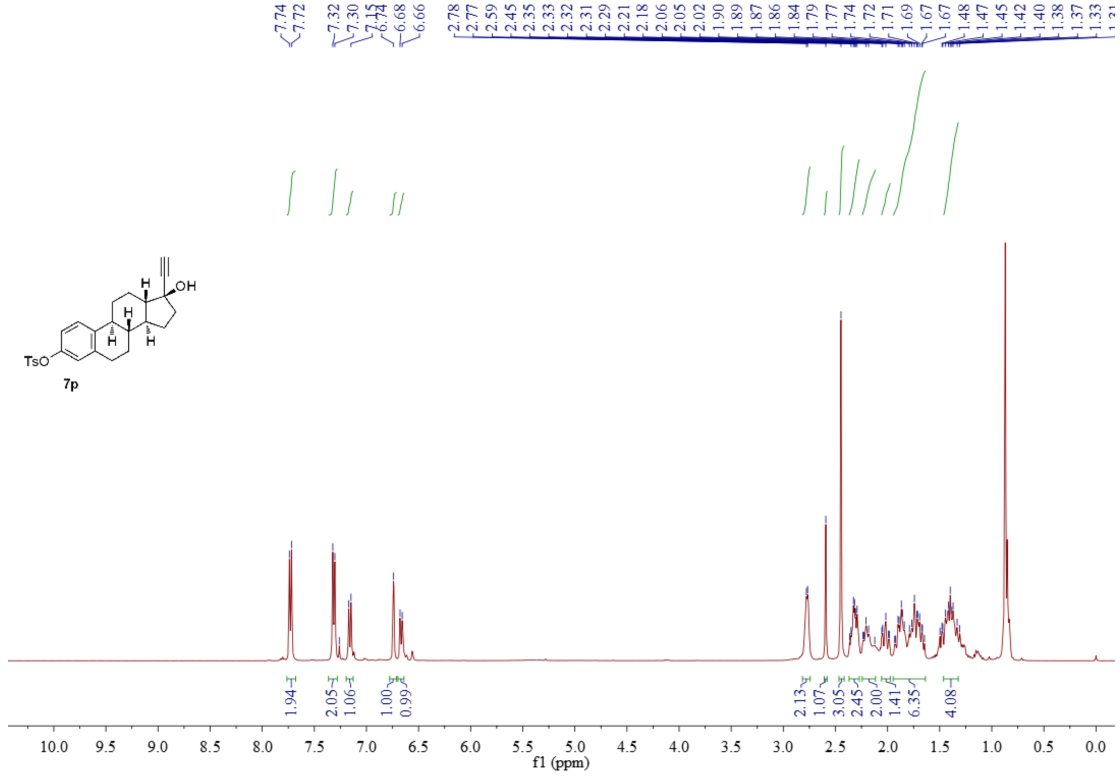
7.74
7.72
7.32
7.30
7.15
7.14
6.74
6.68
6.66
2.78
2.77
2.59
2.45
2.35
2.33
2.32
2.31
2.29
2.21
2.18
2.06
2.05
2.02
1.90
1.89
1.87
1.86
1.84
1.79
1.77
1.74
1.72
1.71
1.69
1.67
1.67
1.48
1.47
1.45
1.42
1.40
1.38
1.37
1.33
OH
H
H
H
TsO
7p
1.94
2.05
1.06
1.00
0.99
2.13
1.07
3.05
2.45
2.00
1.41
6.35
4.08
10.0 9.5 9.0 8.5 8.0 7.5 7.0 6.5 6.0 5.5 5.0 4.5 4.0 3.5 3.0 2.5 2.0 1.5 1.0 0.5 0.0
f1 (ppm)

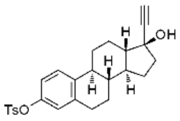

7p

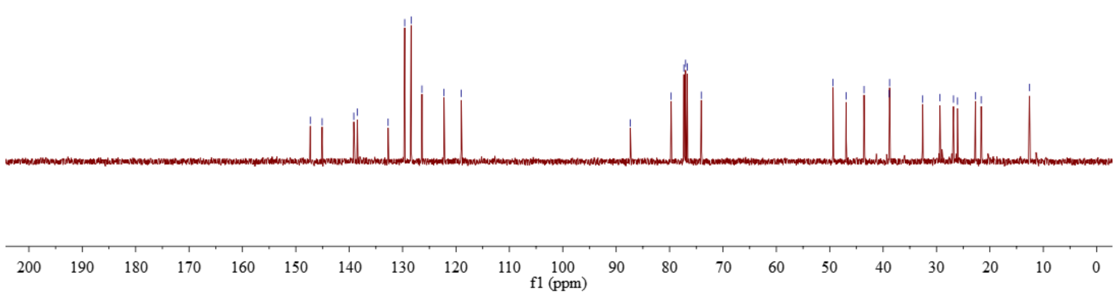

147.29
145.07
139.14
138.49
132.71
129.61
128.40
126.38
122.25
119.01
87.35
79.70
77.32
77.00
76.68
74.06
49.38
46.93
43.57
38.84
38.79
32.59
29.35
26.82
26.06
22.71
21.63
12.59
200 190 180 170 160 150 140 130 120 110 100 90 80 70 60 50 40 30 20 10 0
f1 (ppm)
